# Supplementary material for: Inventory of Patient-Reported Outcome Measures Used in the Non-Operative Care of Scoliosis: A Scoping Review
Source: Children (Basel). 2023 Jan 29;10(2):239. doi: 10.3390/children10020239 (PMC9954663; doi:10.3390/children10020239)
Supplement: Supplementary file 1 [file children-10-00239-s001.zip › S2 Edited references.pdf]

Covidence	Study	Reference
#56	Sponseller 1987	Sponseller, P D; Cohen, M S; Nachemson, A L; Hall, J E; Wohl, M E. Results of surgical treatment of adults with idiopathic scoliosis. The Journal of bone and joint surgery. American volume, 1987, 69, 5, 667-75. .
#74	MacLean 1989	MacLean, W E Jr; Green, N E; Pierre, C B; Ray, D C. Stress and coping with scoliosis: psychological effects on adolescents and their families. Journal of pediatric orthopedics, 1989, 9, 3, 257-61. .
#131	Cordover 1997	Cordover, A M; Betz, R R; Clements, D H; Bosacco, S J. Natural history of adolescent thoracolumbar and lumbar idiopathic scoliosis into adulthood. Journal of spinal disorders, 1997, 10, 3, 193-6. .
#206	Asher 2002	Asher, Marc; Lai, Sue Min; Burton, Doug; Manna, Barbara. Spine deformity correlates better than trunk deformity with idiopathic scoliosis patients' quality of life questionnaire responses. Studies in health technology and informatics, 2002, 91, ck1, 9214582, 462-4. .
#229	Danielsson 2003	Danielsson, Aina J; Nachemson, Alf L. Back pain and function 22 years after brace treatment for adolescent idiopathic scoliosis: a case-control study-part I. Spine, 2003, 28, 18, 2078-2086. .
#234	Sanders 2003	Sanders, James O; Polly, David W Jr; Cats-Baril, William; Jones, JoAnn; Lenke, Larry G; O'Brien, Michael F; Stephens Richards, B; Sucato, Daniel J; AIS Section of the Spinal Deformity Study Group. Analysis of patient and parent assessment of deformity in idiopathic scoliosis using the Walter Reed Visual Assessment Scale. Spine, 2003, 28, 18, 2158-63. .
#315	Weigert 2006	Weigert, Karen Petra; Nygaard, Linda Marie; Christensen, Finn Bjarke; Hansen, Ebbe Stender; Bunger, Cody. Outcome in adolescent idiopathic scoliosis after brace treatment and surgery assessed by means of the Scoliosis Research Society Instrument 24. European spine journal : official publication of the European Spine Society, the European Spinal Deformity Society, and the European Section of the Cervical Spine Research Society, 2006, 15, 7, 1108-17. .

#321	Andersen 2006	Andersen, Mikkel O; Christensen, Steen B; Thomsen, Karsten. Outcome at 10 years after treatment for adolescent idiopathic scoliosis. Spine, 2006, 31, 3, 350-4. .
#322	Haefeli 2006	Haefeli, Mathias; Elfering, Achim; Kilian, Reinhold; Min, Kan; Boos, Norbert. Nonoperative treatment for adolescent idiopathic scoliosis: a 10- to 60-year follow-up with special reference to health-related quality of life. Spine, 2006, 31, 3, 355-367. .
#328	Glassman 2006	Glassman, Steven D; Berven, Sigurd; Kostuik, John; Dimar, John R; Horton, William C; Bridwell, Keith. Nonsurgical resource utilization in adult spinal deformity. Spine, 2006, 31, 8, 941-7. .
#335	Beka 2006	Beka, Anastasia; Dermitzaki, Irini; Christodoulou, Anastasios; Kapetanos, George; Markovitis, Marios; Pournaras, John. Children and adolescents with idiopathic scoliosis: emotional reactions, coping mechanisms, and self-esteem. Psychological reports, 2006, 98, 2, 477-85. .
#353	Crawford 2006	Crawford, John R; Izatt, Maree T; Adam, Clayton J; Labrom, Robert D; Askin, Geoffrey N. A prospective assessment of SRS-24 scores after endoscopic anterior instrumentation for scoliosis. Spine, 2006, 31, 21, E817-22. .
#363	Vasiliadis 2006	Vasiliadis, Elias; Grivas, Theodoros B; Savvidou, Olga; Triantafyllopoulos, Georgios. The influence of brace on quality of life of adolescents with idiopathic scoliosis. Studies in health technology and informatics, 2006, 123, ck1, 9214582, 352-6. .
#364	Parent 2006	Parent, E C; Hill, D; Mahood, J; Moreau, M; Lou, E; Raso, J. Associations between quality-of-life and internal or external spinal deformity measurements in adolescent with idiopathic scoliosis (AIS). Studies in health technology and informatics, 2006, 123, ck1, 9214582, 357-63. .
#377	Bunge 2007	Bunge, Eveline M; Juttmann, Rikard E; de Kleuver, Marinus; van Biezen, Frans C; de Koning, Harry J; NESCIO group. Health-related quality of life in patients with adolescent idiopathic scoliosis after treatment: short-term effects after brace or surgical treatment. European spine journal : official publication of the European Spine Society, the European Spinal Deformity Society, and the European Section of the Cervical Spine Research Society, 2007, 16, 1, 30560. .

#393	Kluba 2009	Kluba, Torsten; Dikmenli, Gulden; Dietz, Klaus; Giehl, Johannes P; Niemeyer, Thomas. Comparison of surgical and conservative treatment for degenerative lumbar scoliosis. Archives of orthopaedic and trauma surgery, 2009, 129, 1, 44566. https://dx.doi.org/10.1007/s00402-008-0673-z.
#398	Lerner 2009	Lerner, Thomas; Bullmann, Viola; Schulte, Tobias L; Schneider, Marc; Liljenqvist, Ulf. A level-1 pilot study to evaluate of ultraporous beta-tricalcium phosphate as a graft extender in the posterior correction of adolescent idiopathic scoliosis. European spine journal : official publication of the European Spine Society, the European Spinal Deformity Society, and the European Section of the Cervical Spine Research Society, 2009, 18, 2, 170-9. https://dx.doi.org/10.1007/s00586-008-0844-1.
#409	Lonner 2009	Lonner, Baron S; Auerbach, Joshua D; Estreicher, Michael; Milby, Andrew H; Kean, Kristin E; Panagopoulos, Georgia; Chang, David. Video-assisted anterior thoracoscopic spinal fusion versus posterior spinal fusion: a comparative study utilizing the SRS-22 outcome instrument. Spine, 2009, 34, 2, 193-8. https://dx.doi.org/10.1097/BRS.0b013e318191e9e8.
#410	Rivett 2009	Rivett, LouAnn; Rothberg, Alan; Stewart, Aimee; Berkowitz, Rowan. The relationship between quality of life and compliance to a brace protocol in adolescents with idiopathic scoliosis: a comparative study. BMC musculoskeletal disorders, 2009, 10, 100968565, 5. https://dx.doi.org/10.1186/1471-2474-10-5.
#411	Lonner 2009	Lonner, Baron S; Auerbach, Joshua D; Estreicher, Michael; Milby, Andrew H; Kean, Kristin E. Video-assisted thoracoscopic spinal fusion compared with posterior spinal fusion with thoracic pedicle screws for thoracic adolescent idiopathic scoliosis. The Journal of bone and joint surgery. American volume, 2009, 91, 2, 398-408. https://dx.doi.org/10.2106/JBJS.G.01044.
#421	Beausejour 2009	Beausejour, Marie; Joncas, Julie; Goulet, Lise; Roy-Beaudry, Marjolaine; Parent, Stefan; Grimard, Guy; Forcier, Martin; Lauriault, Sophie; Labelle, Hubert. Reliability and validity of adapted French Canadian version of Scoliosis Research Society Outcomes Questionnaire (SRS-22) in Quebec. Spine, 2009, 34, 6, 623-8. https://dx.doi.org/10.1097/BRS.0b013e3181973e58.
#422	Liao 2009	Liao, Jen-Chung; Chen, Wen-Jer; Chen, Lih-Huei; Niu, Chi-Chien. Outcome of the L5-S1 segment after posterior instrumented spinal surgery in degenerative lumbar diseases. Chang Gung medical journal, 2009, 32, 1, 29799. .
#426	Newton 2009	Newton, Peter O; Upasani, Vidyadhar V; Bastrom, Tracey P; Marks, Michelle C. The deformity-flexibility quotient predicts both patient satisfaction and surgeon preference in the treatment of Lenke 1B or 1C curves for adolescent idiopathic scoliosis. Spine, 2009, 34, 10, 1032-9. https://dx.doi.org/10.1097/BRS.0b013e31819c97f8.

#427	Pekmezci 2009	Pekmezci, Murat; Berven, Sigurd H; Hu, Serena S; Deviren, Vedat. The factors that play a role in the decision-making process of adult deformity patients. Spine, 2009, 34, 8, 813-7. https://dx.doi.org/10.1097/BRS.0b013e3181851ba6.
#450	Bago 2009	Bago, Juan; Perez-Grueso, Francisco J S; Les, Esther; Hernandez, Pablo; Pellise, Ferran. Minimal important differences of the SRS-22 Patient Questionnaire following surgical treatment of idiopathic scoliosis. European spine journal : official publication of the European Spine Society, the European Spinal Deformity Society, and the European Section of the Cervical Spine Research Society, 2009, 18, 12, 1898-904. https://dx.doi.org/10.1007/s00586-009-1066-x.
#452	Mac-Thiong 2009	Mac-Thiong, Jean-Marc; Transfeldt, Ensor E; Mehbod, Amir A; Perra, Joseph H; Denis, Francis; Garvey, Timothy A; Lonstein, John E; Wu, Chunhui; Dorman, Christopher W; Winter, Robert B. Can c7 plumbline and gravity line predict health related quality of life in adult scoliosis?. Spine, 2009, 34, 15, E519-27. https://dx.doi.org/10.1097/BRS.0b013e3181a9c7ad.
#454	Ploumis 2009	Ploumis, Avraam; Liu, Hong; Mehbod, Amir A; Transfeldt, Ensor E; Winter, Robert B. A correlation of radiographic and functional measurements in adult degenerative scoliosis. Spine, 2009, 34, 15, 1581-4. https://dx.doi.org/10.1097/BRS.0b013e31819c94cc.
#456	Smith 2009	Smith, Justin S; Shaffrey, Christopher I; Berven, Sigurd; Glassman, Steven; Hamill, Christopher; Horton, William; Ondra, Stephen; Schwab, Frank; Shainline, Michael; Fu, Kai-Ming; Bridwell, Keith; Spinal Deformity Study Group. Improvement of back pain with operative and nonoperative treatment in adults with scoliosis. Neurosurgery, 2009, 65, 1, 31503. https://dx.doi.org/10.1227/01.NEU.0000347005.35282.6C.
#459	Lafage 2009	Lafage, Virginie; Schwab, Frank; Patel, Ashish; Hawkinson, Nicola; Farcy, Jean-Pierre. Pelvic tilt and truncal inclination: two key radiographic parameters in the setting of adults with spinal deformity. Spine, 2009, 34, 17, E599-606. https://dx.doi.org/10.1097/BRS.0b013e3181aad219.
#460	Misterska 2009	Misterska, Ewa; Glowacki, Maciej; Harasymczuk, Jerzy. Polish adaptation of Bad Sobernheim Stress Questionnaire-Brace and Bad Sobernheim Stress Questionnaire-Deformity. European spine journal : official publication of the European Spine Society, the European Spinal Deformity Society, and the European Section of the Cervical Spine Research Society, 2009, 18, 12, 4262. https://dx.doi.org/10.1007/s00586-009-1126-2.

#463	Tsutsui 2009	Tsutsui, Shunji; Pawelek, Jeff; Bastrom, Tracey; Lenke, Lawrence; Lowe, Thomas; Betz, Randal; Clements, David; Newton, Peter O. Dissecting the effects of spinal fusion and deformity magnitude on quality of life in patients with adolescent idiopathic scoliosis. Spine, 2009, 34, 18, E653-8. https://dx.doi.org/10.1097/BRS.0b013e3181b2008f.
#472	Li 2009	Li, Gang; Passias, Peter; Kozanek, Michal; Fu, Eric; Wang, Shaobai; Xia, Qun; Li, Guoan; Rand, Frank E; Wood, Kirkham B. Adult scoliosis in patients over sixty-five years of age: outcomes of operative versus nonoperative treatment at a minimum two-year follow-up. Spine, 2009, 34, 20, 2165-70. https://dx.doi.org/10.1097/BRS.0b013e3181b3ff0c.
#480	Crandall 2009	Crandall, Dennis G; Revella, Jan. Transforaminal lumbar interbody fusion versus anterior lumbar interbody fusion as an adjunct to posterior instrumented correction of degenerative lumbar scoliosis: three year clinical and radiographic outcomes. Spine, 2009, 34, 20, 2126-33. https://dx.doi.org/10.1097/BRS.0b013e3181b612db.
#481	Bridwell 2009	Bridwell, Keith H; Glassman, Steven; Horton, William; Shaffrey, Christopher; Schwab, Frank; Zebala, Lukas P; Lenke, Lawrence G; Hilton, Joan F; Shainline, Michael; Baldus, Christine; Wootten, David. Does treatment (nonoperative and operative) improve the two-year quality of life in patients with adult symptomatic lumbar scoliosis: a prospective multicenter evidence-based medicine study. Spine, 2009, 34, 20, 99195. https://dx.doi.org/10.1097/BRS.0b013e3181a8fdc8.
#482	Bess 2009	Bess, Shay; Boachie-Adjei, Oheneba; Burton, Doug; Cunningham, Matthew; Shaffrey, Chris; Shelokov, Alexis; Hostin, Richard; Schwab, Frank; Wood, Kirkham; Akbarnia, Behrooz; International Spine Study Group. Pain and disability determine treatment modality for older patients with adult scoliosis, while deformity guides treatment for younger patients. Spine, 2009, 34, 20, 2186-90. https://dx.doi.org/10.1097/BRS.0b013e3181b05146.
#486	Smith 2009	Smith, Justin S; Shaffrey, Christopher I; Berven, Sigurd; Glassman, Steven; Hamill, Christopher; Horton, William; Ondra, Stephen; Schwab, Frank; Shainline, Michael; Fu, Kai-Ming G; Bridwell, Keith; Spinal Deformity Study Group. Operative versus nonoperative treatment of leg pain in adults with scoliosis: a retrospective review of a prospective multicenter database with two-year follow-up. Spine, 2009, 34, 16, 1693-8. https://dx.doi.org/10.1097/BRS.0b013e3181ac5fcd.
#506	Gorzkowicz 2009	Gorzkowicz, Bozena; Kolban, Maciej; Szych, Zbigniew. Assessment of quality of life in patients with idiopathic scoliosis treated operatively. Ortopedia, traumatologia, rehabilitacja, 2009, 11, 6, 530-41. .

#515	Fu 2010	Fu, Kai-Ming G; Smith, Justin S; Sansur, Charles A; Shaffrey, Christopher I. Standardized measures of health status and disability and the decision to pursue operative treatment in elderly patients with degenerative scoliosis. Neurosurgery, 2010, 66, 1, 42-47. https://dx.doi.org/10.1227/01.NEU.0000361999.29279.E6.
#518	Danielsson 2010	Danielsson, Aina J; Hasserius, Ralph; Ohlin, Acke; Nachemson, Alf L. Health-related quality of life in untreated versus brace-treated patients with adolescent idiopathic scoliosis: a long-term follow-up. Spine, 2010, 35, 2, 199-205. https://dx.doi.org/10.1097/BRS.0b013e3181c89f4a.
#541	Glassman 2010	Glassman, Steven D; Carreon, Leah Y; Shaffrey, Christopher I; Polly, David W; Ondra, Stephen L; Berven, Sigurd H; Bridwell, Keith H. The costs and benefits of nonoperative management for adult scoliosis. Spine, 2010, 35, 5, 578-82. https://dx.doi.org/10.1097/BRS.0b013e3181b0f2f8.
#545	Parent 2010	Parent, Eric C; Dang, Rohan; Hill, Doug; Mahood, Jim; Moreau, Marc; Raso, Jim; Lou, Edmond. Score distribution of the scoliosis research society-22 questionnaire in subgroups of patients of all ages with idiopathic scoliosis. Spine, 2010, 35, 5, 568-77. https://dx.doi.org/10.1097/BRS.0b013e3181b9c9c0.
#575	Carreon 2010	Carreon, Leah Y; Sanders, James O; Diab, Mohammad; Sucato, Daniel J; Sturm, Peter F; Glassman, Steven D; Spinal Deformity Study Group. The minimum clinically important difference in Scoliosis Research Society-22 Appearance, Activity, And Pain domains after surgical correction of adolescent idiopathic scoliosis. Spine, 2010, 35, 23, 2079-83. https://dx.doi.org/10.1097/BRS.0b013e3181c61fd7.
#579	Lin 2010	Lin, Jiu-jenq; Chen, Wei-Hsiu; Chen, Po-Quang; Tsauo, Jau-Yih. Alteration in shoulder kinematics and associated muscle activity in people with idiopathic scoliosis. Spine, 2010, 35, 11, 1151-7. https://dx.doi.org/10.1097/BRS.0b013e3181cd5923.
#586	Negrini 2010	Negrini, Stefano; Minozzi, Silvia; Bettany-Saltikov, Josette; Zaina, Fabio; Chockalingam, Nachiappan; Grivas, Theodoros B; Kotwicki, Tomasz; Maruyama, Toru; Romano, Michele; Vasiliadis, Elias S. Braces for idiopathic scoliosis in adolescents. The Cochrane database of systematic reviews, 2010, , 1, CD006850. https://dx.doi.org/10.1002/14651858.CD006850.pub2.
#589	Keorochana 2010	Keorochana, Gun; Tawonsawatruk, Tulyapruek; Laohachareonsombat, Wichien; Wajanavisit, Wiwat; Jaovisidha, Suphaneewan. The results of decompression and instrumented fusion with pedicular screw plate system in degenerative lumbar scoliosis patients with spinal stenosis: a prospective observational study. Journal of the Medical Association of Thailand = Chotmaihet thangphaet, 2010, 93, 4, 457-61. .

#612	Lubicky 2011	Lubicky, John P; Hanson, Jean E; Riley, Elizabeth H; Spinal Deformity Study Group. Instrumentation constructs in pediatric patients undergoing deformity correction correlated with Scoliosis Research Society scores. Spine, 2011, 36, 20, 1692-700. https://dx.doi.org/10.1097/BRS.0b013e3182102c6a.
#624	Misterska 2011	Misterska, Ewa; Glowacki, Maciej; Ignys-O'Byrne, Anna; Latuszewska, Joanna; Lewandowski, Jacek; Ignys, Iwona; Krauss, Hanna; Piatek, Jacek. Differences in deformity and bracing-related stress between rural and urban area patients with adolescent idiopathic scoliosis treated with a Cheneau brace. Annals of agricultural and environmental medicine : AAEM, 2011, 18, 2, 410-4. .
#644	Bago 2012	Bago, Joan; Perez-Grueso, Francisco Javier Sanchez; Pellise, Ferran; Les, Esther. How do idiopathic scoliosis patients who improve after surgery differ from those who do not exceed a minimum detectable change?. European spine journal : official publication of the European Spine Society, the European Spinal Deformity Society, and the European Section of the Cervical Spine Research Society, 2012, 21, 1, 18415. https://dx.doi.org/10.1007/s00586-011-2017-x.
#649	O'Shaughnessy 2012	O'Shaughnessy, Brian A; Bridwell, Keith H; Lenke, Lawrence G; Cho, Woojin; Baldus, Christine; Chang, Michael S; Auerbach, Joshua D; Crawford, Charles H. Does a long-fusion "T3-sacrum" portend a worse outcome than a short-fusion "T10-sacrum" in primary surgery for adult scoliosis?. Spine, 2012, 37, 10, 884-90. https://dx.doi.org/10.1097/BRS.0b013e3182376414.
#653	Danielsson 2012	Danielsson, Aina J; Hasserius, Ralph; Ohlin, Acke; Nachemson, Alf L. Body appearance and quality of life in adult patients with adolescent idiopathic scoliosis treated with a brace or under observation alone during adolescence. Spine, 2012, 37, 9, 755-62. https://dx.doi.org/10.1097/BRS.0b013e318231493c.
#660	Morse 2012	Morse, Lee Jae; Kawakami, Noriaki; Lenke, Lawrence G; Sucato, Daniel J; Sanders, James O; Diab, Mohammad. Culture and ethnicity influence outcomes of the Scoliosis Research Society Instrument in adolescent idiopathic scoliosis. Spine, 2012, 37, 12, 1072-6. https://dx.doi.org/10.1097/BRS.0b013e31823ed962.
#678	Min 2012	Min, Kan; Haefeli, Mathias; Mueller, Daniel; Klammer, Georg; Hahn, Frederik. Anterior short correction in thoracic adolescent idiopathic scoliosis with mini-open thoracotomy approach: prospective clinical, radiological and pulmonary function results. European spine journal : official publication of the European Spine Society, the European Spinal Deformity Society, and the European Section of the Cervical Spine Research Society, 2012, 21 Suppl 6, 9301980, b9y, S765-72. https://dx.doi.org/10.1007/s00586-012-2156-8.
#679	Zeng 2012	Zeng, Yan; White, Andrew P; Albert, Todd J; Chen, Zhongqiang. Surgical strategy in adult lumbar scoliosis: the utility of categorization into 2 groups based on primary symptom, each with 2-year minimum follow-up. Spine, 2012, 37, 9, E556-61. https://dx.doi.org/10.1097/BRS.0b013e31824af5c6.

#681	Deceuninck 2012	Deceuninck, J; Bernard, J C. Quality of life and brace-treated idiopathic scoliosis: a cross-sectional study performed at the Centre des Massues on a population of 120 children and adolescents. Annals of physical and rehabilitation medicine, 2012, 55, 2, 93-102. https://dx.doi.org/10.1016/j.rehab.2011.12.003.
#683	Misterska 2012	Misterska, Ewa; Glowacki, Maciej; Latuszewska, Joanna. Female patients' and parents' assessment of deformity- and brace-related stress in the conservative treatment of adolescent idiopathic scoliosis. Spine, 2012, 37, 14, 1218-23. https://dx.doi.org/10.1097/BRS.0b013e31824b66d4.
#685	Rothenfluh 2012	Rothenfluh, Dominique A; Neubauer, Georg; Klasen, Juergen; Min, Kan. Analysis of internal construct validity of the SRS-24 questionnaire. European spine journal : official publication of the European Spine Society, the European Spinal Deformity Society, and the European Section of the Cervical Spine Research Society, 2012, 21, 8, 1590-5. .
#702	Glassman 2007	Glassman, Steven D; Schwab, Frank J; Bridwell, Keith H; Ondra, Stephen L; Berven, Sigurd; Lenke, Lawrence G. The selection of operative versus nonoperative treatment in patients with adult scoliosis. Spine, 2007, 32, 1, 34151. .
#741	Parent 2007	Parent, Eric C; Hill, Doug; Moreau, Marc; Mahood, Jim; Raso, Jim; Lou, Edmond. Score distribution of the Scoliosis Quality of Life Index questionnaire in different subgroups of patients with adolescent idiopathic scoliosis. Spine, 2007, 32, 16, 1767-77. .
#742	Glattes 2007	Glattes, R Christopher; Burton, Douglas C; Lai, Sue Min; Frasier, Elizabeth; Asher, Marc A. The reliability and concurrent validity of the Scoliosis Research Society-22r patient questionnaire compared with the Child Health Questionnaire-CF87 patient questionnaire for adolescent spinal deformity. Spine, 2007, 32, 16, 1778-84. .
#763	Watanabe 2007	Watanabe, Kei; Lenke, Lawrence G; Bridwell, Keith H; Hasegawa, Kazuhiro; Hirano, Toru; Endo, Naoto; Cheh, Gene; Kim, Yongjung J; Hensley, Marsha; Stobbs, Georgia; Koester, Linda. Cross-cultural comparison of the Scoliosis Research Society Outcomes Instrument between American and Japanese idiopathic scoliosis patients: are there differences?. Spine, 2007, 32, 24, 296303. .
#764	Howard 2007	Howard, Andrew; Donaldson, Sandra; Hedden, Douglas; Stephens, Derek; Alman, Benjamin; Wright, James. Improvement in quality of life following surgery for adolescent idiopathic scoliosis. Spine, 2007, 32, 24, 297886. .

#765	Sanders 2007	Sanders, James O; Harrast, John J; Kuklo, Timothy R; Polly, David W; Bridwell, Keith H; Diab, Mohammad; Dormans, John P; Drummond, Denis S; Emans, John B; Johnston, Charles E 2nd; Lenke, Lawrence G; McCarthy, Richard E; Newton, Peter O; Richards, B Stephens; Sucato, Daniel J; Spinal Deformity Study Group. The Spinal Appearance Questionnaire: results of reliability, validity, and responsiveness testing in patients with idiopathic scoliosis. Spine, 2007, 32, 24, 2719-22. .
#766	Schwab 2007	Schwab, Frank; Lafage, Virginie; Farcy, Jean-Pierre; Bridwell, Keith; Glassman, Steven; Ondra, Stephen; Lowe, Tom; Shainline, Michael. Surgical rates and operative outcome analysis in thoracolumbar and lumbar major adult scoliosis: application of the new adult deformity classification. Spine, 2007, 32, 24, 2723-30. .
#799	Peelle 2008	Peelle, Michael W; Boachie-Adjei, Oheneba; Charles, Gina; Kanazawa, Yamuna; Mesfin, Addisu. Lumbar curve response to selective thoracic fusion in adult idiopathic scoliosis. The spine journal : official journal of the North American Spine Society, 2008, 8, 6, 897-903. https://dx.doi.org/10.1016/j.spinee.2007.11.010.
#808	Morton 2008	Morton, Anne; Riddle, Russ; Buchanan, Renee; Katz, Don; Birch, John. Accuracy in the prediction and estimation of adherence to bracewear before and during treatment of adolescent idiopathic scoliosis. Journal of pediatric orthopedics, 2008, 28, 3, 336-41. https://dx.doi.org/10.1097/BPO.0b013e318168d154.
#809	Mulcahey 2008	Mulcahey, M J; Haley, Stephen M; Duffy, Theresa; Pengsheng, Ni; Betz, Randal R. Measuring physical functioning in children with spinal impairments with computerized adaptive testing. Journal of pediatric orthopedics, 2008, 28, 3, 330-5. https://dx.doi.org/10.1097/BPO.0b013e318168c792.
#820	Vasiliadis 2008	Vasiliadis, Elias; Grivas, Theodoros B. Quality of life after conservative treatment of adolescent idiopathic scoliosis. Studies in health technology and informatics, 2008, 135, ck1, 9214582, 409-13. .
#832	Upasani 2008	Upasani, Vidyadhar V; Caltoum, Christine; Petcharaporn, Maty; Bastrom, Tracey P; Pawelek, Jeff B; Betz, Randal R; Clements, David H; Lenke, Lawrence G; Lowe, Thomas G; Newton, Peter O. Adolescent idiopathic scoliosis patients report increased pain at five years compared with two years after surgical treatment. Spine, 2008, 33, 10, 1107-12. https://dx.doi.org/10.1097/BRS.0b013e31816f2849.
#862	Baldus 2008	Baldus, Christine; Bridwell, Keith H; Harrast, John; Edwards, Charles 2nd; Glassman, Steven; Horton, William; Lenke, Lawrence G; Lowe, Thomas; Mardjetko, Steve; Ondra, Stephen; Schwab, Frank; Shaffrey, Christopher. Age-gender matched comparison of SRS instrument scores between adult deformity and normal adults: are all SRS domains disease specific?. Spine, 2008, 33, 20, 114900. https://dx.doi.org/10.1097/BRS.0b013e31817c0466.

#871	Hill 2008	Hill, D; Parent, E; Lou, E; Mahood, J. Can future back pain in AIS subjects be predicted during adolescence from the severity of the deformity?. Studies in health technology and informatics, 2008, 140, ck1, 9214582, 249-53. .
#874	Zeh 2008	Zeh, Alexander; Planert, Michael; Klima, Stefan; Hein, Werner; Wohlrab, David. The flexible Triac-Brace for conservative treatment of idiopathic scoliosis. An alternative treatment option?. Acta orthopaedica Belgica, 2008, 74, 4, 512-21. .
#878	Newton 2008	Newton, Peter O; Upasani, Vidyadhar V; Lhamby, Juliano; Ugrinow, Valerie L; Pawelek, Jeff B; Bastrom, Tracey P. Surgical treatment of main thoracic scoliosis with thoracoscopic anterior instrumentation. a five-year follow-up study. The Journal of bone and joint surgery. American volume, 2008, 90, 10, 2077-89. https://dx.doi.org/10.2106/JBJS.G.01315.
#883	Smith 2008	Smith, Justin S; Fu, Kai-Ming; Urban, Peter; Shaffrey, Christopher I. Neurological symptoms and deficits in adults with scoliosis who present to a surgical clinic: incidence and association with the choice of operative versus nonoperative management. Journal of neurosurgery. Spine, 2008, 9, 4, 326-31. https://dx.doi.org/10.3171/SPI.2008.9.10.326.
#919	Blondel 2012	Blondel, Benjamin; Schwab, Frank; Ungar, Benjamin; Smith, Justin; Bridwell, Keith; Glassman, Steven; Shaffrey, Christopher; Farcy, Jean-Pierre; Lafage, Virginie. Impact of magnitude and percentage of global sagittal plane correction on health-related quality of life at 2-years follow-up. Neurosurgery, 2012, 71, 2, 341-348. https://dx.doi.org/10.1227/NEU.0b013e31825d20c0.
#939	Kinel 2012	Kinel, Edyta; Kotwicki, Tomasz; Podolska, Anna; Bialek, Marianna; Stryla, Wanda. Quality of life and stress level in adolescents with idiopathic scoliosis subjected to conservative treatment. Studies in health technology and informatics, 2012, 176, ck1, 9214582, 419-22. .
#941	Tomaszewski 2012	Tomaszewski, Ryszard; Janowska, Magdalena. Psychological aspects of scoliosis surgery in children. Studies in health technology and informatics, 2012, 176, ck1, 9214582, 428-32. .
#948	Diab 2012	Diab, Aliaa A. The role of forward head correction in management of adolescent idiopathic scoliotic patients: a randomized controlled trial. Clinical rehabilitation, 2012, 26, 12, 1123-32. https://dx.doi.org/10.1177/0269215512447085.

#956	Leszczewska 2012	Leszczewska, Justyna; Czaprowski, Dariusz; Pawlowska, Paulina; Kolwicz, Aleksandra; Kotwicki, Tomasz. Evaluation of the stress level of children with idiopathic scoliosis in relation to the method of treatment and parameters of the deformity. TheScientificWorldJournal, 2012, 2012, 101131163, 538409. https://dx.doi.org/10.1100/2012/538409.
#988	Newton 2013	Newton, Peter O; Marks, Michelle C; Bastrom, Tracey P; Betz, Randal; Clements, David; Lonner, Baron; Crawford, Alvin; Shufflebarger, Harry; O'Brien, Michael; Yaszay, Burt; Harms Study Group. Surgical treatment of Lenke 1 main thoracic idiopathic scoliosis: results of a prospective, multicenter study. Spine, 2013, 38, 4, 328-38. https://dx.doi.org/10.1097/BRS.0b013e31826c6df4.
#990	Bharucha 2013	Bharucha, Neil J; Lonner, Baron S; Auerbach, Joshua D; Kean, Kristin E; Trobisch, Per D. Low-density versus high-density thoracic pedicle screw constructs in adolescent idiopathic scoliosis: do more screws lead to a better outcome?. The spine journal : official journal of the North American Spine Society, 2013, 13, 4, 375-81. https://dx.doi.org/10.1016/j.spinee.2012.05.029.
#993	Lerner 2013	Lerner, Thomas; Liljenqvist, Ulf. Silicate-substituted calcium phosphate as a bone graft substitute in surgery for adolescent idiopathic scoliosis. European spine journal : official publication of the European Spine Society, the European Spinal Deformity Society, and the European Section of the Cervical Spine Research Society, 2013, 22 Suppl 2, 9301980, b9y, S185-94. https://dx.doi.org/10.1007/s00586-012-2485-7.
#998	Smith 2013	Smith, Justin S; Shaffrey, Christopher I; Glassman, Steven D; Carreon, Leah Y; Schwab, Frank J; Lafage, Virginie; Arlet, Vincent; Fu, Kai-Ming G; Bridwell, Keith H; Spinal Deformity Study Group. Clinical and radiographic parameters that distinguish between the best and worst outcomes of scoliosis surgery for adults. European spine journal : official publication of the European Spine Society, the European Spinal Deformity Society, and the European Section of the Cervical Spine Research Society, 2013, 22, 2, 402-10. https://dx.doi.org/10.1007/s00586-012-2547-x.
#1001	Wang 2013	Wang, Michael Y. Improvement of sagittal balance and lumbar lordosis following less invasive adult spinal deformity surgery with expandable cages and percutaneous instrumentation. Journal of neurosurgery. Spine, 2013, 18, 1, 44663. https://dx.doi.org/10.3171/2012.9.SPINE111081.
#1004	Park 2013	Park, Paul; La Marca, Frank. Combined "hybrid" open and minimally invasive surgical correction of adult thoracolumbar scoliosis: a retrospective cohort study. Neurosurgery, 2013, 72, 2, 151-9. https://dx.doi.org/10.1227/NEU.0b013e31827b9d55.
#1007	Misterska 2013	Misterska, Ewa; Glowacki, Maciej; Latuszewska, Joanna; Adamczyk, Katarzyna. Perception of stress level, trunk appearance, body function and mental health in females with adolescent idiopathic scoliosis treated conservatively: a longitudinal analysis. Quality of life research : an international journal of quality of life aspects of treatment, care and rehabilitation, 2013, 22, 7, 1633-45. https://dx.doi.org/10.1007/s11136-012-0316-2.

#1029	Schwab 2013	Schwab, Frank J; Blondel, Benjamin; Bess, Shay; Hostin, Richard; Shaffrey, Christopher I; Smith, Justin S; Boachie-Adjei, Oheneba; Burton, Douglas C; Akbarnia, Behrooz A; Mundis, Gregory M; Ames, Christopher P; Kebaish, Khaled; Hart, Robert A; Farcy, Jean-Pierre; Lafage, Virginie; International Spine Study Group (ISSG). Radiographical spinopelvic parameters and disability in the setting of adult spinal deformity: a prospective multicenter analysis. Spine, 2013, 38, 13, E803-12. https://dx.doi.org/10.1097/BRS.0b013e318292b7b9.
#1034	Smith 2013	Smith, Justin S; Klineberg, Eric; Schwab, Frank; Shaffrey, Christopher I; Moal, Bertrand; Ames, Christopher P; Hostin, Richard; Fu, Kai-Ming G; Burton, Douglas; Akbarnia, Behrooz; Gupta, Munish; Hart, Robert; Bess, Shay; Lafage, Virginie; International Spine Study Group. Change in classification grade by the SRS-Schwab Adult Spinal Deformity Classification predicts impact on health-related quality of life measures: prospective analysis of operative and nonoperative treatment. Spine, 2013, 38, 19, 1663-71. https://dx.doi.org/10.1097/BRS.0b013e31829ec563.
#1036	Zebracki 2013	Zebracki, Kathy; Thawrani, Dinesh; Oswald, Timothy S; Anadio, Jennifer M; Sturm, Peter F; Spine Deformity Study Group. Predictors of emotional functioning in youth after surgical correction of idiopathic scoliosis. Journal of pediatric orthopedics, 2013, 33, 6, 624-7. https://dx.doi.org/10.1097/BPO.0b013e318288b77f.
#1046	Danielsson 2013	Danielsson, Aina J; Romberg, Karin. Reliability and validity of the Swedish version of the Scoliosis Research Society-22 (SRS-22r) patient questionnaire for idiopathic scoliosis. Spine, 2013, 38, 21, 1875-84. https://dx.doi.org/10.1097/BRS.0b013e3182a211c0.
#1050	Phillips 2013	Phillips, Frank M; Isaacs, Robert E; Rodgers, William Blake; Khajavi, Kaveh; Tohmeh, Antoine G; Deviren, Vedat; Peterson, Mark D; Hyde, Jonathan; Kurd, Mark. Adult degenerative scoliosis treated with XLIF: clinical and radiographical results of a prospective multicenter study with 24-month follow-up. Spine, 2013, 38, 21, 1853-61. https://dx.doi.org/10.1097/BRS.0b013e3182a43f0b.
#1052	Bastrom 2013	Bastrom, Tracey P; Marks, Michelle C; Yaszay, Burt; Newton, Peter O; Harms Study Group. Prevalence of postoperative pain in adolescent idiopathic scoliosis and the association with preoperative pain. Spine, 2013, 38, 21, 1848-52. https://dx.doi.org/10.1097/BRS.0b013e3182a4aa97.
#1053	Deukmedjian 2013	Deukmedjian, Armen R; Ahmadian, Amir; Bach, Konrad; Zouzias, Alexandros; Uribe, Juan S. Minimally invasive lateral approach for adult degenerative scoliosis: lessons learned. Neurosurgical focus, 2013, 35, 2, E4. https://dx.doi.org/10.3171/2013.5.FOCUS13173.
#1067	Weinstein 2013	Weinstein, Stuart L; Dolan, Lori A; Wright, James G; Dobbs, Matthew B. Effects of bracing in adolescents with idiopathic scoliosis. The New England journal of medicine, 2013, 369, 16, 1512-21. https://dx.doi.org/10.1056/NEJMoa1307337.

#1080	Ersberg 2013	Ersberg, Anna; Gerdhem, Paul. Pre- and postoperative quality of life in patients treated for scoliosis. Acta orthopaedica, 2013, 84, 6, 537-43. https://dx.doi.org/10.3109/17453674.2013.854667.
#1085	Filippiadis 2013	Filippiadis, Dimitrios K; Papagelopoulos, Panagiotis; Kitsou, Maria; Oikonomopoulos, Nikolaos; Brountzos, Elias; Kelekis, Nikolaos; Kelekis, Alexis. Percutaneous vertebroplasty in adult degenerative scoliosis for spine support: study for pain evaluation and mobility improvement. BioMed research international, 2013, 2013, 101600173, 626502. https://dx.doi.org/10.1155/2013/626502.
#1088	Sieberg 2013	Sieberg, Christine B; Simons, Laura E; Edelstein, Mark R; DeAngelis, Maria R; Pielech, Melissa; Sethna, Navil; Hresko, M Timothy. Pain prevalence and trajectories following pediatric spinal fusion surgery. The journal of pain : official journal of the American Pain Society, 2013, 14, 12, 1694-702. https://dx.doi.org/10.1016/j.jpain.2013.09.005.
#1093	Pellegrino 2014	Pellegrino, Luciano N; Avanzi, Osmar. Prospective evaluation of quality of life in adolescent idiopathic scoliosis before and after surgery. Journal of spinal disorders & techniques, 2014, 27, 8, 409-14. https://dx.doi.org/10.1097/BSD.0b013e3182797a5e.
#1101	Garabekyan 2014	Garabekyan, Tigran; Hosseinzadeh, Pooya; Iwinski, Henry J; Muchow, Ryan D; Talwalkar, Vishwas R; Walker, Janet; Milbrandt, Todd A. The results of preoperative halo-gravity traction in children with severe spinal deformity. Journal of pediatric orthopedics. Part B, 2014, 23, 1, 44566. https://dx.doi.org/10.1097/BPB.0b013e32836486b6.
#1103	Cheung 2007	Cheung, Kenneth M C; Cheng, Elaine Y L; Chan, Samantha C W; Yeung, Kelvin W K; Luk, Keith D K. Outcome assessment of bracing in adolescent idiopathic scoliosis by the use of the SRS-22 questionnaire. International orthopaedics, 2007, 31, 4, 507-11. .
#1111	Andersen 2010	Andersen, Mikkel O; Thomsen, Karsten; Kyvik, Kirsten O. Perceived health status in self-reported adolescent idiopathic scoliosis: a survey based on a population of twins. Spine, 2010, 35, 16, 1571-4. https://dx.doi.org/10.1097/BRS.0b013e3181e47dab.
#1119	Sanders 2010	Sanders, James O; Carreon, Leah Y; Sucato, Daniel J; Sturm, Peter F; Diab, Mohammad; Spinal Deformity Study Group. Preoperative and perioperative factors effect on adolescent idiopathic scoliosis surgical outcomes. Spine, 2010, 35, 20, 1867-71. https://dx.doi.org/10.1097/BRS.0b013e3181efa6f5.

#1120	Bridwell 2010	Bridwell, Keith H; Baldus, Christine; Berven, Sigurd; Edwards, Charles 2nd; Glassman, Steven; Hamill, Christopher; Horton, William; Lenke, Lawrence G; Ondra, Stephen; Schwab, Frank; Shaffrey, Christopher; Wootten, David. Changes in radiographic and clinical outcomes with primary treatment adult spinal deformity surgeries from two years to three- to five-years follow-up. Spine, 2010, 35, 20, 1849-54. https://dx.doi.org/10.1097/BRS.0b013e3181efa06a.
#1121	Zimmerman 2010	Zimmerman, Ryan M; Mohamed, Ahmed S; Skolasky, Richard L; Robinson, Malaya D; Kebaish, Khaled M. Functional outcomes and complications after primary spinal surgery for scoliosis in adults aged forty years or older: a prospective study with minimum two-year follow-up. Spine, 2010, 35, 20, 1861-6. https://dx.doi.org/10.1097/BRS.0b013e3181e57827.
#1122	Crawford 2010	Crawford, Charles H 3rd; Bridwell, Keith H; Cho, Woojin; Buchowski, Jacob M; O'Shaughnessy, Brian A; Chang, Michael S; Auerbach, Josh D. Extension of prior idiopathic scoliosis fusions to the sacrum: a matched cohort analysis of sixty patients with minimum two-year follow-up. Spine, 2010, 35, 20, 1843-8. https://dx.doi.org/10.1097/BRS.0b013e3181e03115.
#1123	Transfeldt 2010	Transfeldt, Ensor E; Topp, Raymond; Mehbod, Amir A; Winter, Robert B. Surgical outcomes of decompression, decompression with limited fusion, and decompression with full curve fusion for degenerative scoliosis with radiculopathy. Spine, 2010, 35, 20, 1872-5. https://dx.doi.org/10.1097/BRS.0b013e3181ce63a2.
#1138	Monticone 2010	Monticone, Marco; Baiardi, Paola; Calabro, David; Calabro, Fabio; Foti, Calogero. Development of the Italian version of the revised Scoliosis Research Society-22 Patient Questionnaire, SRS-22r-I: cross-cultural adaptation, factor analysis, reliability, and validity. Spine, 2010, 35, 24, E1412-7. https://dx.doi.org/10.1097/BRS.0b013e3181e88981.
#1153	Misterska 2010	Misterska, Ewa; Glowacki, Maciej; Harasymczuk, Jerzy. Personality characteristics of females with adolescent idiopathic scoliosis after brace or surgical treatment compared to healthy controls. Medical science monitor : international medical journal of experimental and clinical research, 2010, 16, 12, CR606-15. .
#1164	Roy-Beaudry 2011	Roy-Beaudry, Marjolaine; Beausejour, Marie; Joncas, Julie; Forcier, Martin; Bekhiche, Sara; Labelle, Hubert; Grimard, Guy; Parent, Stefan. Validation and clinical relevance of a French-Canadian version of the spinal appearance questionnaire in adolescent patients. Spine, 2011, 36, 9, 746-51. https://dx.doi.org/10.1097/BRS.0b013e3181e040e7.
#1166	Smith 2011	Smith, Justin S; Shaffrey, Christopher I; Glassman, Steven D; Berven, Sigurd H; Schwab, Frank J; Hamill, Christopher L; Horton, William C; Ondra, Stephen L; Sansur, Charles A; Bridwell, Keith H; Spinal Deformity Study Group. Risk-benefit assessment of surgery for adult scoliosis: an analysis based on patient age. Spine, 2011, 36, 10, 817-24. https://dx.doi.org/10.1097/BRS.0b013e3181e21783.

#1174	Li 2011	Li, Fangcai; Chen, Qixin; Chen, Weishan; Xu, Kan; Wu, Qionghua. Posterior-only approach with selective segmental TLIF for degenerative lumbar scoliosis. Journal of spinal disorders & techniques, 2011, 24, 5, 308-12. https://dx.doi.org/10.1097/BSD.0b013e3181f9a7d5.
#1181	Tsai 2011	Tsai, Tai-Hsin; Huang, Tzuu-Yuan; Lieu, Ann-Shung; Lee, Kung-Shing; Kung, Sui-Sum; Chu, Cheng-Wei; Hwang, Shiuh-Lin. Functional outcome analysis: instrumented posterior lumbar interbody fusion for degenerative lumbar scoliosis. Acta neurochirurgica, 2011, 153, 3, 547-55. https://dx.doi.org/10.1007/s00701-010-0909-x.
#1194	Zhang 2011	Zhang, Jingtao; He, Dawei; Gao, Juan; Yu, Xiuchun; Sun, Haining; Chen, Ziqiang; Li, Ming. Changes in life satisfaction and self-esteem in patients with adolescent idiopathic scoliosis with and without surgical intervention. Spine, 2011, 36, 9, 741-5. https://dx.doi.org/10.1097/BRS.0b013e3181e0f034.
#1197	Carreon 2011	Carreon, Leah Yacat; Sanders, James O; Diab, Mohammad; Sturm, Peter F; Sucato, Daniel J; Spinal Deformity Study Group. Patient satisfaction after surgical correction of adolescent idiopathic scoliosis. Spine, 2011, 36, 12, 965-8. https://dx.doi.org/10.1097/BRS.0b013e3181e92b1d.
#1201	Smucny 2011	Smucny, Mia; Lubicky, John P; Sanders, James O; Carreon, Leah Y; Diab, Mohammad. Patient self-assessment of appearance is improved more by all pedicle screw than by hybrid constructs in surgical treatment of adolescent idiopathic scoliosis. Spine, 2011, 36, 3, 248-54. https://dx.doi.org/10.1097/BRS.0b013e3181cdb4be.
#1218	Hong 2011	Hong, Jae-Young; Suh, Seung-Woo; Easwar, T R; Modi, Hitesh N; Yang, Jae-Hyuk; Park, Jung-Ho. Evaluation of the three-dimensional deformities in scoliosis surgery with computed tomography: efficacy and relationship with clinical outcomes. Spine, 2011, 36, 19, E1259-65. https://dx.doi.org/10.1097/BRS.0b013e318205e413.
#1221	Carreon 2011	Carreon, Leah Y; Sanders, James O; Polly, David W; Sucato, Daniel J; Parent, Stefan; Roy-Beaudry, Marjolaine; Hopkins, Jeffrey; McClung, Anna; Bratcher, Kelly R; Diamond, Beverly E; Spinal Deformity Study Group. Spinal appearance questionnaire: factor analysis, scoring, reliability, and validity testing. Spine, 2011, 36, 18, E1240-4. https://dx.doi.org/10.1097/BRS.0b013e318204f987.
#1235	Urrutia 2011	Urrutia, Julio; Espinosa, Julio; Diaz-Ledezma, Claudio; Cabello, Carlos. The impact of lumbar scoliosis on pain, function and health-related quality of life in postmenopausal women. European spine journal : official publication of the European Spine Society, the European Spinal Deformity Society, and the European Section of the Cervical Spine Research Society, 2011, 20, 12, 118156. https://dx.doi.org/10.1007/s00586-011-1829-z.

#1243	Fu 2011	Fu, Kai-Ming G; Rhagavan, Prashant; Shaffrey, Christopher I; Chernavvsky, Daniel R; Smith, Justin S. Prevalence, severity, and impact of foraminal and canal stenosis among adults with degenerative scoliosis. Neurosurgery, 2011, 69, 6, 1181-7. https://dx.doi.org/10.1227/NEU.0b013e31822a9aeb.
#1256	Lonjon 2014	Lonjon, Guillaume; Ilharreborde, Brice; Odent, Thierry; Moreau, Sebastien; Glorion, Christophe; Mazda, Keyvan. Reliability and validity of the French-Canadian version of the scoliosis research society 22 questionnaire in France. Spine, 2014, 39, 1, E26-34. https://dx.doi.org/10.1097/BRS.0000000000000080.
#1270	Brox 2014	Brox, J I; Lange, J E; Steen, H. Comorbidity influenced health-related quality of life of 390 patients with idiopathic scoliosis at long-term follow-up. European journal of physical and rehabilitation medicine, 2014, 50, 1, 73-81. .
#1278	Schlosser 2014	Schlosser, Tom P C; Stadhouder, Agnita; Schimmel, Janneke J P; Lehr, A Mechteld; van der Heijden, Geert J M G; Castelein, Rene M. Reliability and validity of the adapted Dutch version of the revised Scoliosis Research Society 22-item questionnaire. The spine journal : official journal of the North American Spine Society, 2014, 14, 8, 1663-72. https://dx.doi.org/10.1016/j.spinee.2013.09.046.
#1280	Noh 2014	Noh, Dong Koog; You, Joshua Sung-H; Koh, Jae-Hyun; Kim, Hoseong; Kim, Donghyun; Ko, Sung-Mok; Shin, Ji-Youn. Effects of novel corrective spinal technique on adolescent idiopathic scoliosis as assessed by radiographic imaging. Journal of back and musculoskeletal rehabilitation, 2014, 27, 3, 331-8. https://dx.doi.org/10.3233/BMR-130452.
#1282	Scheer 2014	Scheer, Justin K; Lafage, Virginie; Smith, Justin S; Deviren, Vedat; Hostin, Richard; McCarthy, Ian M; Mundis, Gregory M; Burton, Douglas C; Klineberg, Eric; Gupta, Munish C; Kebaish, Khaled M; Shaffrey, Christopher I; Bess, Shay; Schwab, Frank; Ames, Christopher P; International Spine Study Group. Impact of age on the likelihood of reaching a minimum clinically important difference in 374 three-column spinal osteotomies: clinical article. Journal of neurosurgery. Spine, 2014, 20, 3, 306-12. https://dx.doi.org/10.3171/2013.12.SPINE13680.
#1298	Ding 2014	Ding, Ran; Liang, Jinqian; Qiu, Guixing; Shen, Jianxiong; Li, Zheng. Evaluation of quality of life in adolescent idiopathic scoliosis with different distal fusion level: a comparison of L3 versus L4. Journal of spinal disorders & techniques, 2014, 27, 5, E155-61. https://dx.doi.org/10.1097/BSD.0000000000000073.
#1299	Caronni 2014	Caronni, Antonio; Zaina, Fabio; Negrini, Stefano. Improving the measurement of health-related quality of life in adolescent with idiopathic scoliosis: the SRS-7, a Rasch-developed short form of the SRS-22 questionnaire. Research in developmental disabilities, 2014, 35, 4, 784-99. https://dx.doi.org/10.1016/j.ridd.2014.01.020.

#1309	Fu 2014	Fu, Lingjie; Chang, Michael S; Crandall, Dennis G; Revella, Jan. Comparative analysis of clinical outcomes and complications in patients with degenerative scoliosis undergoing primary versus revision surgery. Spine, 2014, 39, 10, 805-11. https://dx.doi.org/10.1097/BRS.0000000000000283.
#1312	Verma 2014	Verma, Kushagra; Lonner, Baron; Toombs, Courtney S; Ferrise, Paige; Wright, Bettye; King, Akilah B; Boachie-Adjei, Oheneba. International utilization of the SRS-22 instrument to assess outcomes in adolescent idiopathic scoliosis: what can we learn from a medical outreach group in Ghana?. Journal of pediatric orthopedics, 2014, 34, 5, 503-8. https://dx.doi.org/10.1097/BPO.0000000000000137.
#1314	Khajavi 2014	Khajavi, Kaveh; Shen, Alessandria Y. Two-year radiographic and clinical outcomes of a minimally invasive, lateral, transpsoas approach for anterior lumbar interbody fusion in the treatment of adult degenerative scoliosis. European spine journal : official publication of the European Spine Society, the European Spinal Deformity Society, and the European Section of the Cervical Spine Research Society, 2014, 23, 6, 1215-23. https://dx.doi.org/10.1007/s00586-014-3246-6.
#1319	Monticone 2014	Monticone, Marco; Ambrosini, Emilia; Cazzaniga, Daniele; Rocca, Barbara; Ferrante, Simona. Active self-correction and task-oriented exercises reduce spinal deformity and improve quality of life in subjects with mild adolescent idiopathic scoliosis. Results of a randomised controlled trial. European spine journal : official publication of the European Spine Society, the European Spinal Deformity Society, and the European Section of the Cervical Spine Research Society, 2014, 23, 6, 1204-14. https://dx.doi.org/10.1007/s00586-014-3241-y.
#1321	Hoashi 2014	Hoashi, Jane S; Samdani, Amer F; Betz, Randal R; Bastrom, Tracey P; Harms Study Group; Cahill, Patrick J. Is there a "July effect" in surgery for adolescent idiopathic scoliosis?. The Journal of bone and joint surgery. American volume, 2014, 96, 7, e55. https://dx.doi.org/10.2106/JBJS.M.00150.
#1324	Mesfin 2014	Mesfin, Addisu; Lenke, Lawrence G; Bridwell, Keith H; Akhtar, Usman; Jupitz, Jennifer M; Fogelson, Jeremy L; Hershman, Stuart H; Kim, Han J; Koester, Linda A. Does preoperative narcotic use adversely affect outcomes and complications after spinal deformity surgery? A comparison of nonnarcotic- with narcotic-using groups. The spine journal : official journal of the North American Spine Society, 2014, 14, 12, 2819-25. https://dx.doi.org/10.1016/j.spinee.2014.03.049.
#1334	Auerbach 2014	Auerbach, Joshua D; Lonner, Baron S; Crerand, Canice E; Shah, Suken A; Flynn, John M; Bastrom, Tracey; Penn, Phedra; Ahn, Jennifer; Toombs, Courtney; Bharucha, Neil; Bowe, Whitney P; Newton, Peter O. Body image in patients with adolescent idiopathic scoliosis: validation of the Body Image Disturbance Questionnaire--Scoliosis Version. The Journal of bone and joint surgery. American volume, 2014, 96, 8, e61. https://dx.doi.org/10.2106/JBJS.L.00867.
#1337	Tempel 2014	Tempel, Zachary J; Gandhoke, Gurpreet S; Bonfield, Christopher M; Okonkwo, David O; Kanter, Adam S. Radiographic and clinical outcomes following combined lateral lumbar interbody fusion and posterior segmental stabilization in patients with adult degenerative scoliosis. Neurosurgical focus, 2014, 36, 5, E11. https://dx.doi.org/10.3171/2014.3.FOCUS13368.

#1338	Haque 2014	Haque, Raqeeb M; Mundis, Gregory M Jr; Ahmed, Yousef; El Ahmadieh, Tarek Y; Wang, Michael Y; Mummaneni, Praveen V; Uribe, Juan S; Okonkwo, David O; Eastlack, Robert K; Anand, Neel; Kanter, Adam S; La Marca, Frank; Akbarnia, Behrooz A; Park, Paul; Lafage, Virginie; Terran, Jamie S; Shaffrey, Christopher I; Klineberg, Eric; Deviren, Vedat; Fessler, Richard G; International Spine Study Group. Comparison of radiographic results after minimally invasive, hybrid, and open surgery for adult spinal deformity: a multicenter study of 184 patients. Neurosurgical focus, 2014, 36, 5, E13. https://dx.doi.org/10.3171/2014.3.FOCUS1424.
#1349	Fu 2014	Fu, Kai-Ming G; Bess, Shay; Shaffrey, Christopher I; Smith, Justin S; Lafage, Virginie; Schwab, Frank; Burton, Douglas C; Akbarnia, Behrooz A; Ames, Christopher P; Boachie-Adjei, Oheneba; Deverin, Vedat; Hart, Robert A; Hostin, Richard; Klineberg, Eric; Gupta, Munish; Kebaish, Khaled; Mundis, Gregory; Mummaneni, Praveen V; International Spine Study Group. Patients with adult spinal deformity treated operatively report greater baseline pain and disability than patients treated nonoperatively; however, deformities differ between age groups. Spine, 2014, 39, 17, 1401-7. https://dx.doi.org/10.1097/BRS.0000000000000414.
#1350	Urquhart 2014	Urquhart, D S; Gallella, S; Gidaris, D; Brady, E; Blacklock, S; Tsirikos, A I. Six-year follow-up study on the effect of combined anterior and posterior spinal fusion on lung function and quality of life in young people with adolescent idiopathic scoliosis. Archives of disease in childhood, 2014, 99, 10, 922-6. https://dx.doi.org/10.1136/archdischild-2013-305739.
#1361	dosSantosAlves 2014	dos Santos Alves, Vera Lucia; Alves da Silva, Renato Jose Azevedo Leite; Avanzi, Osmar. Effect of a preoperative protocol of aerobic physical therapy on the quality of life of patients with adolescent idiopathic scoliosis: a randomized clinical study. American journal of orthopedics (Belle Mead, N.J.), 2014, 43, 6, E112-6. .
#1376	Hines 2015	Hines, Tabatha; Roland, Sandy; Nguyen, Dylan; Kennard, Beth; Richard, Heather; Hughes, Carroll W; McClintock, Shawn M; Ramo, Brandon; Herring, Tony. School Scoliosis Screenings: Family Experiences and Potential Anxiety After Orthopaedic Referral. Spine, 2015, 40, 21, E1135-43. https://dx.doi.org/10.1097/BRS.0000000000001040.
#1384	Bastrom 2015	Bastrom, Tracey P; Bartley, Carrie; Marks, Michelle C; Yaszay, Burt; Newton, Peter O; Harms Study Group. Postoperative Perfection: Ceiling Effects and Lack of Discrimination With Both SRS-22 and -24 Outcomes Instruments in Patients With Adolescent Idiopathic Scoliosis. Spine, 2015, 40, 24, E1323-9. https://dx.doi.org/10.1097/BRS.0000000000001082.
#1386	Xu 2015	Xu, Ximing; Wang, Fei; Yang, Mingyuan; Huang, Qikai; Chang, Yifan; Wei, Xianzhao; Bai, Yushu; Li, Ming. Chinese Adaptation of the Bad Sobernheim Stress Questionnaire for Patients With Adolescent Idiopathic Scoliosis Under Brace Treatment. Medicine, 2015, 94, 31, e1236. https://dx.doi.org/10.1097/MD.0000000000001236.
#1392	Durmala 2015	Durmala, Jacek; Blicharska, Irmina; Drosdzol-Cop, Agnieszka; Skrzypulec-Plinta, Violetta. The Level of Self-Esteem and Sexual Functioning in Women with Idiopathic Scoliosis: A Preliminary Study. International journal of environmental research and public health, 2015, 12, 8, 9444-53. https://dx.doi.org/10.3390/ijerph120809444.

#1397	Uddin 2015	Uddin, Omar M; Haque, Raqeeb; Sugrue, Patrick A; Ahmed, Yousef M; El Ahmadieh, Tarek Y; Press, Joel M; Koski, Tyler; Fessler, Richard G. Cost minimization in treatment of adult degenerative scoliosis. Journal of neurosurgery. Spine, 2015, 23, 6, 798-806. https://dx.doi.org/10.3171/2015.3.SPINE14560.
#1408	Zapata 2015	Zapata, Karina Amani; Wang-Price, Sharon S; Sucato, Daniel J; Thompson, Mary; Trudelle-Jackson, Elaine; Lovelace-Chandler, Venita. Spinal Stabilization Exercise Effectiveness for Low Back Pain in Adolescent Idiopathic Scoliosis: A Randomized Trial. Pediatric physical therapy : the official publication of the Section on Pediatrics of the American Physical Therapy Association, 2015, 27, 4, 396-402. https://dx.doi.org/10.1097/PEP.0000000000000174.
#1422	Bao 2015	Bao, Hongda; Yan, Peng; Lonner, Baron; Qiu, Yong; Ren, Yuan; Zhu, Zezhang; Liu, Zhen; Zhu, Feng. Validation of the Simplified Chinese Version of the Body Image Disturbance Questionnaire-Scoliosis. Spine, 2015, 40, 21, E1155-60. https://dx.doi.org/10.1097/BRS.0000000000001140.
#1426	Anwer 2015	Anwer, Shahnawaz; Alghadir, Ahmad; Abu Shaphe, Md; Anwar, Dilshad. Effects of Exercise on Spinal Deformities and Quality of Life in Patients with Adolescent Idiopathic Scoliosis. BioMed research international, 2015, 2015, 101600173, 123848. https://dx.doi.org/10.1155/2015/123848.
#1435	Daniels 2015	Daniels, Alan H; Smith, Justin S; Hiratzka, Jayme; Ames, Christopher P; Bess, Shay; Shaffrey, Christopher I; Schwab, Frank J; Lafage, Virginie; Klineberg, Eric O; Burton, Doug; Mundis, Greg M; Line, Breton; Hart, Robert A; International Spine Study Group (ISSG). Functional Limitations Due to Lumbar Stiffness in Adults With and Without Spinal Deformity. Spine, 2015, 40, 20, 1599-604. https://dx.doi.org/10.1097/BRS.0000000000001090.
#1445	Kleinstueck 2016	Kleinstueck, F S; Fekete, T F; Jeszenszky, D; Haschtmann, D; Mannion, A F. Adult degenerative scoliosis: comparison of patient-rated outcome after three different surgical treatments. European spine journal : official publication of the European Spine Society, the European Spinal Deformity Society, and the European Section of the Cervical Spine Research Society, 2016, 25, 8, 2649-56. https://dx.doi.org/10.1007/s00586-014-3484-7.
#1447	Sciubba 2016	Sciubba, Daniel M; Scheer, Justin K; Yurter, Alp; Smith, Justin S; Lafage, Virginie; Klineberg, Eric; Gupta, Munish; Eastlack, Robert; Mundis, Gregory M; Protopsaltis, Themistocles S; Blaskiewicz, Donald; Kim, Han Jo; Koski, Tyler; Kebaish, Khaled; Shaffrey, Christopher I; Bess, Shay; Hart, Robert A; Schwab, Frank; Ames, Christopher P; International Spine Study Group (ISSG). Patients with spinal deformity over the age of 75: a retrospective analysis of operative versus non-operative management. European spine journal : official publication of the European Spine Society, the European Spinal Deformity Society, and the European Section of the Cervical Spine Research Society, 2016,
#1450	Kuru 2016	Kuru, Tugba; Yeldan, Ipek; Dereli, E Elcin; Ozdincler, Arzu R; Dikici, Fatih; Colak, Ilker. The efficacy of three-dimensional Schroth exercises in adolescent idiopathic scoliosis: a randomised controlled clinical trial. Clinical rehabilitation, 2016, 30, 2, 181-90. https://dx.doi.org/10.1177/0269215515575745.

#1454	Matamalas 2016	Matamalas, Antonia; Bago, Juan; D'Agata, Elisabetta; Pellise, Ferran. Does patient perception of shoulder balance correlate with clinical balance?. European spine journal : official publication of the European Spine Society, the European Spinal Deformity Society, and the European Section of the Cervical Spine Research Society, 2016, 25, 11, 3560-3567. .
#1460	Ferrero 2016	Ferrero, Emmanuelle; Vira, Shaleen; Ames, Christopher P; Kebaish, Khaled; Obeid, Ibrahim; O'Brien, Michael F; Gupta, Munish C; Boachie-Adjei, Oheneba; Smith, Justin S; Mundis, Gregory M; Challier, Vincent; Protopsaltis, Themistocles S; Schwab, Frank J; Lafage, Virginie; International Spine Study Group. Analysis of an unexplored group of sagittal deformity patients: low pelvic tilt despite positive sagittal malalignment. European spine journal : official publication of the European Spine Society, the European Spinal Deformity Society, and the European Section of the Cervical Spine Research Society, 2016, 25, 11, 3568-3576. .
#1464	Berliner 2013	Berliner, Jonathan L; Verma, Kushagra; Lonner, Baron S; Penn, Phedra U; Bharucha, Neil J. Discriminative validity of the Scoliosis Research Society 22 questionnaire among five curve-severity subgroups of adolescents with idiopathic scoliosis. The spine journal : official journal of the North American Spine Society, 2013, 13, 2, 127-33. https://dx.doi.org/10.1016/j.spinee.2012.10.025.
#1484	Kim 2013	Kim, Han Jo; Buchowski, Jacob M; Zebala, Lukas P; Dickson, Douglas D; Koester, Linda; Bridwell, Keith H. RhBMP-2 is superior to iliac crest bone graft for long fusions to the sacrum in adult spinal deformity: 4- to 14-year follow-up. Spine, 2013, 38, 14, 1209-15. https://dx.doi.org/10.1097/BRS.0b013e31828b656d.
#1490	Lansford 2013	Lansford, Todd J; Burton, Douglas C; Asher, Marc A; Lai, Sue-Min. Radiographic and patient-based outcome analysis of different bone-grafting techniques in the surgical treatment of idiopathic scoliosis with a minimum 4-year follow-up: allograft versus autograft/allograft combination. The spine journal : official journal of the North American Spine Society, 2013, 13, 5, 523-9. https://dx.doi.org/10.1016/j.spinee.2013.01.025.
#1493	Daubs 2013	Daubs, Michael D; Lenke, Lawrence G; Bridwell, Keith H; Kim, Yongjung J; Hung, Man; Cheh, Gene; Koester, Linda A. Does correction of preoperative coronal imbalance make a difference in outcomes of adult patients with deformity?. Spine, 2013, 38, 6, 476-83. https://dx.doi.org/10.1097/BRS.0b013e3182846eb3.
#1499	He 2016	He, Shouyu; Bao, Hongda; Zhu, Zezhang; Qiu, Yong; Zhu, Feng; Zhou, Hengcai; Sun, Xu; Wang, Bin. Vertebral coplanar alignment technique: a surgical option for correction of adult thoracic idiopathic scoliosis. European spine journal : official publication of the European Spine Society, the European Spinal Deformity Society, and the European Section of the Cervical Spine Research Society, 2016, 25, 2, 417-23. https://dx.doi.org/10.1007/s00586-015-4080-1.
#1507	Ketenci 2016	Ketenci, Ismail Emre; Yanik, Hakan Serhat; Demiroz, Serdar; Ulusoy, Ayhan; Erdem, Sevki. Three-Dimensional Correction in Patients With Lenke 1 Adolescent Idiopathic Scoliosis: Comparison of Consecutive Versus Interval Pedicle Screw Instrumentation. Spine, 2016, 41, 2, 134-8. https://dx.doi.org/10.1097/BRS.0000000000001182.

#1509	Mummaneni 2016	Mummaneni, Praveen V; Park, Paul; Fu, Kai-Ming; Wang, Michael Y; Nguyen, Stacie; Lafage, Virginie; Uribe, Juan S; Ziewacz, John; Terran, Jamie; Okonkwo, David O; Anand, Neel; Fessler, Richard; Kanter, Adam S; LaMarca, Frank; Deviren, Vedat; Bess, R Shay; Schwab, Frank J; Smith, Justin S; Akbarnia, Behrooz A; Mundis, Gregory M Jr; Shaffrey, Christopher I; International Spine Study Group. Does Minimally Invasive Percutaneous Posterior Instrumentation Reduce Risk of Proximal Junctional Kyphosis in Adult Spinal Deformity Surgery? A Propensity-Matched Cohort Analysis. Neurosurgery, 2016, 78, 1, 101-8. https://dx.doi.org/10.1227/NEU.0000000000001002.
#1517	Park 2016	Park, Paul; Okonkwo, David O; Nguyen, Stacie; Mundis, Gregory M Jr; Than, Khoi D; Deviren, Vedat; La Marca, Frank; Fu, Kai-Ming; Wang, Michael Y; Uribe, Juan S; Anand, Neel; Fessler, Richard; Nunley, Pierce D; Chou, Dean; Kanter, Adam S; Shaffrey, Christopher I; Akbarnia, Behrooz A; Passias, Peter G; Eastlack, Robert K; Mummaneni, Praveen V; International Spine Study Group. Can a Minimal Clinically Important Difference Be Achieved in Elderly Patients with Adult Spinal Deformity Who Undergo Minimally Invasive Spinal Surgery?. World neurosurgery, 2016, 86, 101528275, 168-72. https://dx.doi.org/10.1016/j.wneu.2015.09.072.
#1520	Mannion 2016	Mannion, A F; Vila-Casademunt, A; Domingo-Sabat, M; Wunderlin, S; Pellise, F; Bago, J; Acaroglu, E; Alanay, A; Perez-Grueso, F S; Obeid, I; Kleinstuck, F S; European Spine Study Group (ESSG). The Core Outcome Measures Index (COMI) is a responsive instrument for assessing the outcome of treatment for adult spinal deformity. European spine journal : official publication of the European Spine Society, the European Spinal Deformity Society, and the European Section of the Cervical Spine Research Society, 2016, 25, 8, 2638-48. https://dx.doi.org/10.1007/s00586-015-4292-4.
#1522	Liu 2016	Liu, Shian; Diebo, Bassel G; Henry, Jensen K; Smith, Justin S; Hostin, Richard; Cunningham, Matthew E; Mundis, Gregory; Ames, Christopher P; Burton, Douglas; Bess, Shay; Akbarnia, Behrooz; Hart, Robert; Passias, Peter G; Schwab, Frank J; Lafage, Virginie; International Spine Study Group (ISSG). The benefit of nonoperative treatment for adult spinal deformity: identifying predictors for reaching a minimal clinically important difference. The spine journal : official journal of the North American Spine Society, 2016, 16, 2, 210-8. https://dx.doi.org/10.1016/j.spinee.2015.10.043.
#1523	Schwieger 2016	Schwieger, Traci; Campo, Shelly; Weinstein, Stuart L; Dolan, Lori A; Ashida, Sato; Steuber, Keli R. Body Image and Quality-of-Life in Untreated Versus Brace-Treated Females With Adolescent Idiopathic Scoliosis. Spine, 2016, 41, 4, 311-9. https://dx.doi.org/10.1097/BRS.0000000000001210.
#1527	Bess 2016	Bess, Shay; Line, Breton; Fu, Kai-Ming; McCarthy, Ian; Lafage, Virgine; Schwab, Frank; Shaffrey, Christopher; Ames, Christopher; Akbarnia, Behrooz; Jo, Han; Kelly, Michael; Burton, Douglas; Hart, Robert; Klineberg, Eric; Kebaish, Khaled; Hostin, Richard; Mundis, Gregory; Mummaneni, Praveen; Smith, Justin S; International Spine Study Group. The Health Impact of Symptomatic Adult Spinal Deformity: Comparison of Deformity Types to United States Population Norms and Chronic Diseases. Spine, 2016, 41, 3, 224-33. https://dx.doi.org/10.1097/BRS.0000000000001202.
#1528	Ha 2016	Ha, Kee-Yong; Jang, Won-Hee; Kim, Young-Hoon; Park, Dong-Chul. Clinical Relevance of the SRS-Schwab Classification for Degenerative Lumbar Scoliosis. Spine, 2016, 41, 5, E282-8. https://dx.doi.org/10.1097/BRS.0000000000001229.
#1539	Klineberg 2016	Klineberg, Eric O; Passias, Peter G; Jalai, Cyrus M; Worley, Nancy; Sciubba, Daniel M; Burton, Douglas C; Gupta, Munish C; Soroceanu, Alex; Zebala, Luke P; Mundis, Gregory M Jr; Kim, Han Jo; Hamilton, D Kojo; Hart, Robert A; Ames, Christopher P; Lafage, Virginie; International Spine Study Group. Predicting Extended Length of Hospital Stay in an Adult Spinal Deformity Surgical Population. Spine, 2016, 41, 13, E798-805. https://dx.doi.org/10.1097/BRS.0000000000001391.

#1541	Lafage 2016	Lafage, Renaud; Schwab, Frank; Challier, Vincent; Henry, Jensen K; Gum, Jeffrey; Smith, Justin; Hostin, Richard; Shaffrey, Christopher; Kim, Han J; Ames, Christopher; Scheer, Justin; Klineberg, Eric; Bess, Shay; Burton, Douglas; Lafage, Virginie; International Spine Study Group. Defining Spino-Pelvic Alignment Thresholds: Should Operative Goals in Adult Spinal Deformity Surgery Account for Age?. Spine, 2016, 41, 1, 22859. https://dx.doi.org/10.1097/BRS.0000000000001171.
#1543	Park 2016	Park, Paul; Wang, Michael Y; Nguyen, Stacie; Mundis, Gregory M Jr; La Marca, Frank; Uribe, Juan S; Anand, Neel; Okonkwo, David O; Kanter, Adam S; Fessler, Richard; Eastlack, Robert K; Chou, Dean; Deviren, Vedat; Nunley, Pierce D; Shaffrey, Christopher I; Mummaneni, Praveen V; International Spine Study Group. Comparison of Complications and Clinical and Radiographic Outcomes Between Nonobese and Obese Patients with Adult Spinal Deformity Undergoing Minimally Invasive Surgery. World neurosurgery, 2016, 87, 101528275, 55-60. https://dx.doi.org/10.1016/j.wneu.2015.12.024.
#1558	Madhavan 2016	Madhavan, Karthik; Chieng, Lee Onn; McGrath, Lynn; Hofstetter, Christoph P; Wang, Michael Y. Early experience with endoscopic foraminotomy in patients with moderate degenerative deformity. Neurosurgical focus, 2016, 40, 2, E6. https://dx.doi.org/10.3171/2015.11.FOCUS15511.
#1560	Jiang 2016	Jiang, Chuan-jie; Yang, Yong-jun; Zhou, Ji-ping; Yao, Shu-qiang; Yang, Kai; Wu, Rui; Tan, Yuan-chao. Applications of the scoliosis width-to-length ratio for guiding selection of the surgical approaches of degenerative lumbar scoliosis. BMC musculoskeletal disorders, 2016, 17, 100968565, 48. https://dx.doi.org/10.1186/s12891-016-0904-3.
#1572	Rullander 2016	Rullander, Anna-Clara; Lundstrom, Mats; Lindkvist, Marie; Hagglof, Bruno; Lindh, Viveca. Stress symptoms among adolescents before and after scoliosis surgery: correlations with postoperative pain. Journal of clinical nursing, 2016, 25, 44750, 1086-94. https://dx.doi.org/10.1111/jocn.13137.
#1574	Fortin 2016	Fortin, Carole; Grunstein, Erin; Labelle, Hubert; Parent, Stefan; Ehrmann Feldman, Debbie. Trunk imbalance in adolescent idiopathic scoliosis. The spine journal : official journal of the North American Spine Society, 2016, 16, 6, 687-93. https://dx.doi.org/10.1016/j.spinee.2016.02.033.
#1576	Jain 2016	Jain, Amit; Lafage, Virginie; Kelly, Michael P; Hassanzadeh, Hamid; Neuman, Brian J; Sciubba, Daniel M; Bess, Shay; Shaffrey, Christopher I; Ames, Christopher P; Scheer, Justin K; Burton, Douglas; Gupta, Munish C; Hart, Robert; Hostin, Richard A; Kebaish, Khaled M; International Spine Study Group. Validity, Reliability, and Responsiveness of SRS-7 as an Outcomes Assessment Instrument for Operatively Treated Patients With Adult Spinal Deformity. Spine, 2016, 41, 18, 1463-8. https://dx.doi.org/10.1097/BRS.0000000000001540.
#1582	Matamalas 2016	Matamalas, Antonia; Bago, Juan; D Agata, Elisabetta; Pellise, Ferran. Validity and reliability of photographic measures to evaluate waistline asymmetry in idiopathic scoliosis. European spine journal : official publication of the European Spine Society, the European Spinal Deformity Society, and the European Section of the Cervical Spine Research Society, 2016, 25, 10, 3170-3179. .

#1583	Mariconda 2016	Mariconda, Massimo; Andolfi, Claudia; Cerbasi, Simone; Servodidio, Valeria. Effect of surgical correction of adolescent idiopathic scoliosis on the quality of life: a prospective study with a minimum 5-year follow-up. European spine journal : official publication of the European Spine Society, the European Spinal Deformity Society, and the European Section of the Cervical Spine Research Society, 2016, 25, 10, 3331-3340. .
#1589	Hostin 2016	Hostin, Richard; Robinson, Chessie; O'Brien, Michael; Ames, Christopher; Schwab, Frank; Smith, Justin S; Lafage, Virginie; Gupta, Munish; Hart, Robert; Burton, Douglas; Bess, Shay; Schaffrey, Christopher; McCarthy, Ian. A Multicenter Comparison of Inpatient Resource Use for Adult Spinal Deformity Surgery. Spine, 2016, 41, 7, 603-9. https://dx.doi.org/10.1097/BRS.0000000000001280.
#1592	Monticone 2016	Monticone, Marco; Ambrosini, Emilia; Cazzaniga, Daniele; Rocca, Barbara; Motta, Lorenzo; Cerri, Cesare; Brayda-Bruno, Marco; Lovi, Alessio. Adults with idiopathic scoliosis improve disability after motor and cognitive rehabilitation: results of a randomised controlled trial. European spine journal : official publication of the European Spine Society, the European Spinal Deformity Society, and the European Section of the Cervical Spine Research Society, 2016, 25, 10, 3120-3129. .
#1604	Jalai 2016	Jalai, Cyrus M; Passias, Peter G; Lafage, Virginie; Smith, Justin S; Lafage, Renaud; Poorman, Gregory W; Diebo, Bassel; Liabaud, Barthelemy; Neuman, Brian J; Scheer, Justin K; Shaffrey, Christopher I; Bess, Shay; Schwab, Frank; Ames, Christopher P; International Spine Study Group (ISSG). A comparative analysis of the prevalence and characteristics of cervical malalignment in adults presenting with thoracolumbar spine deformity based on variations in treatment approach over 2 years. European spine journal : official publication of the European Spine Society, the European Spinal Deformity Society, and the European Section of the Cervical Spine Research Society, 2016,
#1605	Wang 2016	Wang, Guodong; Cui, Xingang; Jiang, Zhensong; Li, Tao; Liu, Xiaoyang; Sun, Jianmin. Evaluation and Surgical Management of Adult Degenerative Scoliosis Associated With Lumbar Stenosis. Medicine, 2016, 95, 15, e3394. https://dx.doi.org/10.1097/MD.0000000000003394.
#1613	Hawasli 2016	Hawasli, Ammar H; Chang, Jodie; Yarbrough, Chester K; Steger-May, Karen; Lenke, Lawrence G; Dorward, Ian G. Interpedicular height as a predictor of radicular pain in adult degenerative scoliosis. The spine journal : official journal of the North American Spine Society, 2016, 16, 9, 1070-8. https://dx.doi.org/10.1016/j.spinee.2016.04.017.
#1616	Hostin 2016	Hostin, Richard; O'Brien, Michael; McCarthy, Ian; Bess, Shay; Gupta, Munish; Klineberg, Eric; International Spine Study Group, Denver, CO. Retrospective Study of Anterior Interbody Fusion Rates and Patient Outcomes of Using Mineralized Collagen and Bone Marrow Aspirate in Multilevel Adult Spinal Deformity Surgery. Clinical spine surgery, 2016, 29, 8, E384-8. https://dx.doi.org/10.1097/BSD.0b013e318292468f.
#1640	Negrini 2016	Negrini, Stefano; Minozzi, Silvia; Bettany-Saltikov, Josette; Chockalingam, Nachiappan; Grivas, Theodoros B; Kotwicki, Tomasz; Maruyama, Toru; Romano, Michele; Zaina, Fabio. Braces for Idiopathic Scoliosis in Adolescents. Spine, 2016, 41, 23, 1813-1825. .

#1646	Grant 2016	Grant, Daniel R; Schoenleber, Scott J; McCarthy, Alicia M; Neiss, Geraldine I; Yorgova, Petya K; Rogers, Kenneth J; Gabos, Peter G; Shah, Suken A. Are We Prescribing Our Patients Too Much Pain Medication? Best Predictors of Narcotic Usage After Spinal Surgery for Scoliosis. The Journal of bone and joint surgery. American volume, 2016, 98, 18, 1555-62. https://dx.doi.org/10.2106/JBJS.16.00101.
#1647	Du 2016	Du, Chunping; Yu, Jiadan; Zhang, Jiaqi; Jiang, Jiaojiao; Lai, Huabin; Liu, Wei; Liu, Yang; Li, Hao; Wang, Pu. Relevant areas of functioning in patients with adolescent idiopathic scoliosis on the International Classification of Functioning, Disability and Health: The patients' perspective. Journal of rehabilitation medicine, 2016, 48, 9, 806-814. https://dx.doi.org/10.2340/16501977-2147.
#1650	Banno 2016	Banno, Tomohiro; Hasegawa, Tomohiko; Yamato, Yu; Kobayashi, Sho; Togawa, Daisuke; Oe, Shin; Mihara, Yuki; Matsuyama, Yukihiro. T1 Pelvic Angle Is a Useful Parameter for Postoperative Evaluation in Adult Spinal Deformity Patients. Spine, 2016, 41, 21, 1641-1648. .
#1652	Chapman 2016	Chapman, Todd M Jr; Baldus, Christine R; Lurie, Jon D; Glassman, Steven D; Schwab, Frank J; Shaffrey, Christopher I; Lafage, Virginie; Boachie-Adjei, Oheneba; Kim, Han J; Smith, Justin S; Crawford, Charles H 3rd; Lenke, Lawrence G; Buchowski, Jacob M; Edwards, Charles 2nd; Koski, Tyler; Parent, Stefan; Lewis, Stephen; Kang, Daniel G; McClendon, Jamal Jr; Metz, Lionel; Zebala, Lukas P; Kelly, Michael P; Spratt, Kevin F; Bridwell, Keith H. Baseline Patient-Reported Outcomes Correlate Weakly With Radiographic Parameters: A Multicenter, Prospective NIH Adult Symptomatic Lumbar Scoliosis Study of 286 Patients. Spine, 2016, 41, 22, 1701-1708. .
#1657	Harris 2016	Harris, Bradley Y; Roth, Matthew F; Diebo, Bassel G; Bess, Shay; Theologis, Alexander A; Scheer, Justin K; Schwab, Frank J; Lafage, Virginie; Ames, Christopher P; Hodes, Richard; Ayamga, Jennifer; Boachie-Adjei, Oheneba; International Spine Study Group. Investigating the Universality of Preoperative Health-Related Quality of Life (HRQoL) for Surgically Treated Spinal Deformity in Young Adults: A Propensity Score-Matched Comparison Between African and US Populations. Spine deformity, 2016, 4, 5, 351-357. https://dx.doi.org/10.1016/j.jspd.2016.03.006.
#1660	Crawford 2016	Crawford, Charles H 3rd; Glassman, Steven D; Bridwell, Keith H; Carreon, Leah Y. The Substantial Clinical Benefit Threshold for SRS-22R Domains After Surgical Treatment of Adult Spinal Deformity. Spine deformity, 2016, 4, 5, 373-377. https://dx.doi.org/10.1016/j.jspd.2016.05.001.
#1663	Pizones 2016	Pizones, Javier; Martin-Buitrago, Mar Perez; Sanchez Perez-Grueso, Francisco Javier; Pellise, Ferran; Alanay, Ahmet; Obeid, Ibrahim; Kleinstuck, Frank; Acaroglu, Emre R; ESSG European Spine Study Group. Untreated Thoracic Curve in Adult Idiopathic Scoliosis: What Are Patients' Concerns?. Spine deformity, 2016, 4, 6, 439-445. https://dx.doi.org/10.1016/j.jspd.2016.05.004.
#1669	Ersen 2016	Ersen, O; Bilgic, S; Koca, K; Ege, T; Oguz, E; Bilekli, A B. Difference between Spinecor brace and Thoracolumbosacral orthosis for deformity correction and quality of life in adolescent idiopathic scoliosis. Acta orthopaedica Belgica, 2016, 82, 4, 710-714. .

#1679	Monticone 2004	Monticone, M; Carabalona, R; Negrini, S. Reliability of the Scoliosis Research Society-22 Patient Questionnaire (Italian version) in mild adolescent vertebral deformities. Europa medicophysica, 2004, 40, 3, 191-7. .
#1690	Vasiliadis 2006	Vasiliadis, Elias; Grivas, Theodoros B; Gkoltsiou, Konstantina. Development and preliminary validation of Brace Questionnaire (BrQ): a new instrument for measuring quality of life of brace treated scoliotics. Scoliosis, 2006, 1, 101271527, 7. .
#1692	Rowe 2006	Rowe, Dale E; Feise, Ronald J; Crowther, Edward R; Grod, Jaroslaw P; Menke, J Michael; Goldsmith, Charles H; Stoline, Michael R; Souza, Thomas A; Kambach, Brandon. Chiropractic manipulation in adolescent idiopathic scoliosis: a pilot study. Chiropractic & osteopathy, 2006, 14, 101245797, 15. .
#1694	Pineda 2006	Pineda, Sonia; Bago, Juan; Gilperez, Carmen; Climent, Jose M. Validity of the Walter Reed Visual Assessment Scale to measure subjective perception of spine deformity in patients with idiopathic scoliosis. Scoliosis, 2006, 1, 101271527, 18. .
#1697	Kotwicki 2007	Kotwicki, Tomasz; Kinel, Edyta; Stryla, Wanda; Szulc, Andrzej. Estimation of the stress related to conservative scoliosis therapy: an analysis based on BSSQ questionnaires. Scoliosis, 2007, 2, 101271527, 1. .
#1705	Zhao 2007	Zhao, Li; Zhang, Yong; Sun, Xiaotang; Du, Qing; Shang, Lei. The Scoliosis Research Society-22 questionnaire adapted for adolescent idiopathic scoliosis patients in China: reliability and validity analysis. Journal of children's orthopaedics, 2007, 1, 6, 351-5. https://dx.doi.org/10.1007/s11832-007-0061-1.
#1726	Cahill 2014	Cahill, Patrick J; Pahys, Joshua M; Asghar, Jahangir; Yaszay, Burt; Marks, Michelle C; Bastrom, Tracey P; Lonner, Baron S; Shah, Suken A; Shufflebarger, Harry L; Newton, Peter O; Betz, Randal R; Samdani, Amer F. The effect of surgeon experience on outcomes of surgery for adolescent idiopathic scoliosis. The Journal of bone and joint surgery. American volume, 2014, 96, 16, 1333-9. https://dx.doi.org/10.2106/JBJS.M.01265.
#1739	Fu 2014	Fu, Lingjie; Chang, Michael S; Crandall, Dennis G; Revella, Jan. Does obesity affect surgical outcomes in degenerative scoliosis?. Spine, 2014, 39, 24, 2049-55. https://dx.doi.org/10.1097/BRS.0000000000000600.

#1749	Protopsaltis 2014	Protopsaltis, Themistocles; Schwab, Frank; Bronsard, Nicolas; Smith, Justin S; Klineberg, Eric; Mundis, Gregory; Ryan, Devon J; Hostin, Richard; Hart, Robert; Burton, Douglas; Ames, Christopher; Shaffrey, Christopher; Bess, Shay; Errico, Thomas; Lafage, Virginie; International Spine Study Group. TheT1 pelvic angle, a novel radiographic measure of global sagittal deformity, accounts for both spinal inclination and pelvic tilt and correlates with health-related quality of life. The Journal of bone and joint surgery. American volume, 2014, 96, 19, 1631-40. https://dx.doi.org/10.2106/JBJS.M.01459.
#1755	Plaszewski 2014	Plaszewski, Maciej; Cieslinski, Igor; Kowalski, Pawel; Truszczynska, Aleksandra; Nowobilski, Roman. Does scoliosis-specific exercise treatment in adolescence alter adult quality of life?. TheScientificWorldJournal, 2014, 2014, 101131163, 539671. https://dx.doi.org/10.1155/2014/539671.
#1762	Rushton 2015	Rushton, Paul R P; Grevitt, Michael P; Sell, Philip J. Anterior or posterior surgery for right thoracic adolescent idiopathic scoliosis (AIS)? A prospective cohorts' comparison using radiologic and functional outcomes. Journal of spinal disorders & techniques, 2015, 28, 3, 29434. https://dx.doi.org/10.1097/BSD.0b013e3182693e33.
#1766	Zavatsky 2015	Zavatsky, Joseph M; Peters, Austin J; Nahvi, Farzon A; Bharucha, Neil J; Trobisch, Per D; Kean, Kristin E; Richard, Sandra; Bucello, Yolanda; Valdevit, Antonio; Lonner, Baron S. Disease severity and treatment in adolescent idiopathic scoliosis: the impact of race and economic status. The spine journal : official journal of the North American Spine Society, 2015, 15, 5, 939-43. https://dx.doi.org/10.1016/j.spinee.2013.06.043.
#1776	Pellise 2015	Pellise, Ferran; Vila-Casademunt, Alba; Ferrer, Montse; Domingo-Sabat, Montse; Bago, Juan; Perez-Grueso, Francisco J S; Alanay, Ahmet; Mannion, A F; Acaroglu, Emre; European Spine Study Group, ESSG. Impact on health related quality of life of adult spinal deformity (ASD) compared with other chronic conditions. European spine journal : official publication of the European Spine Society, the European Spinal Deformity Society, and the European Section of the Cervical Spine Research Society, 2015, 24, 1, 44631. https://dx.doi.org/10.1007/s00586-014-3542-1.
#1782	McCarthy 2015	McCarthy, Ian M. Putting the Patient in Patient Reported Outcomes: A Robust Methodology for Health Outcomes Assessment. Health economics, 2015, 24, 12, 1588-603. https://dx.doi.org/10.1002/hec.3113.
#1791	Rainoldi 2015	Rainoldi, Laura; Zaina, Fabio; Villafane, Jorge H; Donzelli, Sabrina; Negrini, Stefano. Quality of life in normal and idiopathic scoliosis adolescents before diagnosis: reference values and discriminative validity of the SRS-22. A cross-sectional study of 1,205 pupils. The spine journal : official journal of the North American Spine Society, 2015, 15, 4, 662-7. https://dx.doi.org/10.1016/j.spinee.2014.12.004.
#1798	Albayrak 2015	Albayrak, Akif; Buyuk, Abdul Fettah; Ucpunar, Hanifi; Balioglu, Mehmet Bulent; Kargin, Deniz; Kaygusuz, Mehmet Akif. Pre- and postoperative photographs and surgical outcomes in patients with Lenke type 1 adolescent idiopathic scoliosis. Spine, 2015, 40, 7, 469-74. https://dx.doi.org/10.1097/BRS.0000000000000796.

#1804	Park 2015	Park, Paul; Wang, Michael Y; Lafage, Virginie; Nguyen, Stacie; Ziewacz, John; Okonkwo, David O; Uribe, Juan S; Eastlack, Robert K; Anand, Neel; Haque, Raqeeb; Fessler, Richard G; Kanter, Adam S; Deviren, Vedat; La Marca, Frank; Smith, Justin S; Shaffrey, Christopher I; Mundis, Gregory M Jr; Mummaneni, Praveen V; International Spine Study Group. Comparison of two minimally invasive surgery strategies to treat adult spinal deformity. Journal of neurosurgery. Spine, 2015, 22, 4, 374-80. https://dx.doi.org/10.3171/2014.9.SPINE131004.
#1814	Scheer 2015	Scheer, Justin K; Smith, Justin S; Clark, Aaron J; Lafage, Virginie; Kim, Han Jo; Rolston, John D; Eastlack, Robert; Hart, Robert A; Protopsaltis, Themistocles S; Kelly, Michael P; Kebaish, Khaled; Gupta, Munish; Klineberg, Eric; Hostin, Richard; Shaffrey, Christopher I; Schwab, Frank; Ames, Christopher P; International Spine Study Group. Comprehensive study of back and leg pain improvements after adult spinal deformity surgery: analysis of 421 patients with 2-year follow-up and of the impact of the surgery on treatment satisfaction. Journal of neurosurgery. Spine, 2015, 22, 5, 540-53. https://dx.doi.org/10.3171/2014.10.SPINE14475.
#1818	Crawford 2015	Crawford, Charles H 3rd; Glassman, Steven D; Bridwell, Keith H; Berven, Sigurd H; Carreon, Leah Y. The minimum clinically important difference in SRS-22R total score, appearance, activity and pain domains after surgical treatment of adult spinal deformity. Spine, 2015, 40, 6, 377-81. https://dx.doi.org/10.1097/BRS.0000000000000761.
#1819	Aykac 2015	Aykac, Bilal; Ayhan, Selim; Yuksel, Selcen; Guler, Umit Ozgur; Pellise, Ferran; Alanay, Ahmet; Perez-Grueso, Francisco Javier Sanchez; Acaroglu, Emre; ESSG European Spine Study Group. Sagittal alignment of cervical spine in adult idiopathic scoliosis. European spine journal : official publication of the European Spine Society, the European Spinal Deformity Society, and the European Section of the Cervical Spine Research Society, 2015, 24, 6, 1175-82. https://dx.doi.org/10.1007/s00586-015-3868-3.
#1824	Passias 2015	Passias, Peter G; Soroceanu, Alexandra; Scheer, Justin; Yang, Sun; Boniello, Anthony; Smith, Justin S; Protopsaltis, Themistocles; Kim, Han J; Schwab, Frank; Gupta, Munish; Klineberg, Eric; Mundis, Gregory; Lafage, Renaud; Hart, Robert; Shaffrey, Christopher; Lafage, Virginie; Ames, Christopher; International Spine Study Group. Magnitude of preoperative cervical lordotic compensation and C2-T3 angle are correlated to increased risk of postoperative sagittal spinal pelvic malalignment in adult thoracolumbar deformity patients at 2-year follow-up. The spine journal : official journal of the North American Spine Society, 2015, 15, 8, 1756-63.
#1836	Haidar 2015	Haidar, Rachid K; Kassak, Kassem; Masrouha, Karim; Ibrahim, Kamal; Mhaidli, Hani. Reliability and validity of an adapted Arabic version of the Scoliosis Research Society-22r Questionnaire. Spine, 2015, 40, 17, E971-7. https://dx.doi.org/10.1097/BRS.0000000000000956.
#1839	Gum 2015	Gum, Jeffrey L; Bridwell, Keith H; Lenke, Lawrence G; Bumpass, David B; Sugrue, Patrick A; Karikari, Isaac O; Carreon, Leah Y. SRS22R Appearance Domain Correlates Most With Patient Satisfaction After Adult Deformity Surgery to the Sacrum at 5-year Follow-up. Spine, 2015, 40, 16, 1297-302. https://dx.doi.org/10.1097/BRS.0000000000000961.
#1840	Godzik 2015	Godzik, Jakub; Holekamp, Terrence F; Limbrick, David D; Lenke, Lawrence G; Park, T S; Ray, Wilson Z; Bridwell, Keith H; Kelly, Michael P. Risks and outcomes of spinal deformity surgery in Chiari malformation, Type 1, with syringomyelia versus adolescent idiopathic scoliosis. The spine journal : official journal of the North American Spine Society, 2015, 15, 9, 37469. https://dx.doi.org/10.1016/j.spinee.2015.04.048.

#1844	Protopsaltis 2015	Protopsaltis, Themistocles S; Scheer, Justin K; Terran, Jamie S; Smith, Justin S; Hamilton, D Kojo; Kim, Han Jo; Mundis, Greg M Jr; Hart, Robert A; McCarthy, Ian M; Klineberg, Eric; Lafage, Virginie; Bess, Shay; Schwab, Frank; Shaffrey, Christopher I; Ames, Christopher P; International Spine Study Group. How the neck affects the back: changes in regional cervical sagittal alignment correlate to HRQOL improvement in adult thoracolumbar deformity patients at 2-year follow-up. Journal of neurosurgery. Spine, 2015, 23, 2, 153-8. https://dx.doi.org/10.3171/2014.11.SPINE1441.
#1850	Jain 2015	Jain, Amit; Sponseller, Paul D; Negrini, Stefano; Newton, Peter O; Cahill, Patrick J; Bastrom, Tracey P; Marks, Michelle C; Harms Study Group. SRS-7: A Valid, Responsive, Linear, and Unidimensional Functional Outcome Measure for Operatively Treated Patients With AIS. Spine, 2015, 40, 9, 650-5. https://dx.doi.org/10.1097/BRS.0000000000000836.
#1851	Smith 2015	Smith, Justin S; Shaffrey, Christopher I; Lafage, Virginie; Schwab, Frank; Scheer, Justin K; Protopsaltis, Themistocles; Klineberg, Eric; Gupta, Munish; Hostin, Richard; Fu, Kai-Ming G; Mundis, Gregory M Jr; Kim, Han Jo; Deviren, Vedat; Soroceanu, Alex; Hart, Robert A; Burton, Douglas C; Bess, Shay; Ames, Christopher P; International Spine Study Group. Comparison of best versus worst clinical outcomes for adult spinal deformity surgery: a retrospective review of a prospectively collected, multicenter database with 2-year follow-up. Journal of neurosurgery. Spine, 2015, 23, 3, 349-59. https://dx.doi.org/10.3171/2014.12.SPINE14777.
#1863	Lange 2009	Lange, Johan Emil; Steen, Harald; Brox, Jens Ivar. Long-term results after Boston brace treatment in adolescent idiopathic scoliosis. Scoliosis, 2009, 4, 101271527, 17. https://dx.doi.org/10.1186/1748-7161-4-17.
#1870	Bago 2010	Bago, Juan; Sanchez-Raya, Judith; Perez-Grueso, Francisco Javier Sanchez; Climent, Jose Maria. The Trunk Appearance Perception Scale (TAPS): a new tool to evaluate subjective impression of trunk deformity in patients with idiopathic scoliosis. Scoliosis, 2010, 5, 101271527, 6. https://dx.doi.org/10.1186/1748-7161-5-6.
#1874	D'Agata 2010	D'Agata, Elisabetta; Testor, Carles Perez; Rigo, Manuel. Spanish validation of Bad Sobernheim Stress Questionnaire (BSSQ (brace).es) for adolescents with braces. Scoliosis, 2010, 5, 101271527, 15. https://dx.doi.org/10.1186/1748-7161-5-15.
#1879	Aulisa 2010	Aulisa, Angelo G; Guzzanti, Vincenzo; Perisano, Carlo; Marzetti, Emanuele; Specchia, Alessandro; Galli, Marco; Giordano, Marco; Aulisa, Lorenzo. Determination of quality of life in adolescents with idiopathic scoliosis subjected to conservative treatment. Scoliosis, 2010, 5, 101271527, 21. https://dx.doi.org/10.1186/1748-7161-5-21.
#1898	Morningstar 2011	Morningstar, Mark W. Outcomes for adult scoliosis patients receiving chiropractic rehabilitation: a 24-month retrospective analysis. Journal of chiropractic medicine, 2011, 10, 3, 179-84. https://dx.doi.org/10.1016/j.jcm.2011.01.006.

#1901	Nam 2011	Nam, Hee-Seung; Park, Yong Bum. Effects of transforaminal injection for degenerative lumbar scoliosis combined with spinal stenosis. Annals of rehabilitation medicine, 2011, 35, 4, 514-23. https://dx.doi.org/10.5535/arm.2011.35.4.514.
#1903	Davies 2011	Davies, Evan; Norvell, Daniel; Hermsmeyer, Jeffrey. Efficacy of bracing versus observation in the treatment of idiopathic scoliosis. Evidence-based spine-care journal, 2011, 2, 2, 25-34. https://dx.doi.org/10.1055/s-0030-1267102.
#1907	Yu 2012	Yu, Ching-Hsiao; Chen, Po-Quang; Ma, Shu-Chuang; Pan, Chee-Huan. Segmental correction of adolescent idiopathic scoliosis by all-screw fixation method in adolescents and young adults. minimum 5 years follow-up with SF-36 questionnaire. Scoliosis, 2012, 7, 101271527, 5. https://dx.doi.org/10.1186/1748-7161-7-5.
#1923	Potoupnis 2012	Potoupnis, M; Papavasiliou, K; Kenanidis, E; Pellios, S; Kapetanou, A; Sayegh, F; Kapetanos, G. Reliability and concurrent validity of the adapted Greek version of the Scoliosis Research Society-22r Questionnaire. A cross-sectional study performed on conservatively treated patients. Hippokratia, 2012, 16, 3, 225-9. .
#1928	Al-Aubaidi 2013	Al-Aubaidi, Zaid Tj; Tropp, Hans; Pedersen, Niels W; Jespersen, Stig M. Comparison of in-and outpatients protocols for providence night time only bracing in AIS patients - compliance and satisfaction. Scoliosis, 2013, 8, 101271527, 6. https://dx.doi.org/10.1186/1748-7161-8-6.
#1934	Aulisa 2013	Aulisa, Angelo G; Guzzanti, Vincenzo; Galli, Marco; Erra, Carmen; Scudieri, Giorgio; Padua, Luca. Validation of Italian version of Brace Questionnaire (BrQ). Scoliosis, 2013, 8, 1, 13. https://dx.doi.org/10.1186/1748-7161-8-13.
#1952	Chan 2014	Chan, Siu Ling; Cheung, Kenneth Mc; Luk, Keith Dk; Wong, Kenneth Wh; Wong, Man Sang. A correlation study between in-brace correction, compliance to spinal orthosis and health-related quality of life of patients with Adolescent Idiopathic Scoliosis. Scoliosis, 2014, 9, 1, 1. https://dx.doi.org/10.1186/1748-7161-9-1.
#1956	Liu 2014	Liu, Shian; Schwab, Frank; Smith, Justin S; Klineberg, Eric; Ames, Christopher P; Mundis, Gregory; Hostin, Richard; Kebaish, Khaled; Deviren, Vedat; Gupta, Munish; Boachie-Adjei, Oheneba; Hart, Robert A; Bess, Shay; Lafage, Virginie. Likelihood of reaching minimal clinically important difference in adult spinal deformity: a comparison of operative and nonoperative treatment. The Ochsner journal, 2014, 14, 1, 67-77. .

#1960	Rivett 2014	Rivett, LouAnn; Stewart, Aimee; Potterton, Joanne. The effect of compliance to a Rigo System Cheneau brace and a specific exercise programme on idiopathic scoliosis curvature: a comparative study: SOSORT 2014 award winner. Scoliosis, 2014, 9, 101271527, 5. https://dx.doi.org/10.1186/1748-7161-9-5.
#1971	D'Agata 2014	D'Agata, Elisabetta; Rigo, Manuel; Perez-Testor, Carles; Puigvi, Nuria Casanovas; Castellano-Tejedor, Carmina. Emotional indicators in young patients with Idiopathic Scoliosis: a study through the drawing of Human Figure. Scoliosis, 2014, 9, 1, 24. https://dx.doi.org/10.1186/s13013-014-0024-5.
#1995	Pellios 2016	Pellios, Stavros; Kenanidis, Eustathios; Potoupnis, Michael; Tsiridis, Eleftherios; Sayegh, Fares E; Kirkos, John; Kapetanos, George A. Curve progression 25 years after bracing for adolescent idiopathic scoliosis: long term comparative results between two matched groups of 18 versus 23 hours daily bracing. Scoliosis and spinal disorders, 2016, 11, 101675716, 3. https://dx.doi.org/10.1186/s13013-016-0065-z.
#1996	Lee 2016	Lee, Hyejung; Choi, Jihea; Hwang, Jin-Ho; Park, Jung Hyun. Health-related quality of life of adolescents conservatively treated for idiopathic scoliosis in Korea: a cross-sectional study. Scoliosis and spinal disorders, 2016, 11, 101675716, 11. https://dx.doi.org/10.1186/s13013-016-0071-1.
#2000	Diarbakerli 2016	Diarbakerli, Elias; Grauers, Anna; Moller, Hans; Abbott, Allan; Gerdhem, Paul. Adolescents with and without idiopathic scoliosis have similar self-reported level of physical activity: a cross-sectional study. Scoliosis and spinal disorders, 2016, 11, 101675716, 17. https://dx.doi.org/10.1186/s13013-016-0082-y.
#2006	Lebel 2016	Lebel, Andrea; Lebel, Victoria Ashley. Severe progressive scoliosis in an adult female possibly secondary thoracic surgery in childhood treated with scoliosis specific Schroth physiotherapy: Case presentation. Scoliosis and spinal disorders, 2016, 11, Suppl 2, 41. .
#2020	Theis 2017	Theis, Jennifer C; Grauers, Anna; Diarbakerli, Elias; Savvides, Panayiotis; Abbott, Allan; Gerdhem, Paul. An observational study on surgically treated adult idiopathic scoliosis patients' quality of life outcomes at 1- and 2-year follow-ups and comparison to controls. Scoliosis and spinal disorders, 2017, 12, 101675716, 11. https://dx.doi.org/10.1186/s13013-017-0118-y.
#2021	Teles 2017	Teles, Alisson R; Mattei, Tobias A; Righesso, Orlando; Falavigna, Asdrubal. Effectiveness of Operative and Nonoperative Care for Adult Spinal Deformity: Systematic Review of the Literature. Global spine journal, 2017, 7, 2, 170-178. https://dx.doi.org/10.1177/2192568217699182.

#2022	Deceuninck 2017	Deceuninck, Julie; Tirat-Herbert, Aurelie; Rodriguez Martinez, Nuria; Bernard, Jean-Claude. French validation of the Brace Questionnaire (BrQ). Scoliosis and spinal disorders, 2017, 12, 101675716, 18. https://dx.doi.org/10.1186/s13013-017-0126-y.
#2024	Rodrigues 2017	Rodrigues, Luciano Miller Reis; Gotfryd, Alberto Ofenhejm; Machado, Andre Nunes; Defino, Matheus; Asano, Leonardo Yukio Jorge. Adolescent idiopathic scoliosis: surgical treatment and quality of life. Acta ortopedica brasileira, 2017, 25, 3, 85-89. https://dx.doi.org/10.1590/1413-785220172503157788.
#2026	Theroux 2017	Theroux, Jean; Stomski, Norman; Innes, Stanley; Ballard, Ariane; Khadra, Christelle; Labelle, Hubert; Le May, Sylvie. Revisiting the psychometric properties of the Scoliosis Research Society-22 (SRS-22) French version. Scoliosis and spinal disorders, 2017, 12, 101675716, 21. https://dx.doi.org/10.1186/s13013-017-0129-8.
#2028	Lee 2017	Lee, Kyu Yeol; Kim, Min-Woo; Im, Chul Soon; Jung, Young Hoon. Radiologic and Clinical Courses of Degenerative Lumbar Scoliosis (10degree-25degree) after a Short-Segment Fusion. Asian spine journal, 2017, 11, 4, 570-579. https://dx.doi.org/10.4184/asj.2017.11.4.570.
#2029	Kato 2017	Kato, Minori; Namikawa, Takashi; Matsumura, Akira; Konishi, Sadahiko; Nakamura, Hiroaki. Radiographic Risk Factors of Reoperation Following Minimally Invasive Decompression for Lumbar Canal Stenosis Associated With Degenerative Scoliosis and Spondylolisthesis. Global spine journal, 2017, 7, 6, 498-505. https://dx.doi.org/10.1177/2192568217699192.
#2032	Eastlack 2017	Eastlack, Robert K; Mundis, Gregory M Jr; Wang, Michael; Mummaneni, Praveen V; Uribe, Juan; Okonkwo, David; Akbarnia, Behrooz A; Anand, Neel; Kanter, Adam; Park, Paul; Lafage, Virginie; Shaffrey, Christopher; Fessler, Richard; Deviren, Vedat; International Spine Study Group. Is There a Patient Profile That Characterizes a Patient With Adult Spinal Deformity as a Candidate for Minimally Invasive Surgery?. Global spine journal, 2017, 7, 7, 703-708. https://dx.doi.org/10.1177/2192568217716151.
#2033	Kwan 2017	Kwan, Kenny Yat Hong; Cheng, Aldous C S; Koh, Hui Yu; Chiu, Alice Y Y; Cheung, Kenneth Man Chee. Effectiveness of Schroth exercises during bracing in adolescent idiopathic scoliosis: results from a preliminary study-SOSORT Award 2017 Winner. Scoliosis and spinal disorders, 2017, 12, 101675716, 32. https://dx.doi.org/10.1186/s13013-017-0139-6.
#2037	D'Agata 2017	D'Agata, Elisabetta; Sanchez-Raya, Judith; Bago, Juan. Introversion, the prevalent trait of adolescents with idiopathic scoliosis: an observational study. Scoliosis and spinal disorders, 2017, 12, 101675716, 27. https://dx.doi.org/10.1186/s13013-017-0136-9.

#2043	Faraj 2017	Faraj, Sayf S A; Haanstra, Tsjitske M; Martijn, Hugo; de Kleuver, Marinus; van Royen, Barend J. Functional outcome of non-surgical and surgical management for de novo degenerative lumbar scoliosis: a mean follow-up of 10 years. Scoliosis and spinal disorders, 2017, 12, 101675716, 35. https://dx.doi.org/10.1186/s13013-017-0143-x.
#2045	Cheshire 2017	Cheshire, James; Gardner, Adrian; Berryman, Fiona; Pynsent, Paul. Do the SRS-22 self-image and mental health domain scores reflect the degree of asymmetry of the back in adolescent idiopathic scoliosis?. Scoliosis and spinal disorders, 2017, 12, 101675716, 37. https://dx.doi.org/10.1186/s13013-017-0144-9.
#2066	Pratali 2018	Pratali, Raphael de Rezende; Martins, Samuel Machado; Santos, Francisco Prado Eugenio Dos; Barsotti, Carlos Eduardo Goncales; Oliveira, Carlos Eduardo Algaves Soares de. The use of three-column osteotomy in the treatment of rigid deformities of the adult spine. Revista brasileira de ortopedia, 2018, 53, 2, 213-220. https://dx.doi.org/10.1016/j.rboe.2017.03.016.
#2101	Nitikman 2017	Nitikman, Michael; Mulpuri, Kishore; Reilly, Christopher W. Internet-administered Health-related Quality of Life Questionnaires Compared With Pen and Paper in an Adolescent Scoliosis Population: A Randomized Crossover Study. Journal of pediatric orthopedics, 2017, 37, 2, e75-e79. https://dx.doi.org/10.1097/BPO.0000000000000716.
#2104	Schwieger 2017	Schwieger, Traci; Campo, Shelly; Weinstein, Stuart L; Dolan, Lori A; Ashida, Sato; Steuber, Keli R. Body Image and Quality of Life and Brace Wear Adherence in Females With Adolescent Idiopathic Scoliosis. Journal of pediatric orthopedics, 2017, 37, 8, e519-e523. https://dx.doi.org/10.1097/BPO.0000000000000734.
#2105	Souder 2017	Souder, Christopher; Newton, Peter O; Shah, Suken A; Lonner, Baron S; Bastrom, Tracey P; Yaszay, Burt. Factors in Surgical Decision Making for Thoracolumbar/Lumbar AIS: It's About More Than Just the Curve Magnitude. Journal of pediatric orthopedics, 2017, 37, 8, e530-e535. https://dx.doi.org/10.1097/BPO.0000000000000746.
#2114	Paolucci 2017	Paolucci, Teresa; Piccinini, Giulia; Iosa, Marco; Piermattei, Cristina; De Angelis, Simona; Zangrando, Federico; Saraceni, Vincenzo Maria. The importance of trunk perception during brace treatment in moderate juvenile idiopathic scoliosis: What is the impact on self-image?. Journal of back and musculoskeletal rehabilitation, 2017, 30, 2, 203-210. https://dx.doi.org/10.3233/BMR-160733.
#2115	Yoshida 2017	Yoshida, Go; Boissiere, Louis; Larrieu, Daniel; Bourghli, Anouar; Vital, Jean Marc; Gille, Olivier; Pointillart, Vincent; Challier, Vincent; Mariey, Remi; Pellise, Ferran; Vila-Casademunt, Alba; Perez-Grueso, Francisco Javier Sanchez; Alanay, Ahmet; Acaroglu, Emre; Kleinstuck, Frank; Obeid, Ibrahim; ESSG, European Spine Study Group. Advantages and Disadvantages of Adult Spinal Deformity Surgery and Its Impact on Health-Related Quality of Life. Spine, 2017, 42, 6, 411-419. https://dx.doi.org/10.1097/BRS.0000000000001770.

#2126	Nabiyev 2017	Nabiyev, Vugar; Ayhan, Selim; Yuksel, Selcen; Niyazi, Asli; Mmopelwa, Tiro; Domingo-Sabat, Montse; Vila-Casademunt, Alba; Pellise, Ferran; Alanay, Ahmet; Perez-Grueso, Francisco; Kleinstuck, Frank; Obeid, Ibrahim; Acaroglu, Emre; European Spine Study Group (ESSG). Does Surgery for Adult Spinal Deformity Affect the Cognitive Abilities in Patients over 50 Years of Age?. Turkish neurosurgery, 2017, 27, 5, 779-784. https://dx.doi.org/10.5137/1019-5149.JTN.18285-16.1.
#2130	Bakhsheshian 2017	Bakhsheshian, Joshua; Scheer, Justin K; Gum, Jeffrey L; Hostin, Richard; Lafage, Virginie; Bess, Shay; Protopsaltis, Themistocles S; Burton, Douglas C; Keefe, Malla Kate; Hart, Robert A; Mundis, Gregory M Jr; Shaffrey, Christopher I; Schwab, Frank; Smith, Justin S; Ames, Christopher P; International Spine Study Group. Impact of poor mental health in adult spinal deformity patients with poor physical function: a retrospective analysis with a 2-year follow-up. Journal of neurosurgery. Spine, 2017, 26, 1, 116-124. https://dx.doi.org/10.3171/2016.5.SPINE151428.
#2133	Gur 2017	Gur, Gozde; Ayhan, Cigdem; Yakut, Yavuz. The effectiveness of core stabilization exercise in adolescent idiopathic scoliosis: A randomized controlled trial. Prosthetics and orthotics international, 2017, 41, 3, 303-310. https://dx.doi.org/10.1177/0309364616664151.
#2139	Kim 2017	Kim, Yong-Chan; Lenke, Lawrence G; Lee, Seon-Jong; Gum, Jeffrey L; Wilartratsami, Sirichai; Blanke, Kathy M. The cranial sagittal vertical axis (CrSVA) is a better radiographic measure to predict clinical outcomes in adult spinal deformity surgery than the C7 SVA: a monocentric study. European spine journal : official publication of the European Spine Society, the European Spinal Deformity Society, and the European Section of the Cervical Spine Research Society, 2017, 26, 8, 2167-2175. https://dx.doi.org/10.1007/s00586-016-4757-0.
#2140	Hamilton 2017	Hamilton, David Kojo; Kong, Christopher; Hiratzka, Jayme; Contag, Alec G; Ailon, Tamir; Line, Breton; Daniels, Alan; Smith, Justin S; Passias, Peter; Protopsaltis, Themistocles; Sciubba, Daniel; Burton, Douglas; Shaffrey, Christopher; Klineberg, Eric; Mundis, Gregory; Kim, Han-Jo; Lafage, Virginie; Lafage, Renaud; Scheer, Justin; Boachie-Adjei, Oheneba; Bess, Shay; Hart, Robert A. Patient Satisfaction After Adult Spinal Deformity Surgery Does Not Strongly Correlate With Health-Related Quality of Life Scores, Radiographic Parameters, or Occurrence of Complications. Spine, 2017, 42, 10, 764-769. https://dx.doi.org/10.1097/BRS.0000000000001921.
#2142	Yagi 2017	Yagi, Mitsuru; Ohne, Hideaki; Konomi, Tsunehiko; Fujiyoshi, Kanehiro; Kaneko, Shinjiro; Takemitsu, Masakazu; Machida, Masafumi; Yato, Yoshiyuki; Asazuma, Takashi. Walking balance and compensatory gait mechanisms in surgically treated patients with adult spinal deformity. The spine journal : official journal of the North American Spine Society, 2017, 17, 3, 409-417. https://dx.doi.org/10.1016/j.spinee.2016.10.014.
#2143	Theologis 2017	Theologis, Alexander A; Mundis, Gregory M Jr; Nguyen, Stacie; Okonkwo, David O; Mummaneni, Praveen V; Smith, Justin S; Shaffrey, Christopher I; Fessler, Richard; Bess, Shay; Schwab, Frank; Diebo, Bassel G; Burton, Douglas; Hart, Robert; Deviren, Vedat; Ames, Christopher; for the International Spine Study Group. Utility of multilevel lateral interbody fusion of the thoracolumbar coronal curve apex in adult deformity surgery in combination with open posterior instrumentation and L5-S1 interbody fusion: a case-matched evaluation of 32 patients. Journal of neurosurgery. Spine, 2017, 26, 2, 208-219. https://dx.doi.org/10.3171/2016.8.SPINE151543.
#2149	Boissiere 2017	Boissiere, Louis; Takemoto, Mitsuru; Bourghli, Anouar; Vital, Jean-Marc; Pellise, Ferran; Alanay, Ahmet; Yilgor, Caglar; Acaroglu, Emre; Perez-Grueso, Francisco Javier; Kleinstuck, Frank; Obeid, Ibrahim; European Spine Study Group (ESSG). Global tilt and lumbar lordosis index: two parameters correlating with health-related quality of life scores-but how do they truly impact disability?. The spine journal : official journal of the North American Spine Society, 2017, 17, 4, 480-488. https://dx.doi.org/10.1016/j.spinee.2016.10.013.

#2152	McKean 2017	McKean, Greg M; Tsirikos, Athanasios I. Quality of life in children and adolescents undergoing spinal deformity surgery. Journal of back and musculoskeletal rehabilitation, 2017, 30, 2, 339-346. https://dx.doi.org/10.3233/BMR-160558.
#2153	Colak 2017	Colak, Tugba Kuru; Akgul, Turgut; Colak, Ilker; Dereli, Elif Elcin; Chodza, Mehmet; Dikici, Fatih. Health related quality of life and perception of deformity in patients with adolescent idiopathic scoliosis. Journal of back and musculoskeletal rehabilitation, 2017, 30, 3, 597-602. https://dx.doi.org/10.3233/BMR-160564.
#2155	Takemoto 2017	Takemoto, Mitsuru; Boissiere, Louis; Vital, Jean-Marc; Pellise, Ferran; Perez-Grueso, Francisco Javier Sanchez; Kleinstuck, Frank; Acaroglu, Emre R; Alanay, Ahmet; Obeid, Ibrahim. Are sagittal spinopelvic radiographic parameters significantly associated with quality of life of adult spinal deformity patients? Multivariate linear regression analyses for pre-operative and short-term post-operative health-related quality of life. European spine journal : official publication of the European Spine Society, the European Spinal Deformity Society, and the European Section of the Cervical Spine Research Society, 2017, 26, 8, 2176-2186. https://dx.doi.org/10.1007/s00586-016-4872-y.
#2156	Theroux 2017	Theroux, Jean; Le May, Sylvie; Hebert, Jeffrey J; Labelle, Hubert. Back Pain Prevalence Is Associated With Curve-type and Severity in Adolescents With Idiopathic Scoliosis: A Cross-sectional Study. Spine, 2017, 42, 15, E914-E919. https://dx.doi.org/10.1097/BRS.0000000000001986.
#2157	Bakhsheshian 2017	Bakhsheshian, Joshua; Scheer, Justin K; Gum, Jeffrey L; Horner, Lance; Hostin, Richard; Lafage, Virginie; Bess, Shay; Protopsaltis, Themistocles S; Burton, Douglas C; Keefe, Malla; Hart, Robert A; Mundis, Gregory M; Shaffrey, Christopher I; Schwab, Frank; Smith, Justin S; Ames, Christopher P; International Spine Study Group (ISSG). Comparison of Structural Disease Burden to Health-related Quality of Life Scores in 264 Adult Spinal Deformity Patients With 2-Year Follow-up: Novel Insights into Drivers of Disability. Clinical spine surgery, 2017, 30, 2, E124-E131. https://dx.doi.org/10.1097/BSD.0000000000000470.
#2162	Wetterkamp 2017	Wetterkamp, Mark; Thielsch, Meinald T; Gosheger, Georg; Boertz, Patrick; Terheyden, Jan Henrik; Schulte, Tobias L. German validation of the BIDQ-S questionnaire on body image disturbance in idiopathic scoliosis. European spine journal : official publication of the European Spine Society, the European Spinal Deformity Society, and the European Section of the Cervical Spine Research Society, 2017, 26, 2, 309-315. https://dx.doi.org/10.1007/s00586-016-4895-4.
#2165	Zapata 2017	Zapata, Karina A; Wang-Price, Sharon S; Sucato, Daniel J. Six-Month Follow-up of Supervised Spinal Stabilization Exercises for Low Back Pain in Adolescent Idiopathic Scoliosis. Pediatric physical therapy : the official publication of the Section on Pediatrics of the American Physical Therapy Association, 2017, 29, 1, 62-66. .
#2186	Kondo 2017	Kondo, Ryo; Yamato, Yu; Nagafusa, Tetsuyuki; Mizushima, Takashi; Hasegawa, Tomohiko; Kobayashi, Sho; Togawa, Daisuke; Oe, Shin; Kurosu, Kenta; Matsuyama, Yukihiro. Effect of corrective long spinal fusion to the ilium on physical function in patients with adult spinal deformity. European spine journal : official publication of the European Spine Society, the European Spinal Deformity Society, and the European Section of the Cervical Spine Research Society, 2017, 26, 8, 2138-2145. https://dx.doi.org/10.1007/s00586-017-4987-9.

#2199	Jain 2017	Jain, Amit; Kebaish, Khaled M; Sciubba, Daniel M; Hassanzadeh, Hamid; Scheer, Justin K; Neuman, Brian J; Lafage, Virginie; Bess, Shay; Protopsaltis, Themistocles S; Burton, Douglas C; Smith, Justin S; Shaffrey, Christopher I; Hostin, Richard A; Ames, Christopher P; International Spine Study Group. Early Patient-Reported Outcomes Predict 3-Year Outcomes in Operatively Treated Patients with Adult Spinal Deformity. World neurosurgery, 2017, 102, 101528275, 258-262. https://dx.doi.org/10.1016/j.wneu.2017.03.003.
#2202	Voepel-Lewis 2017	Voepel-Lewis, Terri; Caird, Michelle S; Tait, Alan R; Malviya, Shobha; Farley, Frances A; Li, Ying; Abbott, Matthew D; van Veen, Tara; Hassett, Afton L; Clauw, Daniel J. A High Preoperative Pain and Symptom Profile Predicts Worse Pain Outcomes for Children After Spine Fusion Surgery. Anesthesia and analgesia, 2017, 124, 5, 1594-1602. https://dx.doi.org/10.1213/ANE.0000000000001963.
#2204	Atici 2017	Atici, Yunus; Aydin, Canan Gonen; Atici, Aysegul; Buyukkuscu, Mehmet Ozbey; Arikan, Yavuz; Balioglu, Mehmet Bulent. The effect of Kinesio taping on back pain in patients with Lenke Type 1 adolescent idiopathic scoliosis: A randomized controlled trial. Acta orthopaedica et traumatologica turcica, 2017, 51, 3, 191-196. https://dx.doi.org/10.1016/j.aott.2017.01.002.
#2206	Passias 2017	Passias, Peter G; Poorman, Gregory W; Jalai, Cyrus M; Line, Breton; Diebo, Bassel; Park, Paul; Hart, Robert; Burton, Douglas; Schwab, Frank; Lafage, Virginie; Bess, Shay; Errico, Thomas; International Spine Study Group. Outcomes of open staged corrective surgery in the setting of adult spinal deformity. The spine journal : official journal of the North American Spine Society, 2017, 17, 8, 1091-1099. https://dx.doi.org/10.1016/j.spinee.2017.03.012.
#2208	Kang 2017	Kang, Daniel G; Baldus, Christine; Glassman, Steven D; Shaffrey, Christopher I; Lurie, Jon D; Bridwell, Keith H. Neurologic Deficits Have a Negative Impact on Patient-Related Outcomes in Primary Presentation Adult Symptomatic Lumbar Scoliosis Surgical Treatment at One-Year Follow-up. Spine, 2017, 42, 7, 479-489. https://dx.doi.org/10.1097/BRS.0000000000001800.
#2210	Bourghli 2017	Bourghli, Anouar; Boissiere, Louis; Larrieu, Daniel; Vital, Jean-Marc; Yilgor, Caglar; Pellise, Ferran; Alanay, Ahmet; Acaroglu, Emre; Perez-Grueso, Francisco-Javier; Kleinstuck, Franck; Obeid, Ibrahim; European Spine Study Group. Lack of improvement in health-related quality of life (HRQOL) scores 6 months after surgery for adult spinal deformity (ASD) predicts high revision rate in the second postoperative year. European spine journal : official publication of the European Spine Society, the European Spinal Deformity Society, and the European Section of the Cervical Spine Research Society, 2017, 26, 8, 2160-2166. https://dx.doi.org/10.1007/s00586-017-5068-9.
#2222	Lee 2017	Lee, Jung Sub; Shin, Jong Ki; Goh, Tae Sik; Son, Seung Min; An, Sung Jin. Validation of the Korean version of the Spinal Appearance Questionnaire. Journal of back and musculoskeletal rehabilitation, 2017, 30, 6, 1203-1208. https://dx.doi.org/10.3233/BMR-150480.
#2235	Tsirikos 2017	Tsirikos, A I; Mataliotakis, G; Bounakis, N. Posterior spinal fusion for adolescent idiopathic scoliosis using a convex pedicle screw technique: a novel concept of deformity correction. The bone & joint journal, 2017, 99-B, 8, 1080-1087. https://dx.doi.org/10.1302/0301-620X.99B8.BJJ-2016-1351.R1.

#2238	Ward 2017	Ward, W Timothy; Friel, Nicole A; Kenkre, Tanya S; Brooks, Maria M; Londino, Joanne A; Roach, James W. SRS-22r Scores in Nonoperated Adolescent Idiopathic Scoliosis Patients With Curves Greater Than Forty Degrees. Spine, 2017, 42, 16, 1233-1240. https://dx.doi.org/10.1097/BRS.0000000000002004.
#2239	Liu 2017	Liu, Gang; Liu, Sen; Zuo, Yu-Zhi; Li, Qi-Yi; Wu, Zhi-Hong; Wu, Nan; Yu, Ke-Yi; Qiu, Gui-Xing. Recent Advances in Technique and Clinical Outcomes of Minimally Invasive Spine Surgery in Adult Scoliosis. Chinese medical journal, 2017, 130, 21, 2608-2615. https://dx.doi.org/10.4103/0366-6999.212688.
#2263	Bumpass 2017	Bumpass, David B; Lenke, Lawrence G; Gum, Jeffrey L; Shaffrey, Christopher I; Smith, Justin S; Ames, Christopher P; Bess, Shay; Neuman, Brian J; Klineberg, Eric; Mundis, Gregory M Jr; Schwab, Frank; Lafage, Virginie; Kim, Han Jo; Burton, Douglas C; Kebaish, Khaled M; Hostin, Richard; Lafage, Renaud; Kelly, Michael P; International Spine Study Group. Male sex may not be associated with worse outcomes in primary all-posterior adult spinal deformity surgery: a multicenter analysis. Neurosurgical focus, 2017, 43, 6, E9. https://dx.doi.org/10.3171/2017.9.FOCUS17475.
#2264	Than 2017	Than, Khoi D; Mummaneni, Praveen V; Bridges, Kelly J; Tran, Stacie; Park, Paul; Chou, Dean; La Marca, Frank; Uribe, Juan S; Vogel, Todd D; Nunley, Pierce D; Eastlack, Robert K; Anand, Neel; Okonkwo, David O; Kanter, Adam S; Mundis, Gregory M Jr. Complication rates associated with open versus percutaneous pedicle screw instrumentation among patients undergoing minimally invasive interbody fusion for adult spinal deformity. Neurosurgical focus, 2017, 43, 6, E7. https://dx.doi.org/10.3171/2017.8.FOCUS17479.
#2268	Misterska 2017	Misterska, Ewa; Glowacki, Jakub; Okret, Adam; Laurentowska, Maria; Glowacki, Maciej. Back and neck pain and function in females with adolescent idiopathic scoliosis: A follow-up at least 23 years after conservative treatment with a Milwaukee brace. PloS one, 2017, 12, 12, e0189358. https://dx.doi.org/10.1371/journal.pone.0189358.
#2273	Gur 2018	Gur, Gozde; Yakut, Yavuz; Grivas, Theo. The Turkish version of the Brace Questionnaire in brace-treated adolescents with idiopathic scoliosis. Prosthetics and orthotics international, 2018, 42, 2, 129-135. https://dx.doi.org/10.1177/0309364617690393.
#2279	Passias 2018	Passias, Peter G; Jalai, Cyrus M; Line, Breton G; Poorman, Gregory W; Scheer, Justin K; Smith, Justin S; Shaffrey, Christopher I; Burton, Douglas C; Fu, Kai-Ming G; Klineberg, Eric O; Hart, Robert A; Schwab, Frank; Lafage, Virginie; Bess, Shay; International Spine Study Group. Patient profiling can identify patients with adult spinal deformity (ASD) at risk for conversion from nonoperative to surgical treatment: initial steps to reduce ineffective ASD management. The spine journal : official journal of the North American Spine Society, 2018, 18, 2, 234-244. https://dx.doi.org/10.1016/j.spinee.2017.06.044.
#2280	Lonner 2018	Lonner, Baron S; Ren, Yuan; Yaszay, Burt; Cahill, Patrick J; Shah, Suken A; Betz, Randal R; Samdani, Amer F; Shufflebarger, Harry L; Newton, Peter O. Evolution of Surgery for Adolescent Idiopathic Scoliosis Over 20 Years: Have Outcomes Improved?. Spine, 2018, 43, 6, 402-410. https://dx.doi.org/10.1097/BRS.0000000000002332.

#2289	RezaeiMotlagh 2018	Rezaei Motlagh, Fazel; Kamali, Mohammad; Babaee, Taher. Persian adaptation of Quality of Life Profile for Spinal Deformities questionnaire. Journal of back and musculoskeletal rehabilitation, 2018, 31, 1, 177-182. https://dx.doi.org/10.3233/BMR-169775.
#2290	Moke 2018	Moke, Lieven; Severijns, Pieter; Schelfaut, Sebastiaan; Van de Loock, Kristel; Hermans, Lore; Molenaers, Guy; Jonkers, Ilse; Scheys, Lennart. Performance on Balance Evaluation Systems Test (BESTest) Impacts Health-Related Quality of Life in Adult Spinal Deformity Patients. Spine, 2018, 43, 9, 637-646. https://dx.doi.org/10.1097/BRS.0000000000002390.
#2292	Zheng 2018	Zheng, Yu; Dang, Yini; Yang, Yan; Li, Huabo; Zhang, Lijie; Lou, Edmond H M; He, Chengqi; Wong, Mansang. Whether Orthotic Management and Exercise are Equally Effective to the Patients With Adolescent Idiopathic Scoliosis in Mainland China?: A Randomized Controlled Trial Study. Spine, 2018, 43, 9, E494-E503. https://dx.doi.org/10.1097/BRS.0000000000002412.
#2298	Lin 2018	Lin, Tao; Meng, Yichen; Li, Tangbo; Jiang, Heng; Gao, Rui; Zhou, Xuhui. Predictors of Postoperative Recovery Based on Health-Related Quality of Life in Patients after Degenerative Lumbar Scoliosis Surgery. World neurosurgery, 2018, 109, 101528275, e539-e545. https://dx.doi.org/10.1016/j.wneu.2017.10.015.
#2301	Martin-Buitrago 2018	Martin-Buitrago, Mar Perez; Pizones, Javier; Sanchez Perez-Grueso, Francisco Javier; Diaz Almiron, Mariana; Vila-Casademunt, Alba; Obeid, Ibrahim; Alanay, Ahmet; Kleinstuck, Frank; Acaroglu, Emre R; Pellise, Ferran; ESSG European Spine Study Group. Impact of Iliac Instrumentation on the Quality of Life of Patients With Adult Spine Deformity. Spine, 2018, 43, 13, 913-918. https://dx.doi.org/10.1097/BRS.0000000000002476.
#2304	Alanazi 2018	Alanazi, Malik H; Parent, Eric C; Dennett, Elizabeth. Effect of stabilization exercise on back pain, disability and quality of life in adults with scoliosis: a systematic review. European journal of physical and rehabilitation medicine, 2018, 54, 5, 647-653. https://dx.doi.org/10.23736/S1973-9087.17.05062-6.
#2305	Ng 2015	Ng, Bobby Kin Wah; Chau, Wai-Wang; Hui, Chak-Na; Cheng, Po-Yin; Wong, Chau-Yuet; Wang, Bin; Cheng, Jack Chun Yiu; Lam, Tsz Ping. HRQoL assessment by SRS-30 for Chinese patients with surgery for Adolescent Idiopathic Scoliosis (AIS). Scoliosis, 2015, 10, Suppl 2, S19. https://dx.doi.org/10.1186/1748-7161-10-S2-S19.
#2323	Makino 2015	Makino, Takahiro; Kaito, Takashi; Kashii, Masafumi; Iwasaki, Motoki; Yoshikawa, Hideki. Low back pain and patient-reported QOL outcomes in patients with adolescent idiopathic scoliosis without corrective surgery. SpringerPlus, 2015, 4, 101597967, 397. https://dx.doi.org/10.1186/s40064-015-1189-y.

#2331	Schreiber 2015	Schreiber, Sanja; Parent, Eric C; Moez, Elham Khodayari; Hedden, Douglas M; Hill, Doug; Moreau, Marc J; Lou, Edmond; Watkins, Elise M; Southon, Sarah C. The effect of Schroth exercises added to the standard of care on the quality of life and muscle endurance in adolescents with idiopathic scoliosis-an assessor and statistician blinded randomized controlled trial: "SOSORT 2015 Award Winner". Scoliosis, 2015, 10, 101271527, 24. https://dx.doi.org/10.1186/s13013-015-0048-5.
#2337	Rodrigues 2015	Rodrigues, Joao Bernardo Sancio Rocha; Saleme, Nathalia Ambrozim Santos; Batista, Jose Lucas Jr; Cardoso, Igor Machado; Jacob, Charbel Jr. Quality of life in patients submitted to surgical treatment of idiopathic scoliosis. Acta ortopedica brasileira, 2015, 23, 6, 287-9. https://dx.doi.org/10.1590/1413-785220152306115026.
#2341	Uehara 2018	Uehara, Masashi; Takahashi, Jun; Ikegami, Shota; Kuraishi, Shugo; Futatsugi, Toshimasa; Oba, Hiroki; Koseki, Michihiko; Kato, Hiroyuki. Mid-term results of computer-assisted skip pedicle screw fixation for patients with Lenke type 1 and 2 adolescent idiopathic scoliosis: A minimum five-year follow-up study. Journal of orthopaedic science : official journal of the Japanese Orthopaedic Association, 2018, 23, 2, 248-252. https://dx.doi.org/10.1016/j.jos.2017.11.011.
#2343	Kelly 2018	Kelly, Michael P; Kim, Han Jo; Ames, Christopher P; Burton, Douglas C; Carreon, Leah Yacat; Polly, David W Jr; Hostin, Richard; Jain, Amit; Gum, Jeffrey L; Lafage, Virginie; Schwab, Frank J; Shaffrey, Christopher I; Smith, Justin S; Bess, Shay; International Spine Study Group. Minimum Detectable Measurement Difference for Health-Related Quality of Life Measures Varies With Age and Disability in Adult Spinal Deformity: Implications for Calculating Minimal Clinically Important Difference. Spine, 2018, 43, 13, E790-E795. https://dx.doi.org/10.1097/BRS.0000000000002519.
#2353	Reid 2018	Reid, Daniel B C; Daniels, Alan H; Ailon, Tamir; Miller, Emily; Sciubba, Daniel M; Smith, Justin S; Shaffrey, Christopher I; Schwab, Frank; Burton, Douglas; Hart, Robert A; Hostin, Richard; Line, Breton; Bess, Shay; Ames, Christopher P; International Spine Study Group. Frailty and Health-Related Quality of Life Improvement Following Adult Spinal Deformity Surgery. World neurosurgery, 2018, 112, 101528275, e548-e554. https://dx.doi.org/10.1016/j.wneu.2018.01.079.
#2361	Misterska 2018	Misterska, Ewa; Glowacki, Jakub; Glowacki, Maciej; Okret, Adam. Long-term effects of conservative treatment of Milwaukee brace on body image and mental health of patients with idiopathic scoliosis. PloS one, 2018, 13, 2, e0193447. https://dx.doi.org/10.1371/journal.pone.0193447.
#2364	Mmopelwa 2018	Mmopelwa, Tiro; Ayhan, Selim; Yuksel, Selcen; Nabiyev, Vugar; Niyazi, Asli; Pellise, Ferran; Alanay, Ahmet; Sanchez Perez Grueso, Francisco Javier; Kleinstuck, Frank; Obeid, Ibrahim; Acaroglu, Emre; European Spine Study Group (ESSG). Analysis of factors affecting baseline SF-36 Mental Component Summary in Adult Spinal Deformity and its impact on surgical outcomes. Acta orthopaedica et traumatologica turcica, 2018, 52, 3, 179-184. https://dx.doi.org/10.1016/j.aott.2018.02.001.
#2365	Hu 2018	Hu, Zongshan; Zhao, Zhihui; Li, Jie; Tseng, Chang-Chun; Qiu, Yong; Cheng, Jack Chun-Yiu; Zhu, Zezhang; Liu, Zhen. Comparison of Clinical and Radiologic Outcome of Three-Dimensional Correction in Lenke 5C Curve: Uniplanar Versus Polyaxial Pedicle Screws. World neurosurgery, 2018, 114, 101528275, e729-e734. https://dx.doi.org/10.1016/j.wneu.2018.03.067.

#2370	Zhao 2018	Zhao, Yongfei; Liang, Yan; Mao, Keya. Radiographic and clinical outcomes following MIS-TLIF in patients with adult lumbar degenerative scoliosis. Journal of orthopaedic surgery and research, 2018, 13, 1, 93. https://dx.doi.org/10.1186/s13018-018-0764-7.
#2374	Ailon 2018	Ailon, Tamir; Smith, Justin S; Shaffrey, Christopher I; Soroceanu, Alex; Lafage, Virginie; Schwab, Frank; Burton, Douglas; Hart, Robert; Kim, Han Jo; Gum, Jeffrey; Hostin, Richard; Kelly, Michael P; Glassman, Steven; Scheer, Justin K; Bess, Shay; Ames, Christopher P; International Spine Study Group. Patients with Adult Spinal Deformity with Previous Fusions Have an Equal Chance of Reaching Substantial Clinical Benefit Thresholds in Health-Related Quality of Life Measures but Do Not Reach the Same Absolute Level of Improvement. World neurosurgery, 2018, 116, 101528275, e354-e361. https://dx.doi.org/10.1016/j.wneu.2018.04.204.
#2380	RappvanRoden 2018	Rapp van Roden, Elizabeth A; Richardson, Robert Tyler; Russo, Stephanie A; Rose, William C; Chafetz, Ross S; Gabos, Peter G; Shah, Suken A; Samdani, Amer F; Richards, James G. Shoulder Complex Mechanics in Adolescent Idiopathic Scoliosis and Their Relation to Patient-perceived Function. Journal of pediatric orthopedics, 2018, 38, 8, e446-e454. https://dx.doi.org/10.1097/BPO.0000000000001207.
#2381	Zimon 2018	Zimon, Michalina; Matusik, Edyta; Kapustka, Bartosz; Durmala, Jacek; Doroniewicz, Iwona; Wnuk, Bartosz. Conservative management strategies and stress level in children and adolescents with idiopathic scoliosis. Psychiatria polska, 2018, 52, 2, 355-369. https://dx.doi.org/10.12740/PP/OnlineFirst/68744.
#2401	Matamalas 2014	Matamalas, Antonia; Bago, Joan; D'Agata, Elisabetta; Pellise, Ferran. Body image in idiopathic scoliosis: a comparison study of psychometric properties between four patient-reported outcome instruments. Health and quality of life outcomes, 2014, 12, 101153626, 81. https://dx.doi.org/10.1186/1477-7525-12-81.
#2406	Fu 2016	Fu, Xin; Sun, Xiao-Lei; Harris, Jonathan A; Sheng, Sun-Ren; Xu, Hua-Zi; Chi, Yong-Long; Wu, Ai-Min. Long fusion correction of degenerative adult spinal deformity and the selection of the upper or lower thoracic region as the site of proximal instrumentation: a systematic review and meta-analysis. BMJ open, 2016, 6, 11, e012103. https://dx.doi.org/10.1136/bmjopen-2016-012103.
#2408	Hedayati 2018	Hedayati, Zahra; Ahmadi, Amir; Kamyab, Mojtaba; Babaee, Taher; Ganjavian, Mohammad Saleh. Effect of Group Exercising and Adjusting the Brace at Shorter Intervals on Cobb Angle and Quality of Life of Patients With Idiopathic Scoliosis. American journal of physical medicine & rehabilitation, 2018, 97, 2, 104-109. https://dx.doi.org/10.1097/PHM.0000000000000812.
#2422	Toombs 2018	Toombs, Courtney; Lonner, Baron; Shah, Suken; Samdani, Amer; Cahill, Patrick; Shufflebarger, Harry; Yaszay, Burt; Sponseller, Paul; Newton, Peter. Quality of Life Improvement Following Surgery in Adolescent Spinal Deformity Patients: A Comparison Between Scheuermann Kyphosis and Adolescent Idiopathic Scoliosis. Spine deformity, 2018, 6, 6, 676-683. https://dx.doi.org/10.1016/j.jspd.2018.04.009.

#2437	Swierkosz 2015	Swierkosz, Szymon; Nowak, Zbigniew. Low back pain in adolescents. An assessment of the quality of life in terms of qualitative and quantitative pain variables. Journal of back and musculoskeletal rehabilitation, 2015, 28, 1, 25-34. .
#2439	Theis 2015	Theis, Jennifer; Gerdhem, Paul; Abbott, Allan. Quality of life outcomes in surgically treated adult scoliosis patients: a systematic review. European spine journal : official publication of the European Spine Society, the European Spinal Deformity Society, and the European Section of the Cervical Spine Research Society, 2015, 24, 7, 1343-55. https://dx.doi.org/10.1007/s00586-014-3593-3.
#2446	Smith 2016	Smith, Justin S; Lafage, Virginie; Shaffrey, Christopher I; Schwab, Frank; Lafage, Renaud; Hostin, Richard; O'Brien, Michael; Boachie-Adjei, Oheneba; Akbarnia, Behrooz A; Mundis, Gregory M; Errico, Thomas; Kim, Han Jo; Protopsaltis, Themistocles S; Hamilton, D Kojo; Scheer, Justin K; Sciubba, Daniel; Ailon, Tamir; Fu, Kai-Ming G; Kelly, Michael P; Zebala, Lukas; Line, Breton; Klineberg, Eric; Gupta, Munish; Deviren, Vedat; Hart, Robert; Burton, Doug; Bess, Shay; Ames, Christopher P; International Spine Study Group. Outcomes of Operative and Nonoperative Treatment for Adult Spinal Deformity: A Prospective, Multicenter, Propensity-Matched Cohort Assessment With Minimum 2-
#2448	Ames 2016	Ames, Christopher; Gammal, Isaac; Matsumoto, Morio; Hosogane, Naobumi; Smith, Justin S; Protopsaltis, Themistocles; Yamato, Yu; Matsuyama, Yukihiro; Taneichi, Hiroshi; Lafage, Renaud; Ferrero, Emmanuelle; Schwab, Frank J; Lafage, Virginie. Geographic and Ethnic Variations in Radiographic Disability Thresholds: Analysis of North American and Japanese Operative Adult Spinal Deformity Populations. Neurosurgery, 2016, 78, 6, 793-801. https://dx.doi.org/10.1227/NEU.0000000000001184.
#2452	Simony 2016	Simony, Ane; Carreon, Leah Y; Andersen, Mikkel O. Reliability and Validity Testing of a Danish Translated Version of the Scoliosis Research Society Instrument-22 Revised (SRS-22R). Spine deformity, 2016, 4, 1, 16-21. https://dx.doi.org/10.1016/j.jspd.2015.06.006.
#2496	Cochran 1985	Cochran, T; Nachemson, A. Long-term anatomic and functional changes in patients with adolescent idiopathic scoliosis treated with the Milwaukee brace. Spine, 1985, 10, 2, 127-33. .
#2511	Mayo 1994	Mayo, N E; Goldberg, M S; Poitras, B; Scott, S; Hanley, J. The Ste-Justine Adolescent Idiopathic Scoliosis Cohort Study. Part III: Back pain. Spine, 1994, 19, 14, 1573-81. .
#2517	Albert 1995	Albert, T J; Purtill, J; Mesa, J; McIntosh, T; Balderston, R A. Health outcome assessment before and after adult deformity surgery. A prospective study. Spine, 1995, 20, 18, 2002-p2005. .

#2530	Payne 1997	Payne, W K 3rd; Ogilvie, J W; Resnick, M D; Kane, R L; Transfeldt, E E; Blum, R W. Does scoliosis have a psychological impact and does gender make a difference?. Spine, 1997, 22, 12, 1380-4. .
#2562	McDowell 2017	McDowell, Michael M; Tempel, Zachary J; Gandhoke, Gurpreet S; Khattar, Nicholas K; Hamilton, D Kojo; Kanter, Adam S; Okonkwo, David O. Evolution of Sagittal Imbalance Following Corrective Surgery for Sagittal Plane Deformity. Neurosurgery, 2017, 81, 1, 129-134. https://dx.doi.org/10.1093/neuros/nyx145.
#2568	Farrokhi 2017	Farrokhi, Majid Reza; Jamali, Mohammad; Gholami, Mehrnaz; Farrokhi, Farnaz; Hosseini, Khadijeh. Clinical and radiological outcomes after decompression and posterior fusion in patients with degenerative scoliosis. British journal of neurosurgery, 2017, 31, 5, 514-525. https://dx.doi.org/10.1080/02688697.2017.1317717.
#2595	Monticone 2017	Monticone, Marco; Ambrosini, Emilia; Rocca, Barbara; Foti, Calogero; Ferrante, Simona. Responsiveness and Minimal Important Changes of the Scoliosis Research Society-22 Patient Questionnaire in Subjects With Mild Adolescent and Moderate Adult Idiopathic Scoliosis Undergoing Multidisciplinary Rehabilitation. Spine, 2017, 42, 11, E672-E679. https://dx.doi.org/10.1097/BRS.0000000000001923.
#2596	Scaramuzzo 2017	Scaramuzzo, Laura; Giudici, Fabrizio; Bongetta, Daniele; Caboni, Eleonora; Minoia, Leone; Zagra, Antonino. Thoraco-lumbar selective fusion in adolescent idiopathic scoliosis with Lenke C modifier curves: clinical and radiographic analysis at 10-year follow-up. European spine journal : official publication of the European Spine Society, the European Spinal Deformity Society, and the European Section of the Cervical Spine Research Society, 2017, 26, Suppl 4, 514-523. https://dx.doi.org/10.1007/s00586-017-5152-1.
#2608	Perez-Grueso 2000	Perez-Grueso, F S; Fernandez-Baillo, N; Arauz de Robles, S; Garcia Fernandez, A. The low lumbar spine below Cotrel-Dubousset instrumentation: long-term findings. Spine, 2000, 25, 18, 2333-41. .
#2628	Sweet 2001	Sweet, F A; Lenke, L G; Bridwell, K H; Blanke, K M; Whorton, J. Prospective radiographic and clinical outcomes and complications of single solid rod instrumented anterior spinal fusion in adolescent idiopathic scoliosis. Spine, 2001, 26, 18, 1956-65. .
#2637	Koch 2001	Koch, K D; Buchanan, R; Birch, J G; Morton, A A; Gatchel, R J; Browne, R H. Adolescents undergoing surgery for idiopathic scoliosis: how physical and psychological characteristics relate to patient satisfaction with the cosmetic result. Spine, 2001, 26, 19, 2119-24. .

#2642	Freidel 2002	Freidel, Klaus; Petermann, Franz; Reichel, Dagmar; Steiner, Angela; Warschburger, Petra; Weiss, Hans R. Quality of life in women with idiopathic scoliosis. Spine, 2002, 27, 4, E87-91. .
#2645	Pratt 2002	Pratt, Roland K; Burwell, R Geoffrey; Cole, Ashley A; Webb, John K. Patient and parental perception of adolescent idiopathic scoliosis before and after surgery in comparison with surface and radiographic measurements. Spine, 2002, 27, 14, 1543-2. .
#2646	Gotze 2002	Gotze, Christian; Liljenqvist, Ulf R; Slomka, Astrid; Gotze, Hans Guenther; Steinbeck, Joern. Quality of life and back pain: outcome 16.7 years after Harrington instrumentation. Spine, 2002, 27, 13, 1456-4. .
#2651	Asher 2003	Asher, Marc; Min Lai, Sue; Burton, Doug; Manna, Barbara. Scoliosis research society-22 patient questionnaire: responsiveness to change associated with surgical treatment. Spine, 2003, 28, 1, 25628. .
#2652	Asher 2003	Asher, Marc; Min Lai, Sue; Burton, Doug; Manna, Barbara. Discrimination validity of the scoliosis research society-22 patient questionnaire: relationship to idiopathic scoliosis curve pattern and curve size. Spine, 2003, 28, 1, 27242. .
#2656	Merola 2002	Merola, Andrew A; Haher, Thomas R; Brkaric, Mario; Panagopoulos, Georgia; Mathur, Samir; Kohani, Omid; Lowe, Thomas G; Lenke, Larry G; Wenger, Dennis R; Newton, Peter O; Clements, David H 3rd; Betz, Randal R. A multicenter study of the outcomes of the surgical treatment of adolescent idiopathic scoliosis using the Scoliosis Research Society (SRS) outcome instrument. Spine, 2002, 27, 18, 2046-51. .
#2658	Rinella 2004	Rinella, Anthony; Lenke, Lawrence; Peelle, Michael; Edwards, Charles; Bridwell, Keith H; Sides, Brenda. Comparison of SRS questionnaire results submitted by both parents and patients in the operative treatment of idiopathic scoliosis. Spine, 2004, 29, 3, 303-10. .
#2659	Asher 2004	Asher, Marc; Lai, Sue Min; Burton, Doug; Manna, Barbara. The influence of spine and trunk deformity on preoperative idiopathic scoliosis patients' health-related quality of life questionnaire responses. Spine, 2004, 29, 8, 861-8. .

#2676	Glassman 2005	Glassman, Steven D; Berven, Sigurd; Bridwell, Keith; Horton, William; Dimar, John R. Correlation of radiographic parameters and clinical symptoms in adult scoliosis. Spine, 2005, 30, 6, 682-8. .
#2678	Climent 2005	Climent, Jose M; Bago, Juan; Ey, Anna; Perez-Grueso, Francisco J S; Izquierdo, Enrique. Validity of the Spanish version of the Scoliosis Research Society-22 (SRS-22) Patient Questionnaire. Spine, 2005, 30, 6, 705-9. .
#2681	Watanabe 2005	Watanabe, Kei; Hasegawa, Kazuhiro; Hirano, Toru; Uchiyama, Seiji; Endo, Naoto. Use of the scoliosis research society outcomes instrument to evaluate patient outcome in untreated idiopathic scoliosis patients in Japan: part I: comparison with nonscoliosis group: preliminary/limited review in a Japanese population. Spine, 2005, 30, 10, 1197-201. .
#2682	Watanabe 2005	Watanabe, Kei; Hasegawa, Kazuhiro; Hirano, Toru; Uchiyama, Seiji; Endo, Naoto. Use of the scoliosis research society outcomes instrument to evaluate patient outcome in untreated idiopathic scoliosis patients in Japan: part II: relation between spinal deformity and patient outcomes. Spine, 2005, 30, 10, 1202-5. .
#2683	Feise 2005	Feise, Ronald J; Donaldson, Sandra; Crowther, Edward R; Menke, J Michael; Wright, James G. Construction and validation of the scoliosis quality of life index in adolescent idiopathic scoliosis. Spine, 2005, 30, 11, 1310-5. .
#2702	Ohashi 2018	Ohashi, Masayuki; Watanabe, Kei; Hirano, Toru; Hasegawa, Kazuhiro; Katsumi, Keiichi; Shoji, Hirokazu; Tatsuki, Mizouchi; Endo, Naoto. Predicting Factors at Skeletal Maturity for Curve Progression and Low Back Pain in Adult Patients Treated Non-Operatively for Adolescent Idiopathic Scoliosis with Thoracolumbar/Lumbar Curves: A Mean 25-Year Follow-up. Spine, 2018, , 7610646, uxk, 7610649, . https://dx.doi.org/10.1097/BRS.0000000000002716.
#2735	Nauffal 2002	Nauffal, D; Domenech, R; Martinez Garcia, M A; Compte, L; Macian, V; Perpina, M. Noninvasive positive pressure home ventilation in restrictive disorders: outcome and impact on health-related quality of life. Respiratory medicine, 2002, 96, 10, 777-83. .
#2754	Liu 2009	Liu, Wei; Chen, Xiong-sheng; Jia, Lian-shun; Song, Dian-wen. The clinical features and surgical treatment of degenerative lumbar scoliosis: a review of 112 patients. Orthopaedic surgery, 2009, 1, 3, 176-83. https://dx.doi.org/10.1111/j.1757-7861.2009.00030.x.

#2756	Zhang 2017	Zhang, Hao-Cong; Zhang, Zi-Fang; Wang, Zhao-Han; Cheng, Jun-Yao; Wu, Yun-Chang; Fan, Yi-Ming; Wang, Tian-Hao; Wang, Zheng. Optimal Pelvic Incidence Minus Lumbar Lordosis Mismatch after Long Posterior Instrumentation and Fusion for Adult Degenerative Scoliosis. Orthopaedic surgery, 2017, 9, 3, 304-310. https://dx.doi.org/10.1111/os.12343.
#2791	Mannion 2018	Mannion, A F; Elfering, A; Bago, J; Pellise, F; Vila-Casademunt, A; Richner-Wunderlin, S; Domingo-Sabat, M; Obeid, I; Acaroglu, E; Alanay, A; Perez-Grueso, F S; Baldus, C R; Carreon, L Y; Bridwell, K H; Glassman, S D; Kleinstuck, F; European Spine Study Group (ESSG). Factor analysis of the SRS-22 outcome assessment instrument in patients with adult spinal deformity. European spine journal : official publication of the European Spine Society, the European Spinal Deformity Society, and the European Section of the Cervical Spine Research Society, 2018, 27, 3, 685-699. https://dx.doi.org/10.1007/s00586-017-5279-0.
#2821	Simony 2016	Simony, Ane; Carreon, Leah Y; Hansen, Karen Hoejmark; Andersen, Mikkel O. Reliability and Validity Testing of a Danish Translated Version of Spinal Appearance Questionnaire (SAQ) v 1.1. Spine deformity, 2016, 4, 2, 94-97. https://dx.doi.org/10.1016/j.jspd.2015.08.007.
#2867	Olafsson 1999	Olafsson, Y; Saraste, H; Ahlgren, R M. Does bracing affect self-image? A prospective study on 54 patients with adolescent idiopathic scoliosis. European spine journal : official publication of the European Spine Society, the European Spinal Deformity Society, and the European Section of the Cervical Spine Research Society, 1999, 8, 5, 402-5. .
#2888	Gabos 2004	Gabos, Peter G; Bojescul, John A; Bowen, J Richard; Keeler, Kathryn; Rich, Lillian. Long-term follow-up of female patients with idiopathic scoliosis treated with the Wilmington orthosis. The Journal of bone and joint surgery. American volume, 2004, 86, 9, 1891-9. .
#2906	Scheer 2017	Scheer, Justin K; Smith, Justin S; Schwab, Frank; Lafage, Virginie; Shaffrey, Christopher I; Bess, Shay; Daniels, Alan H; Hart, Robert A; Protopsaltis, Themistocles S; Mundis, Gregory M Jr; Sciubba, Daniel M; Ailon, Tamir; Burton, Douglas C; Klineberg, Eric; Ames, Christopher P; International Spine Study Group. Development of a preoperative predictive model for major complications following adult spinal deformity surgery. Journal of neurosurgery. Spine, 2017, 26, 6, 736-743. https://dx.doi.org/10.3171/2016.10.SPINE16197.
#2916	Lamontagne 2001	Lamontagne, L L; Hepworth, J T; Salisbury, M H. Anxiety and postoperative pain in children who undergo major orthopedic surgery. Applied nursing research : ANR, 2001, 14, 3, 119-24. .
#2919	Parsch 2002	Parsch, Dominik; Gartner, Vera; Brocai, Dario R C; Carstens, Claus; Schmitt, Holger. Sports activity of patients with idiopathic scoliosis at long-term follow-up. Clinical journal of sport medicine : official journal of the Canadian Academy of Sport Medicine, 2002, 12, 2, 34912. .

#2956	Cheung 2019	Cheung, Prudence Wing Hang; Wong, Carlos King Ho; Cheung, Jason Pui Yin. An Insight Into the Health-Related Quality of Life of Adolescent Idiopathic Scoliosis Patients Who Are Braced, Observed, and Previously Braced. Spine, 2019, 44, 10, E596-E605. https://dx.doi.org/10.1097/BRS.0000000000002918.
#2964	Thompson 2019	Thompson, J Y; Williamson, E M; Williams, M A; Heine, P J; Lamb, S E; ACTIvATeS Study Group. Effectiveness of scoliosis-specific exercises for adolescent idiopathic scoliosis compared with other non-surgical interventions: a systematic review and meta-analysis. Physiotherapy, 2019, 105, 2, 214-234. https://dx.doi.org/10.1016/j.physio.2018.10.004.
#2969	Prudnikova 2018	Prudnikova, Oksana G; Shchurova, Elena N. Surgical correction of severe spinal deformities using a staged protocol of external and internal techniques. International orthopaedics, 2018, 42, 2, 331-338. https://dx.doi.org/10.1007/s00264-017-3738-1.
#2981	Cheung 2018	Cheung, Prudence Wing Hang; Wong, Carlos King Ho; Lau, Sin Ting; Cheung, Jason Pui Yin. Responsiveness of the EuroQoL 5-dimension (EQ-5D) in adolescent idiopathic scoliosis. European spine journal : official publication of the European Spine Society, the European Spinal Deformity Society, and the European Section of the Cervical Spine Research Society, 2018, 27, 2, 278-285. https://dx.doi.org/10.1007/s00586-017-5330-1.
#2984	Yagi 2018	Yagi, Mitsuru; Fujita, Nobuyuki; Okada, Eijiro; Tsuji, Osahiko; Nagoshi, Narihito; Tsuji, Takashi; Asazuma, Takashi; Nakamura, Masaya; Matsumoto, Morio; Watanabe, Kota. Impact of Frailty and Comorbidities on Surgical Outcomes and Complications in Adult Spinal Disorders. Spine, 2018, 43, 18, 1259-1267. https://dx.doi.org/10.1097/BRS.0000000000002596.
#2989	Zaina 2018	Zaina, Fabio; Poggio, Martina; Donzelli, Sabrina; Negrini, Stefano. Can bracing help adults with chronic back pain and scoliosis? Short-term results from a pilot study. Prosthetics and orthotics international, 2018, 42, 4, 410-414. https://dx.doi.org/10.1177/0309364618757769.
#2990	Cawley 2018	Cawley, Derek T; Larrieu, Daniel; Fujishiro, Takashi; Kieser, David; Boissiere, Louis; Acaroglu, Emre; Alanay, Ahmet; Kleinstuck, Frank; Pellise, Ferran; Perez-Grueso, Francisco Sanchez; Vital, Jean-Marc; Gille, Olivier; Obeid, Ibrahim. NRS20: Combined Back and Leg Pain Score: A Simple and Effective Assessment of Adult Spinal Deformity. Spine, 2018, 43, 17, 1184-1192. https://dx.doi.org/10.1097/BRS.0000000000002633.
#2991	Yagci 2018	Yagci, Gozde; Ayhan, Cigdem; Yakut, Yavuz. Effectiveness of basic body awareness therapy in adolescents with idiopathic scoliosis: A randomized controlled study1. Journal of back and musculoskeletal rehabilitation, 2018, 31, 4, 693-701. https://dx.doi.org/10.3233/BMR-170868.

#2995	Liang 2018	Liang, Yan; Zhao, Yongfei; Wang, Tianhao; Zhu, Zhenqi; Liu, Haiying; Mao, Keya. Precision Treatment of Adult Lumbar Degenerative Scoliosis Complicated by Lumbar Stenosis with the Use of Selective Nerve Root Block. World neurosurgery, 2018, 120, 101528275, e970-e975. https://dx.doi.org/10.1016/j.wneu.2018.08.205.
#2996	Langella 2018	Langella, Francesco; Villafañe, Jorge Hugo; Lafage, Virginie; Smith, Justin S; Shaffrey, Christopher; Kim, Han Jo; Burton, Douglas; Hostin, Richard; Bess, Shay; Ames, Christopher; Mundis, Gregory; Klineberg, Eric; Schwab, Frank; Lafage, Renault; Berjano, Pedro. Xipho-pubic angle (XPA) correlates with patient's reported outcomes in a population of adult spinal deformity: results from a multi-center cohort study. European spine journal : official publication of the European Spine Society, the European Spinal Deformity Society, and the European Section of the Cervical Spine Research Society, 2018, 27, 3, 670-677. https://dx.doi.org/10.1007/s00586-017-5460-5.
#2997	Yagi 2018	Yagi, Mitsuru; Ames, Christopher P; Keefe, Malla; Hosogane, Naobumi; Smith, Justin S; Shaffrey, Christopher I; Schwab, Frank; Lafage, Virginie; Shay Bess, R; Matsumoto, Morio; Watanabe, Kota; International Spine Study Group (ISSG). A cost-effectiveness comparisons of adult spinal deformity surgery in the United States and Japan. European spine journal : official publication of the European Spine Society, the European Spinal Deformity Society, and the European Section of the Cervical Spine Research Society, 2018, 27, 3, 678-684. https://dx.doi.org/10.1007/s00586-017-5274-5.
#3007	Sanders 2018	Sanders, Austin E; Andras, Lindsay M; Iantorno, Stephanie E; Hamilton, Anita; Choi, Paul D; Skaggs, David L. Clinically Significant Psychological and Emotional Distress in 32% of Adolescent Idiopathic Scoliosis Patients. Spine deformity, 2018, 6, 4, 435-440. https://dx.doi.org/10.1016/j.jspd.2017.12.014.
#3028	Liang 2018	Liang, Juping; Zhou, Xuan; Chen, Nan; Li, Xin; Yu, Hong; Yang, Yuqi; Song, Yuanyuan; Du, Qing. Efficacy of three-dimensionally integrated exercise for scoliosis in patients with adolescent idiopathic scoliosis: study protocol for a randomized controlled trial. Trials, 2018, 19, 1, 485. https://dx.doi.org/10.1186/s13063-018-2834-x.
#3029	Terheyden 2018	Terheyden, Jan Henrik; Wetterkamp, Mark; Gosheger, Georg; Lange, Tobias; Schulze Bovingloh, Albert; Schulte, Tobias L. Rasterstereography versus radiography for assessing shoulder balance in idiopathic scoliosis: A validation study relative to patients' self-image. Journal of back and musculoskeletal rehabilitation, 2018, 31, 6, 1049-1057. https://dx.doi.org/10.3233/BMR-170867.
#3030	Zhao 2018	Zhao, Jian; Yang, Mingyuan; Yang, Yiling; Chen, Ziqiang; Li, Ming. Proximal junctional kyphosis following correction surgery in the Lenke 5 adolescent idiopathic scoliosis patient. Journal of orthopaedic science : official journal of the Japanese Orthopaedic Association, 2018, 23, 5, 744-749. https://dx.doi.org/10.1016/j.jos.2018.05.010.
#3038	Wang 2019	Wang, Kai; Zhang, Can; Cheng, Cheng; Jian, Fengzeng; Wu, Hao. Radiographic and Clinical Outcomes following Combined Oblique Lumbar Interbody Fusion and Lateral Instrumentation for the Treatment of Degenerative Spine Deformity: A Preliminary Retrospective Study. BioMed research international, 2019, 2019, 101600173, 5672162. https://dx.doi.org/10.1155/2019/5672162.

#3041	Protopsaltis 2018	Protopsaltis, Themistocles S; Diebo, Bassel G; Lafage, Renaud; Henry, Jensen K; Smith, Justin S; Scheer, Justin K; Sciubba, Daniel M; Passias, Peter G; Kim, Han Jo; Hamilton, David K; Soroceanu, Alexandra; Klineberg, Eric O; Ames, Christopher P; Shaffrey, Christopher I; Bess, Shay; Hart, Robert A; Schwab, Frank J; Lafage, Virginie; International Spine Study Group. Identifying Thoracic Compensation and Predicting Reciprocal Thoracic Kyphosis and Proximal Junctional Kyphosis in Adult Spinal Deformity Surgery. Spine, 2018, 43, 21, 1479-1486. https://dx.doi.org/10.1097/BRS.0000000000002843.
#3046	Lee 2017	Lee, Chang-Hyun; Chung, Chun Kee; Sohn, Moon Jun; Kim, Chi Heon. Short Limited Fusion Versus Long Fusion With Deformity Correction for Spinal Stenosis With Balanced De Novo Degenerative Lumbar Scoliosis: A Meta-analysis of Direct Comparative Studies. Spine, 2017, 42, 19, E1126-E1132. https://dx.doi.org/10.1097/BRS.0000000000002306.
#3051	Diarbakerli 2018	Diarbakerli, Elias; Grauers, Anna; Danielsson, Aina; Gerdhem, Paul. Health-Related Quality of Life in Adulthood in Untreated and Treated Individuals with Adolescent or Juvenile Idiopathic Scoliosis. The Journal of bone and joint surgery. American volume, 2018, 100, 10, 811-817. https://dx.doi.org/10.2106/JBJS.17.00822.
#3058	Miyanji 2018	Miyanji, Firoz; Nasto, Luigi A; Bastrom, Tracey; Samdani, Amer F; Yaszay, Burt; Clements, David; Shah, Suken A; Lonner, Baron; Betz, Randal R; Shufflebarger, Harry L; Newton, Peter O. A Detailed Comparative Analysis of Anterior Versus Posterior Approach to Lenke 5C Curves. Spine, 2018, 43, 5, E285-E291. https://dx.doi.org/10.1097/BRS.0000000000002313.
#3069	Kwan 2018	Kwan, Mun Keong; Chiu, Chee Kidd; Tan, Pheng Hian; Chian, Xue Han; Ler, Xin Yi; Ng, Yun Hui; Ng, Sherwin Johan; Goh, Saw Huan; Chan, Chris Yin Wei. Radiological and clinical outcome of selective thoracic fusion for patients with Lenke 1C and 2C adolescent idiopathic scoliosis with a minimum follow-up of 2 years. The spine journal : official journal of the North American Spine Society, 2018, 18, 12, 2239-2246. https://dx.doi.org/10.1016/j.spinee.2018.05.007.
#3090	Hori 2019	Hori, Yusuke; Matsumura, Akira; Namikawa, Takashi; Kato, Minori; Takahashi, Shinji; Ohyama, Shoichiro; Ozaki, Tomonori; Yabu, Akito; Nakamura, Hiroaki. Does sagittal imbalance impact the surgical outcomes of short-segment fusion for lumbar spinal stenosis associated with degenerative lumbar scoliosis?. Journal of orthopaedic science : official journal of the Japanese Orthopaedic Association, 2019, 24, 2, 224-229. https://dx.doi.org/10.1016/j.jos.2018.10.005.
#3093	Verma 2019	Verma, Ravi; Lafage, Renaud; Scheer, Justin; Smith, Justin; Passias, Peter; Hostin, Richard; Ames, Christopher; Mundis, Gregory; Burton, Douglas; Kim, Han Jo; Bess, Shay; Klineberg, Eric; Schwab, Frank; Lafage, Virginie; International Spine Study Group. Improvement in Back and Leg Pain and Disability Following Adult Spinal Deformity Surgery: Study of 324 Patients With 2-year Follow-up and the Impact of Surgery on Patient-reported Outcomes. Spine, 2019, 44, 4, 263-269. https://dx.doi.org/10.1097/BRS.0000000000002815.
#3112	Passias 2018	Passias, Peter G; Jalai, Cyrus M; Lafage, Virginie; Poorman, Gregory W; Vira, Shaleen; Horn, Samantha R; Scheer, Justin K; Hamilton, D Kojo; Line, Breton G; Bess, Shay; Schwab, Frank J; Ames, Christopher P; Burton, Douglas C; Hart, Robert A; Klineberg, Eric O. Recovery Kinetics of Radiographic and Implant-Related Revision Patients Following Adult Spinal Deformity Surgery. Neurosurgery, 2018, 83, 4, 700-708. https://dx.doi.org/10.1093/neuros/nyx490.

#3115	Uribe 2018	Uribe, Juan S; Januszewski, Jacob; Wang, Michael; Anand, Neel; Okonkwo, David O; Mummaneni, Praveen V; Nguyen, Stacie; Zavatsky, Joseph; Than, Khoi; Nunley, Pierce; Park, Paul; Kanter, Adam S; La Marca, Frank; Fessler, Richard; Mundis, Gregory M; Eastlack, Robert K; Minimally Invasive Surgery Section of the International Spine Study Group. Patients with High Pelvic Tilt Achieve the Same Clinical Success as Those with Low Pelvic Tilt After Minimally Invasive Adult Deformity Surgery. Neurosurgery, 2018, 83, 2, 270-276. https://dx.doi.org/10.1093/neuros/nyx383.
#3127	Hosogane 2018	Hosogane, Naobumi; Ames, Christopher; Matsumoto, Morio; Yagi, Mitsuru; Matsuyama, Yukihiro; Taneichi, Hiroshi; Yamato, Yu; Takeuchi, Daisaku; Schwab, Frank; Shaffrey, Christopher; Smith, Justin S; Bess, Shay; Lafage, Virginie; International Spine Study Group. Ethnic Variations in Radiographic Parameters and SRS-22 Scores in Adult Spinal Deformity: A Comparison Between North American and Japanese Patients Above 50 Years of Age With Minimum 2-Year Follow-up. Clinical spine surgery, 2018, 31, 5, 216-221. https://dx.doi.org/10.1097/BSD.0000000000000610.
#3134	Ketenci 2018	Ketenci, Ismail Emre; Yanik, Hakan Serhat; Erdem, Sevki. The effect of upper instrumented vertebra level on cervical sagittal alignment in Lenke 1 adolescent idiopathic scoliosis. Orthopaedics & traumatology, surgery & research : OTSR, 2018, 104, 5, 623-629. https://dx.doi.org/10.1016/j.otsr.2018.06.003.
#3144	Scheer 2018	Scheer, Justin K; Oh, Taemin; Smith, Justin S; Shaffrey, Christopher I; Daniels, Alan H; Sciubba, Daniel M; Hamilton, D Kojo; Protopsaltis, Themistocles S; Passias, Peter G; Hart, Robert A; Burton, Douglas C; Bess, Shay; Lafage, Renaud; Lafage, Virginie; Schwab, Frank; Klineberg, Eric O; Ames, Christopher P; International Spine Study Group. Development of a validated computer-based preoperative predictive model for pseudarthrosis with 91% accuracy in 336 adult spinal deformity patients. Neurosurgical focus, 2018, 45, 5, E11. https://dx.doi.org/10.3171/2018.8.FOCUS18246.
#3155	Duramaz 2018	Duramaz, Altug; Yilmaz, Semra; Ziroglu, Nezih; Bursal Duramaz, Burcu; Kara, Tayfun. The effect of deformity correction on psychiatric condition of the adolescent with adolescent idiopathic scoliosis. European spine journal : official publication of the European Spine Society, the European Spinal Deformity Society, and the European Section of the Cervical Spine Research Society, 2018, 27, 9, 2233-2240. https://dx.doi.org/10.1007/s00586-018-5639-4.
#3158	Passias 2018	Passias, Peter G; Poorman, Gregory W; Lafage, Virginie; Smith, Justin; Ames, Christopher; Schwab, Frank; Shaffrey, Chris; Segreto, Frank A; Horn, Samantha R; Bortz, Cole A; Varlotta, Christopher G; Hockley, Aaron; Wang, Charles; Daniels, Alan; Neuman, Brian; Hart, Robert; Burton, Douglas; Javidan, Yashar; Line, Breton; LaFage, Renaud; Bess, Shay; Sciubba, Daniel; ISSG. Cervical Versus Thoracolumbar Spinal Deformities: A Comparison of Baseline Quality-of-Life Burden. Clinical spine surgery, 2018, 31, 10, 413-419. https://dx.doi.org/10.1097/BSD.0000000000000743.
#3160	Caronni 2019	Caronni, Antonio; Donzelli, Sabrina; Zaina, Fabio; Negrini, Stefano. The Italian Spine Youth Quality of Life questionnaire measures health-related quality of life of adolescents with spinal deformities better than the reference standard, the Scoliosis Research Society 22 questionnaire. Clinical rehabilitation, 2019, 33, 8, 1404-1415. https://dx.doi.org/10.1177/0269215519842246.
#3162	Louer 2019	Louer, Craig Jr; Yaszay, Burt; Cross, Madeline; Bartley, Carrie E; Bastrom, Tracey P; Shah, Suken A; Lonner, Baron; Cahill, Patrick J; Samdani, Amer; Upasani, Vidyadhar V; Newton, Peter O. Ten-Year Outcomes of Selective Fusions for Adolescent Idiopathic Scoliosis. The Journal of bone and joint surgery. American volume, 2019, 101, 9, 761-770. https://dx.doi.org/10.2106/JBJS.18.01013.

#3164	Makino 2019	Makino, Takahiro; Kaito, Takashi; Sakai, Yusuke; Takenaka, Shota; Yoshikawa, Hideki. Health-related Quality of Life and Postural Changes of Spinal Alignment in Female Adolescents Associated With Back Pain in Adolescent Idiopathic Scoliosis: A Prospective Cross-sectional Study. Spine, 2019, 44, 14, E833-E840. https://dx.doi.org/10.1097/BRS.0000000000002996.
#3170	Swamy 2019	Swamy, Ganesh; Lopatina, Elena; Thomas, Ken C; Marshall, Deborah A; Johal, Herman S. The cost effectiveness of minimally invasive spine surgery in the treatment of adult degenerative scoliosis: a comparison of transpsoas and open techniques. The spine journal : official journal of the North American Spine Society, 2019, 19, 2, 339-348. https://dx.doi.org/10.1016/j.spinee.2018.05.040.
#3171	Katayanagi 2019	Katayanagi, Junya; Iida, Takahiro; Hayamizu, Atsuki; Matsumoto, Kazuyuki; Ohyama, Yasumasa; Mine, Ken; Ozeki, Satoru. Effect of long spinal fusion including the pelvis on activities of daily living related to lumbar spinal function in adults with spinal deformity. Journal of orthopaedic science : official journal of the Japanese Orthopaedic Association, 2019, 24, 3, 409-414. https://dx.doi.org/10.1016/j.jos.2018.10.022.
#3172	Neal 2018	Neal, Chris J; Mandell, Kara; Tasikas, Ellen; Delaney, John J; Miller, Charles A; Schlaff, Cody D; Rosner, Michael K. Cost-effectiveness of adult spinal deformity surgery in a military healthcare system. Neurosurgical focus, 2018, 45, 6, E11. https://dx.doi.org/10.3171/2018.9.FOCUS18381.
#3174	Haddas 2019	Haddas, Ram; Lieberman, Isador H. The Change in Sway and Neuromuscular Activity in Adult Degenerative Scoliosis Patients Pre and Post Surgery Compared With Controls. Spine, 2019, 44, 15, E899-E907. https://dx.doi.org/10.1097/BRS.0000000000003009.
#3177	Ames 2019	Ames, Christopher P; Smith, Justin S; Pellise, Ferran; Kelly, Michael P; Gum, Jeffrey L; Alanay, Ahmet; Acaroglu, Emre; Perez-Grueso, Francisco Javier Sanchez; Kleinstuck, Frank S; Obeid, Ibrahim; Vila-Casademunt, Alba; Burton, Douglas C; Lafage, Virginie; Schwab, Frank J; Shaffrey, Christopher I; Bess, Shay; Serra-Burriel, Miquel; European Spine Study Group, International Spine Study Group. Development of Deployable Predictive Models for Minimal Clinically Important Difference Achievement Across the Commonly Used Health-related Quality of Life Instruments in Adult Spinal Deformity Surgery. Spine, 2019, 44, 16, 1144-1153.
#3180	Schreiber 2019	Schreiber, Sanja; Parent, Eric C; Hill, Doug L; Hedden, Douglas M; Moreau, Marc J; Southon, Sarah C. Patients with adolescent idiopathic scoliosis perceive positive improvements regardless of change in the Cobb angle - Results from a randomized controlled trial comparing a 6-month Schroth intervention added to standard care and standard care alone. SOSORT 2018 Award winner. BMC musculoskeletal disorders, 2019, 20, 1, 319. https://dx.doi.org/10.1186/s12891-019-2695-9.
#3188	Yagci 2019	Yagci, Gozde; Yakut, Yavuz. Core stabilization exercises versus scoliosis-specific exercises in moderate idiopathic scoliosis treatment. Prosthetics and orthotics international, 2019, 43, 3, 301-308. https://dx.doi.org/10.1177/0309364618820144.

#3191	Diarbakerli 2019	Diarbakerli, Elias; Grauers, Anna; Danielsson, Aina; Abbott, Allan; Gerdhem, Paul. Quality of Life in Males and Females With Idiopathic Scoliosis. Spine, 2019, 44, 6, 404-410. https://dx.doi.org/10.1097/BRS.0000000000002857.
#3193	Zhang 2019	Zhang, Yangpu; Tao, Luming; Hai, Yong; Yang, Jincai; Zhou, Lijin; Yin, Peng; Pan, Aixing; Liu, Chang. One-Stage Posterior Multiple-Level Asymmetrical Ponte Osteotomies Versus Single-Level Posterior Vertebral Column Resection for Severe and Rigid Adult Idiopathic Scoliosis: A Minimum 2-Year Follow-up Comparative Study. Spine, 2019, 44, 20, E1196-E1205. https://dx.doi.org/10.1097/BRS.0000000000003101.
#3197	Sharma 2019	Sharma, Akshay; Tanenbaum, Joseph E; Hogue, Olivia; Mehdi, Syed; Vallabh, Sagar; Hu, Emily; Benzel, Edward C; Steinmetz, Michael P; Savage, Jason W. Predicting Clinical Outcomes Following Surgical Correction of Adult Spinal Deformity. Neurosurgery, 2019, 84, 3, 733-740. https://dx.doi.org/10.1093/neuros/nyy190.
#3202	Yuksel 2019	Yuksel, Selcen; Ayhan, Selim; Nabiyev, Vugar; Domingo-Sabat, Montse; Vila-Casademunt, Alba; Obeid, Ibrahim; Perez-Grueso, Francisco Sanchez; Acaroglu, Emre; European Spine Study Group (ESSG). Minimum clinically important difference of the health-related quality of life scales in adult spinal deformity calculated by latent class analysis: is it appropriate to use the same values for surgical and nonsurgical patients?. The spine journal : official journal of the North American Spine Society, 2019, 19, 1, 71-78. https://dx.doi.org/10.1016/j.spinee.2018.07.005.
#3203	Raad 2019	Raad, Micheal; Jain, Amit; Huang, Mitchell; Skolasky, Richard L; Sciubba, Daniel M; Kebaish, Khaled M; Neuman, Brian J. Validity and responsiveness of PROMIS in adult spinal deformity: The need for a self-image domain. The spine journal : official journal of the North American Spine Society, 2019, 19, 1, 50-55. https://dx.doi.org/10.1016/j.spinee.2018.07.014.
#3208	Wong 2019	Wong, Carlos King Ho; Cheung, Prudence Wing Hang; Luo, Nan; Lin, Jiaer; Cheung, Jason Pui Yin. Responsiveness of EQ-5D Youth version 5-level (EQ-5D-5L-Y) and 3-level (EQ-5D-3L-Y) in Patients With Idiopathic Scoliosis. Spine, 2019, 44, 21, 1507-1514. https://dx.doi.org/10.1097/BRS.0000000000003116.
#3210	Yagci 2019	Yagci, Gozde; Demirkiran, Gokhan; Yakut, Yavuz. In-brace alterations of pulmonary functions in adolescents wearing a brace for idiopathic scoliosis. Prosthetics and orthotics international, 2019, 43, 4, 434-439. https://dx.doi.org/10.1177/0309364619839856.
#3211	Segreto 2019	Segreto, Frank A; Messina, James C; Doran, James P; Walker, Sarah E; Aylyarov, Alexandr; Shah, Neil V; Mixa, Patrick J; Ahmed, Natasha; Paltoo, Karen; Opare-Sem, Kwaku; Kaur, Harleen; Day, Louis M; Naziri, Qais; Paulino, Carl B; Scott, Claude B; Hesham, Khalid; Urban, William P; Diebo, Bassel G. Noncontact sports participation in adolescent idiopathic scoliosis: effects on parent-reported and patient-reported outcomes. Journal of pediatric orthopedics. Part B, 2019, 28, 4, 356-361. https://dx.doi.org/10.1097/BPB.0000000000000574.

#3215	Lin 2019	Lin, Tao; Meng, Yichen; Ji, Zhe; Jiang, Heng; Shao, Wei; Gao, Rui; Zhou, Xuhui. Extent of Depression in Juvenile and Adolescent Patients with Idiopathic Scoliosis During Treatment with Braces. World neurosurgery, 2019, 126, 101528275, e27-e32. https://dx.doi.org/10.1016/j.wneu.2019.01.095.
#3221	Than 2019	Than, Khoi D; Park, Paul; Tran, Stacie; Mundis, Gregory M; Fu, Kai-Ming; Uribe, Juan S; Okonkwo, David O; Nunley, Pierce D; Fessler, Richard G; Eastlack, Robert K; Kanter, Adam; Anand, Neel; LaMarca, Frank; Passias, Peter G; Mummaneni, Praveen V; International Spine Study Group. Analysis of Complications with Staged Surgery for Less Invasive Treatment of Adult Spinal Deformity. World neurosurgery, 2019, 126, 101528275, e1337-e1342. https://dx.doi.org/10.1016/j.wneu.2019.03.090.
#3222	Ketenci 2018	Ketenci, Ismail Emre; Yanik, Hakan Serhat; Ulusoy, Ayhan; Demiroz, Serdar; Erdem, Sevki. Lowest Instrumented Vertebrae Selection for Posterior Fusion of Lenke 5C Adolescent Idiopathic Scoliosis: Can We Stop the Fusion One Level Proximal to Lower-end Vertebra?. Indian journal of orthopaedics, 2018, 52, 6, 657-664. https://dx.doi.org/10.4103/ortho.IJOrtho_579_16.
#3229	Piantoni 2018	Piantoni, Lucas; Tello, Carlos A; Remondino, Rodrigo G; Bersusky, Ernesto S; Menendez, Celica; Ponce, Corina; Quintana, Susana; Hekier, Felisa; Francheri Wilson, Ida A; Galaretto, Eduardo; Noel, Mariano A. Quality of life and patient satisfaction in bracing treatment of adolescent idiopathic scoliosis. Scoliosis and spinal disorders, 2018, 13, 101675716, 26. https://dx.doi.org/10.1186/s13013-018-0172-0.
#3236	Simony 2015	Simony, Ane; Hansen, Emil Jesper; Carreon, Leah Y; Christensen, Steen Bach; Andersen, Mikkel Osterheden. Health-related quality-of-life in adolescent idiopathic scoliosis patients 25 years after treatment. Scoliosis, 2015, 10, 101271527, 22. https://dx.doi.org/10.1186/s13013-015-0045-8.
#3247	Cheung 2016	Cheung, Prudence Wing Hang; Wong, Carlos King Ho; Samartzis, Dino; Luk, Keith Dip Kei; Lam, Cindy Lo Kuen; Cheung, Kenneth Man Chee; Cheung, Jason Pui Yin. Psychometric validation of the EuroQoL 5-Dimension 5-Level (EQ-5D-5L) in Chinese patients with adolescent idiopathic scoliosis. Scoliosis and spinal disorders, 2016, 11, 101675716, 19. https://dx.doi.org/10.1186/s13013-016-0083-x.
#3254	Scaramuzzo 2019	Scaramuzzo, Laura; Giudici, Fabrizio; Archetti, Marino; Minoia, Leone; Zagra, Antonino; Bongetta, Daniele. Clinical Relevance of Preoperative MRI in Adolescent Idiopathic Scoliosis: Is Hydromyelia a Predictive Factor of Intraoperative Electrophysiological Monitoring Alterations?. Clinical spine surgery, 2019, 32, 4, E183-E187. https://dx.doi.org/10.1097/BSD.0000000000000820.
#3259	Kontodimopoulos 2018	Kontodimopoulos, Nick; Damianou, Konstantia; Stamatopoulou, Eleni; Kalampokis, Anastasios; Loukos, Ioannis. Children's and parents' perspectives of health-related quality of life in newly diagnosed adolescent idiopathic scoliosis. Journal of orthopaedics, 2018, 15, 2, 319-323. https://dx.doi.org/10.1016/j.jor.2018.02.003.

#3270	Burger 2019	Burger, Marlette; Coetzee, Wilna; du Plessis, Lenka Z; Geldenhuys, Larissa; Joubert, Francois; Myburgh, Elzanne; van Rooyen, Chante; Vermeulen, Nicol. The effectiveness of Schroth exercises in adolescents with idiopathic scoliosis: A systematic review and meta-analysis. The South African journal of physiotherapy, 2019, 75, 1, 904. https://dx.doi.org/10.4102/sajp.v75i1.904.
#3274	Mac-Thiong 2019	Mac-Thiong, Jean-Marc; Remondino, Rodrigo; Joncas, J; Parent, Stefan; Labelle, Hubert. Long-term follow-up after surgical treatment of adolescent idiopathic scoliosis using high-density pedicle screw constructs: Is 5-year routine visit required?. European spine journal : official publication of the European Spine Society, the European Spinal Deformity Society, and the European Section of the Cervical Spine Research Society, 2019, 28, 6, 1296-1300. https://dx.doi.org/10.1007/s00586-019-05887-5.
#3288	Ishikawa 2019	Ishikawa, Yoshinori; Miyakoshi, Naohisa; Kobayashi, Takashi; Abe, Toshiki; Kijima, Hiroaki; Abe, Eiji; Shimada, Yoichi. Activities of daily living and patient satisfaction after long fusion for adult spinal deformity: a retrospective study. European spine journal : official publication of the European Spine Society, the European Spinal Deformity Society, and the European Section of the Cervical Spine Research Society, 2019, 28, 7, 1670-1677. https://dx.doi.org/10.1007/s00586-019-05893-7.
#3289	Fujishiro 2019	Fujishiro, Takashi; Boissiere, Louis; Cawley, Derek Thomas; Larrieu, Daniel; Gille, Olivier; Vital, Jean-Marc; Pellise, Ferran; Perez-Grueso, Francisco Javier Sanchez; Kleinstuck, Frank; Acaroglu, Emre; Alanay, Ahmet; Obeid, Ibrahim; European Spine Study Group, ESSG. Adult spinal deformity surgical decision-making score : Part 1: development and validation of a scoring system to guide the selection of treatment modalities for patients below 40 years with adult spinal deformity. European spine journal : official publication of the European Spine Society, the European Spinal Deformity Society, and the European Section of the Cervical Spine Research Society, 2019, 28, 7, 1652-
#3294	Uehara 2019	Uehara, Masashi; Takahashi, Jun; Ikegami, Shota; Kuraishi, Shugo; Futatsugi, Toshimasa; Oba, Hiroki; Takizawa, Takashi; Munakata, Ryo; Koseki, Michihiko; Kato, Hiroyuki. Correlation of Lower Instrumented Vertebra With Spinal Mobility and Health-related Quality of Life After Posterior Spinal Fusion for Adolescent Idiopathic Scoliosis. Clinical spine surgery, 2019, 32, 7, E326-E329. https://dx.doi.org/10.1097/BSD.0000000000000794.
#3314	Ames 2019	Ames, Christopher P; Smith, Justin S; Pellise, Ferran; Kelly, Michael; Gum, Jeffrey L; Alanay, Ahmet; Acaroglu, Emre; Perez-Grueso, Francisco Javier Sanchez; Kleinstuck, Frank S; Obeid, Ibrahim; Vila-Casademunt, Alba; Shaffrey, Christopher I Jr; Burton, Douglas C; Lafage, Virginie; Schwab, Frank J; Shaffrey, Christopher I Sr; Bess, Shay; Serra-Burriel, Miquel; European Spine Study Group; International Spine Study Group. Development of predictive models for all individual questions of SRS-22R after adult spinal deformity surgery: a step toward individualized medicine. European spine journal : official publication of the European Spine Society, the European Spinal Deformity
#3315	Zhang 2019	Zhang, Yuhao; Li, Xingwei. Treatment of bracing for adolescent idiopathic scoliosis patients: a meta-analysis. European spine journal : official publication of the European Spine Society, the European Spinal Deformity Society, and the European Section of the Cervical Spine Research Society, 2019, 28, 9, 2012-2019. https://dx.doi.org/10.1007/s00586-019-06075-1.
#3320	Zhou 2019	Zhou, Siyu; Li, Wei; Su, Tong; Du, Chengbo; Wang, Wei; Xu, Fei; Sun, Zhuoran; Li, Weishi. Does lumbar lordosis minus thoracic kyphosis predict the clinical outcome of patients with adult degenerative scoliosis?. Journal of orthopaedic surgery and research, 2019, 14, 1, 290. https://dx.doi.org/10.1186/s13018-019-1339-y.

#3324	Nazemi 2018	Nazemi, Alireza K; Gowd, Anirudh K; Vaccaro, Alexander R; Carmouche, Jonathan J; Behrend, Caleb J. Unilateral S2 alar-iliac screws for spinopelvic fixation. Surgical neurology international, 2018, 9, 101535836, 75. https://dx.doi.org/10.4103/sni.sni_460_17.
#3330	Karabulut 2019	Karabulut, Cem; Ayhan, Selim; Yuksel, Selcen; Nabiyev, Vugar; Vila-Casademunt, Alba; Pellise, Ferran; Alanay, Ahmet; Perez-Grueso, Francisco Javier Sanchez; Kleinstuck, Frank; Obeid, Ibrahim; Acaroglu, Emre; EUROPEAN SPINE STUDY GROUP. Adult Spinal Deformity Over 70 Years of Age: A 2-Year Follow-Up Study. International journal of spine surgery, 2019, 13, 4, 336-344. https://dx.doi.org/10.14444/6046.
#3335	Katz 2019	Katz, Austen D; Singh, Hardeep; Greenwood, Matthew; Cote, Mark; Moss, Isaac L. Clinical and Radiographic Evaluation of Multilevel Lateral Lumbar Interbody Fusion in Adult Degenerative Scoliosis. Clinical spine surgery, 2019, 32, 8, E386-E396. https://dx.doi.org/10.1097/BSD.0000000000000812.
#3343	Min 2018	Min, Kan; Jud, Lukas; Farshad, Mazda. Dual Sequential Short Anterior Correction in Double Major Adolescent Idiopathic Scoliosis. Spine deformity, 2018, 6, 5, 545-551. https://dx.doi.org/10.1016/j.jspd.2018.01.009.
#3346	Soliman 2018	Soliman, Hany Abdel Gawwad. Health-related Quality of Life and Body Image Disturbance of Adolescents With Severe Untreated Idiopathic Early-onset Scoliosis in a Developing Country. Spine, 2018, 43, 22, 1566-1571. https://dx.doi.org/10.1097/BRS.0000000000002686.
#3347	Thielsch 2018	Thielsch, Meinald T; Wetterkamp, Mark; Boertz, Patrick; Gosheger, Georg; Schulte, Tobias L. Reliability and validity of the Spinal Appearance Questionnaire (SAQ) and the Trunk Appearance Perception Scale (TAPS). Journal of orthopaedic surgery and research, 2018, 13, 1, 274. https://dx.doi.org/10.1186/s13018-018-0980-1.
#3356	Inami 2018	Inami, Satoshi; Moridaira, Hiroshi; Takeuchi, Daisaku; Ueda, Haruki; Shiba, Yo; Asano, Futoshi; Aoki, Hiromichi; Taneichi, Hiroshi. Postoperative Status of Global Sagittal Alignment With Compensation in Adult Spinal Deformity. Spine, 2018, 43, 23, 1631-1637. https://dx.doi.org/10.1097/BRS.0000000000002693.
#3357	Haddas 2018	Haddas, Ram; Lieberman, Isador H; Block, Andrew. The Relationship Between Fear-Avoidance and Neuromuscular Measures of Function in Patients With Adult Degenerative Scoliosis. Spine, 2018, 43, 23, E1412-E1421. https://dx.doi.org/10.1097/BRS.0000000000002719.

#3366	Meng 2017	Meng, Zeng-Dong; Li, Tian-Peng; Xie, Xu-Hua; Luo, Chong; Lian, Xing-Ye; Wang, Ze-Yu. Quality of life in adolescent patients with idiopathic scoliosis after brace treatment: A meta-analysis. Medicine, 2017, 96, 19, e6828. https://dx.doi.org/10.1097/MD.0000000000006828.
#3368	Misterska 2019	Misterska, Ewa; Glowacki, Jakub; Kolban, Maciej. Does rigid spinal orthosis carry more psychosocial implications than the flexible brace in AIS patients? A cross-sectional study. Journal of back and musculoskeletal rehabilitation, 2019, 32, 1, 101-109. https://dx.doi.org/10.3233/BMR-181121.
#3375	Cho 2018	Cho, Jae Hwan; Hwang, Chang Ju; Choi, Young Hyun; Lee, Dong-Ho; Lee, Choon Sung. Cervical sagittal alignment in patients with adolescent idiopathic scoliosis: is it corrected by surgery?. Journal of neurosurgery. Pediatrics, 2018, 21, 3, 292-301. https://dx.doi.org/10.3171/2017.8.PEDS17357.
#3381	Lendzion 2018	Lendzion, Maciej; Lukaszewicz, Ewa; Was, Jakub; Czaprowski, Dariusz. Self-evaluation of Trunk Aesthetics in Conservatively Treated Children and Adolescents with Idiopathic Scoliosis. Ortopedia, traumatologia, rehabilitacja, 2018, 20, 5, 371-382. https://dx.doi.org/10.5604/01.3001.0012.8273.
#3388	Banno 2019	Banno, Tomohiro; Arima, Hideyuki; Hasegawa, Tomohiko; Yamato, Yu; Togawa, Daisuke; Yoshida, Go; Yasuda, Tatsuya; Oe, Shin; Mihara, Yuki; Ushirozako, Hiroki; Matsuyama, Yukihiro. The Effect of Paravertebral Muscle on the Maintenance of Upright Posture in Patients With Adult Spinal Deformity. Spine deformity, 2019, 7, 1, 125-131. https://dx.doi.org/10.1016/j.jspd.2018.06.008.
#3394	Lonner 2019	Lonner, Baron S; Ren, Yuan; Bess, Shay; Kelly, Michael; Kim, Han Jo; Yaszay, Burt; Lafage, Virginie; Marks, Michelle; Miyanji, Firoz; Shaffrey, Christopher I; Newton, Peter O. Surgery for the Adolescent Idiopathic Scoliosis Patients After Skeletal Maturity: Early Versus Late Surgery. Spine deformity, 2019, 7, 1, 84-92. https://dx.doi.org/10.1016/j.jspd.2018.05.012.
#3396	Yeramaneni 2018	Yeramaneni, Samrat; Ames, Christopher P; Bess, Shay; Burton, Doug; Smith, Justin S; Glassman, Steven; Gum, Jeffrey L; Carreon, Leah; Jain, Amit; Zygourakis, Corinna; Avramis, Ioannis; Hostin, Richard; International Spine Study Group. Center variation in episode-of-care costs for adult spinal deformity surgery: results from a prospective, multicenter database. The spine journal : official journal of the North American Spine Society, 2018, 18, 10, 1829-1836. https://dx.doi.org/10.1016/j.spinee.2018.03.012.
#3411	Oe 2019	Oe, Shin; Togawa, Daisuke; Yamato, Yu; Yoshida, Go; Hasegawa, Tomohiko; Kobayashi, Sho; Yasuda, Tatsuya; Banno, Tomohiro; Arima, Hideyuki; Mihara, Yuki; Ushirozako, Hiroki; Matsuyama, Yukihiro. Comparison of Postoperative Outcomes According to Compensatory Changes of the Thoracic Spine Among Patients With a T1 Slope More Than 40degree. Spine, 2019, 44, 8, 579-587. https://dx.doi.org/10.1097/BRS.0000000000002880.

#3415	Riley 2018	Riley, Max S; Lenke, Lawrence G; Chapman, Todd M Jr; Sides, Brenda A; Blanke, Kathy M; Kelly, Michael P. Clinical and Radiographic Outcomes After Posterior Vertebral Column Resection for Severe Spinal Deformity with Five-Year Follow-up. The Journal of bone and joint surgery. American volume, 2018, 100, 5, 396-405. https://dx.doi.org/10.2106/JBJS.17.00597.
#3416	Faraj 2018	Faraj, Sayf S A; De Kleuver, Marinus; Vila-Casademunt, Alba; Holewijn, Roderick M; Obeid, Ibrahim; Acaroglu, Emre; Alanay, Ahmet; Kleinstuck, Frank; Perez-Grueso, Francisco S; Pellise, Ferran. Sagittal radiographic parameters demonstrate weak correlations with pretreatment patient-reported health-related quality of life measures in symptomatic de novo degenerative lumbar scoliosis: a European multicenter analysis. Journal of neurosurgery. Spine, 2018, 28, 6, 573-580. https://dx.doi.org/10.3171/2017.8.SPINE161266.
#3418	Ohashi 2019	Ohashi, Masayuki; Watanabe, Kei; Hirano, Toru; Hasegawa, Kazuhiro; Katsumi, Keiichi; Shoji, Hirokazu; Mizouchi, Tatsuki; Takahashi, Ikuko; Endo, Naoto. The Natural Course of Compensatory Lumbar Curves in Nonoperated Patients With Thoracic Adolescent Idiopathic Scoliosis. Spine, 2019, 44, 2, E89-E98. https://dx.doi.org/10.1097/BRS.0000000000002779.
#3424	Raad 2018	Raad, Micheal; Neuman, Brian J; Jain, Amit; Hassanzadeh, Hamid; Passias, Peter G; Klineberg, Eric; Mundis, Gregory M; Protopsaltis, Themistocles S; Miller, Emily K; Smith, Justin S; Lafage, Virginie; Hamilton, D Kojo; Bess, Shay; Kebaish, Khaled M; Sciubba, Daniel M; International Spine Study Group. The use of patient-reported preoperative activity levels as a stratification tool for short-term and long-term outcomes in patients with adult spinal deformity. Journal of neurosurgery. Spine, 2018, 29, 1, 68-74. https://dx.doi.org/10.3171/2017.10.SPINE17830.
#3446	Schlenzka 2019	Schlenzka, Dietrich; Ylikoski, Mauno; Poussa, Mikko; Yrjonen, Timo; Ristolainen, Leena. Concomitant low-grade isthmic L5-spondylolisthesis does not affect the course of adolescent idiopathic scoliosis. European spine journal : official publication of the European Spine Society, the European Spinal Deformity Society, and the European Section of the Cervical Spine Research Society, 2019, 28, 12, 3053-3065. https://dx.doi.org/10.1007/s00586-019-06089-9.
#3449	Zhang 2019	Zhang, Hao-Cong; Yu, Hai-Long; Yang, Hui-Feng; Sun, Peng-Fei; Wu, Hao-Tian; Zhan, Yang; Wang, Zheng; Xiang, Liang-Bi. Short-segment decompression/fusion versus long-segment decompression/fusion and osteotomy for Lenke-Silva type VI adult degenerative scoliosis. Chinese medical journal, 2019, 132, 21, 2543-2549. https://dx.doi.org/10.1097/CM9.0000000000000474.
#3453	Teles 2019	Teles, Alisson R; Ocay, Don Daniel; Bin Shebreen, Abdulaziz; Tice, Andrew; Saran, Neil; Ouellet, Jean A; Ferland, Catherine E. Evidence of impaired pain modulation in adolescents with idiopathic scoliosis and chronic back pain. The spine journal : official journal of the North American Spine Society, 2019, 19, 4, 677-686. https://dx.doi.org/10.1016/j.spinee.2018.10.009.
#3455	Yagi 2019	Yagi, Mitsuru; Hosogane, Naobumi; Fujita, Nobuyuki; Okada, Eijiro; Suzuki, Satoshi; Tsuji, Osahiko; Nagoshi, Narihito; Asazuma, Takashi; Tsuji, Takashi; Nakamura, Masaya; Matsumoto, Morio; Watanabe, Kota. Surgical risk stratification based on preoperative risk factors in adult spinal deformity. The spine journal : official journal of the North American Spine Society, 2019, 19, 5, 816-826. https://dx.doi.org/10.1016/j.spinee.2018.12.007.

#3458	Scott 2020	Scott, Eric L; Foxen-Craft, Emily; Caird, Michelle; Philliben, Riley; deSebour, Trevor; Currier, Emily; Voepel-Lewis, Terri. Parental Proxy PROMIS Pain Interference Scores are Only Modestly Concordant With Their Child's Scores: An Effect of Child Catastrophizing. The Clinical journal of pain, 2020, 36, 1, 44568. https://dx.doi.org/10.1097/AJP.0000000000000772.
#3467	Wang 2019	Wang, Hai; Li, Tao; Yuan, Wangshu; Zhang, Zheping; Wei, Jing; Qiu, Guixing; Shen, Jianxiong. Mental health of patients with adolescent idiopathic scoliosis and their parents in China: a cross-sectional survey. BMC psychiatry, 2019, 19, 1, 147. https://dx.doi.org/10.1186/s12888-019-2128-1.
#3470	Khoshhal 2019	Khoshhal, Yalda; Jalali, Maryam; Babaee, Taher; Ghandhari, Hassan; Gum, Jeffrey L. The Effect of Bracing on Spinopelvic Rotation and Psychosocial Parameters in Adolescents with Idiopathic Scoliosis. Asian spine journal, 2019, 13, 6, 1028-1035. https://dx.doi.org/10.31616/asj.2018.0307.
#3479	Xu 2019	Xu, Zhengkuan; Li, Fangcai; Chen, Gang; Chen, Qixin. Reassessment System and Staged Surgical Strategy with Minimally Invasive Techniques for Treatment of Severe Adult Spinal Deformities. World neurosurgery, 2019, 126, 101528275, e860-e868. https://dx.doi.org/10.1016/j.wneu.2019.03.001.
#3483	Anand 2019	Anand, Neel; Alayan, Alisa; Agrawal, Aniruddh; Kahwaty, Sheila; Nomoto, Edward; Khandehroo, Babak. Analysis of Spino-Pelvic Parameters and Segmental Lordosis with L5-S1 Oblique Lateral Interbody Fusion at the Bottom of a Long Construct in Circumferential Minimally Invasive Surgical Correction of Adult Spinal Deformity. World neurosurgery, 2019, 130, 101528275, e1077-e1083. https://dx.doi.org/10.1016/j.wneu.2019.07.091.
#3484	Watanabe 2020	Watanabe, Yuh; Yoshida, Go; Hasegawa, Tomohiko; Yamato, Yu; Togawa, Daisuke; Banno, Tomohiro; Oe, Shin; Arima, Hideyuki; Ushirozako, Hiroki; Yamada, Tomohiro; Murata, Hideyuki; Matsuyama, Yukihiro. Effect of Perioperative Mental Status on Health-related Quality of Life in Patients With Adult Spinal Deformities. Spine, 2020, 45, 2, E76-E82. https://dx.doi.org/10.1097/BRS.0000000000003186.
#3485	Durand 2019	Durand, Wesley M; Daniels, Alan H; Hamilton, David K; Passias, Peter G; Kim, Han Jo; Protopsaltis, Themistocles; Lafage, Virginie; Smith, Justin S; Shaffrey, Christopher; Gupta, Munish; Kelly, Michael P; Klineberg, Eric; Schwab, Frank; Burton, Doug; Bess, Shay; Ames, Christopher; Hart, Robert; International Spine Study Group. Younger Patients Are Differentially Affected by Stiffness-Related Disability Following Adult Spinal Deformity Surgery. World neurosurgery, 2019, 132, 101528275, e297-e304. https://dx.doi.org/10.1016/j.wneu.2019.08.169.
#3486	Watanabe 2020	Watanabe, Kei; Ohashi, Masayuki; Hirano, Toru; Katsumi, Keiichi; Mizouchi, Tatsuki; Tashi, Hideki; Minato, Keitaro; Hasegawa, Kazuhiro; Endo, Naoto. Health-Related Quality of Life in Nonoperated Patients With Adolescent Idiopathic Scoliosis in the Middle Years: A Mean 25-Year Follow-up Study. Spine, 2020, 45, 2, E83-E89. https://dx.doi.org/10.1097/BRS.0000000000003216.

#3491	Wong 2019	Wong, Arnold Y L; Samartzis, Dino; Cheung, Prudence W H; Cheung, Jason Pui Yin. How Common Is Back Pain and What Biopsychosocial Factors Are Associated With Back Pain in Patients With Adolescent Idiopathic Scoliosis?. Clinical orthopaedics and related research, 2019, 477, 4, 676-686. https://dx.doi.org/10.1097/CORR.0000000000000569.
#3493	Helenius 2019	Helenius, Linda; Diarbakerli, Elias; Grauers, Anna; Lastikka, Markus; Oksanen, Hanna; Pajulo, Olli; Loyttyniemi, Eliisa; Manner, Tuula; Gerdhem, Paul; Helenius, Ilkka. Back Pain and Quality of Life After Surgical Treatment for Adolescent Idiopathic Scoliosis at 5-Year Follow-up: Comparison with Healthy Controls and Patients with Untreated Idiopathic Scoliosis. The Journal of bone and joint surgery. American volume, 2019, 101, 16, 1460-1466. https://dx.doi.org/10.2106/JBJS.18.01370.
#3496	Park 2013	Park, Justin J; Carreon, Leah Y; Glassman, Steven D. Adult Lumbar Degenerative Scoliosis 40degree or Less: Outcomes of Surgical Treatment With Minimum 2-Year Follow-up. Spine deformity, 2013, 1, 3, 211-216. https://dx.doi.org/10.1016/j.jspd.2013.03.003.
#3502	Glassman 2013	Glassman, Steven D; Sucato, Daniel J; Carreon, Leah Y; Sanders, James O; Vitale, Michael G; Lenke, Lawrence G. Does Thoracic Hypokyphosis Matter in Lenke Type 1 Adolescent Idiopathic Scoliosis?. Spine deformity, 2013, 1, 1, 40-45. https://dx.doi.org/10.1016/j.jspd.2012.09.001.
#3512	Ledonio 2013	Ledonio, Charles G T; Polly, David W Jr; Crawford, Charles H 3rd; Duval, Sue; Smith, Justin S; Buchowski, Jacob M; Yson, Sharon C; Larson, A Noelle; Sembrano, Jonathan N; Santos, Edward R G. Adult Degenerative Scoliosis Surgical Outcomes: A Systematic Review and Meta-analysis. Spine deformity, 2013, 1, 4, 248-258. https://dx.doi.org/10.1016/j.jspd.2013.05.001.
#3514	McCarthy 2013	McCarthy, Ian; Hostin, Richard; O'Brien, Michael; Fleming, Neil; Ogola, Gerald; Kudyakov, Rustam; Richter, Kathleen; Saigal, Rajiv; Berven, Sigurd; Deviren, Vedat; Ames, Christopher; International Spine Study Group. Cost-Effectiveness of Surgical Treatment for Adult Spinal Deformity: A Comparison of Dollars per Quality of Life Improvement Across Health Domains. Spine deformity, 2013, 1, 4, 293-298. https://dx.doi.org/10.1016/j.jspd.2013.05.007.
#3517	Brewer 2013	Brewer, Paul; Berryman, Fiona; Baker, De; Pynsent, Paul; Gardner, Adrian. Influence of Cobb Angle and ISIS2 Surface Topography Volumetric Asymmetry on Scoliosis Research Society-22 Outcome Scores in Scoliosis. Spine deformity, 2013, 1, 6, 452-457. https://dx.doi.org/10.1016/j.jspd.2013.07.012.
#3519	Mesfin 2013	Mesfin, Addisu; Lenke, Lawrence G; Bridwell, Keith H; Jupitz, Jennifer M; Akhtar, Usman; Fogelson, Jeremy L; Hershman, Stuart; Kim, Han Jo; Koester, Linda A. Weight Change and Clinical Outcomes Following Adult Spinal Deformity Surgery in Overweight and Obese Patients. Spine deformity, 2013, 1, 5, 377-381. https://dx.doi.org/10.1016/j.jspd.2013.07.002.

#3529	Bastrom 2019	Bastrom, Tracey P; Bartley, Carrie E; Newton, Peter O; Harms Study Group. Patient-Reported SRS-24 Outcomes Scores After Surgery for Adolescent Idiopathic Scoliosis Have Improved Since the New Millennium. Spine deformity, 2019, 7, 6, 917-922. https://dx.doi.org/10.1016/j.jspd.2019.01.007.
#3558	Dunn 2018	Dunn, John; Henrikson, Nora B; Morrison, Caitlin C; Nguyen, Matt; Blasi, Paula R; Lin, Jennifer S. , 2018, , , . .
#3582	Glowacki 2013	Glowacki, Maciej; Misterska, Ewa; Adamczyk, Katarzyna; Latuszewska, Joanna. Prospective Assessment of Scoliosis-Related Anxiety and Impression of Trunk Deformity in Female Adolescents Under Brace Treatment. Journal of developmental and physical disabilities, 2013, 25, 2, 203-220. .
#3583	Glowacki 2013	Glowacki, Maciej; Misterska, Ewa; Adamczyk, Katarzyna; Latuszewska, Joanna. Changes in Scoliosis Patient and Parental Assessment of Mental Health in the Course of Cheneau Brace Treatment Based on the Strengths and Difficulties Questionnaire. Journal of developmental and physical disabilities, 2013, 25, 3, 325-342. .
#3585	Misterska 2014	Misterska, Ewa; Glowacki, Maciej; Adamczyk, Katarzyna; Jankowski, Roman. Patients' and Parents' Perceptions of Appearance in Scoliosis Treated with a Brace: A Cross-Sectional Analysis. Journal of child and family studies, 2014, 23, 7, 1163-1171. .
#3592	RezaeiMotlagh 2018	Rezaei Motlagh, Fazel; Pejam, Hamid; Babaee, Taher; Saeedi, Hassan; Hedayati, Zahra; Kamali, Mohammad. Persian adaptation of the Bad Sobernheim stress questionnaire for adolescent with idiopathic scoliosis. Disability and rehabilitation, 2018, , 9207179, a8i, 44566. https://dx.doi.org/10.1080/09638288.2018.1503728.
#3601	Kelly 2019	Kelly, Michael P; Kallen, Michael A; Shaffrey, Christopher I; Smith, Justin S; Burton, Douglas C; Ames, Christopher P; Lafage, Virginie; Schwab, Frank J; Kim, Han Jo; Klineberg, Eric O; Bess, Shay; International Spine Study Group. Examining the Patient-Reported Outcomes Measurement Information System versus the Scoliosis Research Society-22r in adult spinal deformity. Journal of neurosurgery. Spine, 2019, , 101223545, 44567. https://dx.doi.org/10.3171/2018.11.SPINE181014.
#3606	Aghdasi 2019	Aghdasi, Bayan; Bachmann, Keith R; Clark, DesRaj; Koldenhoven, Rachel; Sultan, Mark; George, Jose; Singla, Anuj; Abel, Mark F. Patient-reported Outcomes Following Surgical Intervention for Adolescent Idiopathic Scoliosis: A Systematic Review and Meta-Analysis. Clinical spine surgery, 2019, , 101675083, . https://dx.doi.org/10.1097/BSD.0000000000000822.

#3607	Ushirozako 2019	Ushirozako, Hiroki; Yoshida, Go; Hasegawa, Tomohiko; Yamato, Yu; Yasuda, Tatsuya; Banno, Tomohiro; Arima, Hideyuki; Oe, Shin; Mihara, Yuki; Yamada, Tomohiro; Ojima, Toshiyuki; Togawa, Daisuke; Matsuyama, Yukihiro. Impact of shift to the concave side of the C7-center sacral vertical line on de novo degenerative lumbar scoliosis progression in elderly volunteers. Journal of orthopaedic science : official journal of the Japanese Orthopaedic Association, 2019, , c5t, 9604934, . https://dx.doi.org/10.1016/j.jos.2019.03.007.
#3609	Sabou 2019	Sabou, Silviu; Lagaras, Apostolos; Verma, Rajat; Siddique, Irfan; Mohammad, Saeed. Comparative study of multilevel posterior interbody fusion plus anterior longitudinal ligament release versus classic multilevel posterior interbody fusion in the treatment of adult spinal deformities. Journal of neurosurgery. Spine, 2019, , 101223545, 44568. https://dx.doi.org/10.3171/2019.1.SPINE18754.
#3613	Kyrola 2019	Kyrola, Kati; Hakkinen, Arja H; Ylinen, Jari; Repo, Jussi P. Further validation of the Scoliosis Research Society (SRS-30) questionnaire among adult patients with degenerative spinal disorder. Disability and rehabilitation, 2019, , 9207179, a8i, 44567. https://dx.doi.org/10.1080/09638288.2019.1616327.
#3616	Yagi 2019	Yagi, Mitsuru; Fujita, Nobuyuki; Okada, Eijiro; Tsuji, Osahiko; Nagoshi, Narihito; Asazuma, Takashi; Nakamura, Masaya; Matsumoto, Morio; Watanabe, Kota. Clinical Outcomes, Complications, and Cost-effectiveness in Surgically Treated Adult Spinal Deformity Over 70 Years: A Propensity score-Matched Analysis. Clinical spine surgery, 2019, , 101675083, . https://dx.doi.org/10.1097/BSD.0000000000000842.
#3618	Fedorak 2019	Fedorak, Graham T; Larkin, Kevin; Heflin, John A; Xu, Julie; Hung, Man. Pediatric PROMIS is Equivalent to SRS-22 in Assessing Health Status in Adolescent Idiopathic Scoliosis. Spine, 2019, , 7610646, uxk, 7610649, . https://dx.doi.org/10.1097/BRS.0000000000003112.
#3620	Buell 2019	Buell, Thomas J; Chen, Ching-Jen; Nguyen, James H; Christiansen, Peter A; Murthy, Saikiran G; Buchholz, Avery L; Yen, Chun-Po; Shaffrey, Mark E; Shaffrey, Christopher I; Smith, Justin S. Surgical correction of severe adult lumbar scoliosis (major curves >= 75degree): retrospective analysis with minimum 2-year follow-up. Journal of neurosurgery. Spine, 2019, , 101223545, 44575. https://dx.doi.org/10.3171/2019.3.SPINE1966.
#3633	Watanabe 2019	Watanabe, Kei; Ohashi, Masayuki; Hirano, Toru; Katsumi, Keiichi; Mizouchi, Tatsuki; Tashi, Hideki; Minato, Keitaro; Hasegawa, Kazuhiro; Endo, Naoto. Health-Related Quality of Life in Non-Operated Patients with Adolescent Idiopathic Scoliosis in the Middle Years: a Mean 25-Year Follow-up Study. Spine, 2019, , 7610646, uxk, 7610649, . https://dx.doi.org/10.1097/BRS.0000000000003216.
#3647	Wibmer 2019	Wibmer, Christine; Trotsenko, Pawel; Gilg, Magdalena M; Leithner, Andreas; Sperl, Matthias; Saraph, Vinay. Observational retrospective study on socio-economic and quality of life outcomes in 41 patients with adolescent idiopathic scoliosis 5 years after bracing combined with physiotherapeutic scoliosis-specific exercises (PSSE). European spine journal : official publication of the European Spine Society, the European Spinal Deformity Society, and the European Section of the Cervical Spine Research Society, 2019, 28, 3, 611-618. https://dx.doi.org/10.1007/s00586-018-5746-2.

#3653	Chan 2019	Chan, Chris Yin Wei; Gani, Siti Mariam Abd; Lim, Min Yuen; Chiu, Chee Kidd; Kwan, Mun Keong. APSS-ASJ Best Clinical Research Award: Is There a Difference between Patients' and Parents' Perception of Physical Appearance in Adolescent Idiopathic Scoliosis?. Asian spine journal, 2019, 13, 2, 216-224. https://dx.doi.org/10.31616/asj.2018.0151.
#3656	DeLaRocha 2014	De La Rocha, Adriana; McClung, Anna; Sucato, Daniel J. Increased Body Mass Index Negatively Affects Patient Satisfaction After a Posterior Fusion and Instrumentation for Adolescent Idiopathic Scoliosis. Spine deformity, 2014, 2, 3, 208-213. https://dx.doi.org/10.1016/j.jspd.2013.12.005.
#3658	Weber 2014	Weber, Michael H; Mathew, Jacob E; Takemoto, Steven K; Na, Lumine H; Berven, Sigurd; Spinal Deformity Study Group. Postoperative Recovery Outcomes in Adult Scoliosis: A Prospective Multicenter Database With 5-Year Follow-Up. Spine deformity, 2014, 2, 3, 226-232. https://dx.doi.org/10.1016/j.jspd.2014.01.001.
#3659	Chen 2013	Chen, Antonia F; Bi, Wenzhu; Singhabahu, Dilrukshika; Londino, Joanne; Hohl, Justin; Ward, Maeve; Ward, W Timothy. Converting Scoliosis Research Society-24 to Scoliosis Research Society-22r in a Surgical-Range, Medical/Interventional Adolescent Idiopathic Scoliosis Patient Cohort. Spine deformity, 2013, 1, 2, 108-114. https://dx.doi.org/10.1016/j.jspd.2012.12.003.
#3660	Martin 2013	Martin, Christopher T; Skolasky, Richard L; Mohamed, Ahmed S; Kebaish, Khaled M. Preliminary Results of the Effect of Prophylactic Vertebroplasty on the Incidence of Proximal Junctional Complications After Posterior Spinal Fusion to the Low Thoracic Spine. Spine deformity, 2013, 1, 2, 132-138. https://dx.doi.org/10.1016/j.jspd.2013.01.005.
#3664	Brewer 2014	Brewer, Paul; Berryman, Fiona; Baker, De; Pynsent, Paul; Gardner, Adrian. Analysis of the Scoliosis Research Society-22 Questionnaire Scores: Is There a Difference Between a Child and Parent and Does Physician Review Change That?. Spine deformity, 2014, 2, 1, 34-39. https://dx.doi.org/10.1016/j.jspd.2013.08.006.
#3671	Lonner 2015	Lonner, Baron S; Toombs, Courtney S; Husain, Qasim M; Sponseller, Paul; Shufflebarger, Harry; Shah, Suken A; Samdani, Amer F; Betz, Randal R; Cahill, Patrick J; Yaszay, Burt; Newton, Peter O. Body Mass Index in Adolescent Spinal Deformity: Comparison of Scheuermann's Kyphosis, Adolescent Idiopathic Scoliosis, and Normal Controls. Spine deformity, 2015, 3, 4, 318-326. https://dx.doi.org/10.1016/j.jspd.2015.02.004.
#3689	Larson 2019	Larson, A Noelle; Baky, Fady; Ashraf, Ali; Baghdadi, Yaser M; Treder, Vickie; Polly, David W Jr; Yaszemski, Michael J. Minimum 20-Year Health-Related Quality of Life and Surgical Rates After the Treatment of Adolescent Idiopathic Scoliosis. Spine deformity, 2019, 7, 3, 417-427. https://dx.doi.org/10.1016/j.jspd.2018.09.003.

#3694	Rezaee 2019	Rezaee, Sedighe; Jalali, Maryam; Babaee, Taher; Kamali, Mohammad. Reliability and Concurrent Validity of a Culturally Adapted Persian Version of the Brace Questionnaire in Adolescents With Idiopathic Scoliosis. Spine deformity, 2019, 7, 4, 553-558. https://dx.doi.org/10.1016/j.jspd.2018.10.001.
#3695	Diebo 2019	Diebo, Bassel G; Segreto, Frank A; Solow, Maximillian; Messina, James C; Paltoo, Karen; Burekhovich, Steven A; Bloom, Lee R; Cautela, Frank S; Shah, Neil V; Passias, Peter G; Schwab, Frank J; Pasha, Saba; Lafage, Virginie; Paulino, Carl B. Adolescent Idiopathic Scoliosis Care in an Underserved Inner-City Population: Screening, Bracing, and Patient- and Parent-Reported Outcomes. Spine deformity, 2019, 7, 4, 559-564. https://dx.doi.org/10.1016/j.jspd.2018.11.014.
#3701	Ibaseta 2019	Ibaseta, Alvaro; Rahman, Rafa; Skolasky, Richard L; Reidler, Jay S; Kebaish, Khaled M; Neuman, Brian J. SRS-22r legacy scores can be accurately translated to PROMIS scores in adult spinal deformity patients. The spine journal : official journal of the North American Spine Society, 2019, , 101130732, . https://dx.doi.org/10.1016/j.spinee.2019.09.006.
#3702	Schoutens 2019	Schoutens, Carlijn; Cushman, Daniel M; McCormick, Zachary L; Conger, Aaron; van Royen, Barend J; Spiker, William R. Outcomes of Nonsurgical Treatments for Symptomatic Adult Degenerative Scoliosis: A Systematic Review. Pain medicine (Malden, Mass.), 2019, , 100894201, . https://dx.doi.org/10.1093/pm/pnz253.
#3704	Agarwal 2019	Agarwal, Nitin; Angriman, Federico; Goldschmidt, Ezequiel; Zhou, James; Kanter, Adam S; Okonkwo, David O; Passias, Peter G; Protopsaltis, Themistocles; Lafage, Virginie; Lafage, Renaud; Schwab, Frank; Bess, Shay; Ames, Christopher; Smith, Justin S; Shaffrey, Christopher I; Burton, Douglas; Hamilton, D Kojo; International Spine Study Group. Relationship between body mass index and sagittal vertical axis change as well as health-related quality of life in 564 patients after deformity surgery. Journal of neurosurgery. Spine, 2019, , 101223545, 44567. https://dx.doi.org/10.3171/2019.4.SPINE18485.
#3707	Pierce 2019	Pierce, Katherine E; Passias, Peter G; Alas, Haddy; Brown, Avery E; Bortz, Cole A; Lafage, Renaud; Lafage, Virginie; Ames, Christopher; Burton, Douglas C; Hart, Robert; Hamilton, Kojo; Kelly, Michael; Hostin, Richard; Bess, Shay; Klineberg, Eric; Line, Breton; Shaffrey, Christopher; Mummaneni, Praveen; Smith, Justin S; Schwab, Frank A; International Spine Study Group (ISSG). Does Patient Frailty Status Influence Recovery Following Spinal Fusion for Adult Spinal Deformity?: An Analysis of Patients With 3-Year Follow-up. Spine, 2019, , 7610646, uxk, 7610649, . https://dx.doi.org/10.1097/BRS.0000000000003288.
#3711	Cheung 2019	Cheung, Jason Pui Yin; Cheung, Prudence Wing Hang; Yeng, Wing Cheung; Chan, Lawrence Chi Kwan. Does Curve Regression Occur During Underarm Bracing in Patients with Adolescent Idiopathic Scoliosis?. Clinical orthopaedics and related research, 2019, , 0075674, dfy, . https://dx.doi.org/10.1097/CORR.0000000000000989.
#3713	Wang 2019	Wang, Yongqiang; Gao, Ang; Hudabardiy, Enhamujiang; Yu, Miao. Curve progression in de novo degenerative lumbar scoliosis combined with degenerative segment disease after short-segment fusion. European spine journal : official publication of the European Spine Society, the European Spinal Deformity Society, and the European Section of the Cervical Spine Research Society, 2019, , 9301980, b9y, . https://dx.doi.org/10.1007/s00586-019-06173-0.

#3717	Bae 2019	Bae, Seong Ho; Son, Seung Min; Shin, Won Chul; Goh, Tae Sik; Lee, Jung Sub. Validation of the Korean Version of the Body Image Disturbance Questionnaire-Scoliosis. Spine, 2019, , 7610646, uxk, 7610649, . https://dx.doi.org/10.1097/BRS.0000000000003328.
#3733	Ohashi 2020	Ohashi, Masayuki; Bastrom, Tracey P; Marks, Michelle C; Bartley, Carrie E; Newton, Peter O. The Benefits of Sparing Lumbar Motion Segments in Spinal Fusion for Adolescent Idiopathic Scoliosis are Evident at 10 years Postoperatively. Spine, 2020, , 7610646, uxk, 7610649, . https://dx.doi.org/10.1097/BRS.0000000000003373.
#3735	Newton 2020	Newton, Peter O; Ohashi, Masayuki; Bastrom, Tracey P; Bartley, Carrie E; Yaszay, Burt; Marks, Michelle C; Betz, Randal; Lenke, Lawrence G; Clements, David. Prospective 10-year follow-up assessment of spinal fusions for thoracic AIS: radiographic and clinical outcomes. Spine deformity, 2020, , 101603979, . https://dx.doi.org/10.1007/s43390-019-00015-1.
